# Pattern recognition of forced oscillation technique measurement results using deep learning can identify asthmatic patients more accurately than setting reference ranges

**DOI:** 10.1038/s41598-023-48042-3

**Published:** 2023-12-07

**Authors:** Chiune Funaita, Wakaba Furuie, Fumika Koike, Saki Oyama, Junji Endo, Yoshio Otani, Yuri Ichikawa, Minako Ito, Yoichi Nakamura, Keiko Komatuzaki, Akira Hirata, Yasunari Miyazaki, Yuki Sumi

**Affiliations:** 1 Clinical Information Applied Sciences, Tokyo Medical and Dental University, Tokyo, 113-8519 Japan; 2 Nishi-Shinbashi Hoken Center, Tokyo, 105-0003 Japan; 3 Ikebukuro Otani Clinic, Tokyo, 171-0021 Japan; 4 Medical Check-up Center, Yokohama City Minato Red Cross Hospital, Yokohama, 231-8682 Japan; 5 Medical Center for Allergic and Immune Diseases, Yokohama City Minato Red Cross Hospital, Yokohama, 231-8682 Japan; 6 Department of Respiratory Medicine, Tokyo Medical and Dental University, Tokyo, 113-8519 Japan; 7 Shibata Ekimae Hiratanaika Clinic, Niigata, 957-0055 Japan

**Keywords:** Respiration, Diagnostic markers, Machine learning, Asthma

## Abstract

No official clinical reference values have been established for MostGraph, which measures total respiratory resistance and reactance using the forced oscillation technique, complicating result interpretation. This study aimed to establish a reference range for MostGraph measurements and examine its usefulness in discriminating participants with asthma from controls (participants without any respiratory diseases). The study also aimed to investigate the effectiveness of deep learning in discriminating between the two aforementioned groups. To establish reference ranges, the MostGraph measurements of healthy controls (n = 215) were power-transformed to distribute the data more normally. After inverse transformation, the mean ± standard deviation × 2 of the transformed values were used to establish the reference ranges. The number of measured items outside the reference ranges was evaluated to discriminate patients with asthma (n = 941) from controls. Additionally, MostGraph measurements were evaluated using deep learning. Although reference ranges were established, patients with asthma could not be discriminated from controls. However, with deep learning, we could discriminate between the two groups with 78% accuracy. Therefore, deep learning, which considers multiple measurements as a whole, was more effective in interpreting MostGraph measurement results than use of reference ranges, which considers each result individually.

## Introduction

Forced oscillation technique (FOT) is a non-invasive method for measurement of respiratory resistance and reactance at various frequencies (commonly 4–50 Hz)^1^ using changes in pressure and airflow by applying sound waves from a loudspeaker during quiet breathing. As it does not require breathing effort, it may be applicable for patients who cannot follow instructions or patients with low lung function. FOT can measure different indices to those of spirometry and is thus expected to have new applications in respiratory diseases. An increasing number of reports of the usefulness of FOT in the evaluation and management of obstructive pulmonary diseases are being published^2–5^. MostGraph, the FOT device in accordance with the “Oscillometry Technical Standards”^1^, was developed in Japan. However, official reference ranges for MostGraph measurement results have not been established. One study in which an attempt was made to determine the MostGraph parameter reference values^6^ had a few limitations, such as that the age of the enrolled participants was > 46 years and participants with pulmonary diseases were not excluded. Thus, more than 14% of the study participants showed abnormal spirometry^6^. Furthermore, participants with abnormal chest radiographs were likely included. Consequently, standardized reference values still need to be determined.

In the present study, we first aimed to establish reference ranges for MostGraph measurements in healthy Japanese adults and to examine whether the established reference ranges are useful in differentiating between healthy controls and patients with asthma. Second, we aimed to investigate whether deep learning can discriminate between healthy controls and patients with asthma. Third, to support researchers conducting similar research, the Python programs used to define and train deep learning models, as well as simple HTML, CSS, and JavaScript programs on the web, will be made available to contribute to the medical field.

We chose patients with asthma and cough variant asthma (CVA) as the patient group with respiratory diseases, because asthma is sometimes difficult to diagnose objectively in a clinical setting. Patients with asthma present with coughing, wheezing, or dyspnea often show expiratory airflow limitation on pulmonary function tests, which may vary in intensity^7^. Although some patients with asthma have normal lung function, they may also exhibit abnormal FOT findings owing to airway remodeling, a structural change in the lungs^8^. For an objective diagnosis of asthma, either variable expiratory airflow limitation or airway hyperresponsiveness needs to be demonstrated^7^. Methods for demonstrating variable expiratory airflow limitations include the following: (i) a positive bronchodilator responsiveness (reversibility) test (the patient should have expiratory airflow limitation at the time of presentation); (ii) excessive variation in lung function between visits (to measure this, the patient needs to visit the clinic several times); and (iii) excessive variability in peak flow (the patient needs to visit the clinic several times as well)^7^. Airway hyper-responsiveness tests are rarely performed in Japan because they require an hour-long test with a doctor in attendance. Some asthma patients show increased Fractional exhaled Nitric Oxide (FeNO) levels; however, some people with allergic predispositions also show increased FeNO levels, even if they do not have asthma. CVA is a type of asthma in which the only symptom is cough, and it is especially difficult to diagnose objectively. Other respiratory diseases usually show abnormal chest radiography, spirometry, hematologic, or sputum findings, leading to a diagnosis. Physicians usually make a comprehensive CVA diagnosis and provide diagnostic treatment to alleviate the patient's suffering as soon as possible. As a non-specialist respiratory physician cannot always make an immediate CVA diagnosis in an outpatient setting, MostGraph measurements may be beneficial. We hypothesized that an increase in the odds ratio for the diagnosis of asthma/CVA using MostGraph measurements would be clinically beneficial.

Deep learning is a machine-learning method mimicking human neurons and neural networks. A neuron in the human brain receives signals from many other neurons via dendrites and dendritic branches and transmits to other neurons via axons. The artificial neuron receives signals from artificial neurons in the previous layer and transmits to artificial neurons in the next layer. Specifically, it is a mechanism in which the neuron output signals in the previous layer are weighted by applying coefficients, summing them, adding bias, and outputting them after passing through the activation function. Deep learning refers to multilayer neural networks, typically more than three layers. The loss is the difference between the final output from the deep learning network and the desired output, which is called the “ground truth” or “teaching data.” The learning process, called “backpropagation,” is repeated by changing the weighting coefficients and bias so that the error is close to zero. Theoretically, the multivariate analysis is equivalent to a one-layer neural network, except that its coefficients are calculated theoretically. Deep learning allows for more complex analysis by multiple layering. The performance and accuracy of deep learning was widely recognized at the ImageNet Large Scale Visual Recognition Challenge (ILSVRC) and speech recognition challenges in 2012. Thereafter, deep learning started to be widely used. The advantage of deep learning lies in its future expandability; one can aim for better outputs by changing the network configuration. As only a small number of people appear to be educated in this field of study, we made the source code public for other researchers to use in their own fields.

## Methods

This study was approved by the Medical Research Institute of the Tokyo Medical and Dental University (approval numbers: M2000-2047, M2019-054, M2018-093, M2019-156) and was conducted in accordance with the Declaration of Helsinki. Informed consent was obtained from all participants.

### Recruitment of healthy controls

Healthy participants over 16 years of age were recruited between September 2013 and May 2022 in the following three ways: (i) from among those attending a medical checkup at the Medical Check-up Center of Yokohama City Minato Red Cross Hospital. Healthy participants with normal chest radiographic findings in the previous annual medical checkup before the study entry were chosen. (ii) Healthy medical staff of the Medical Check-up Center of Yokohama City Minato Red Cross Hospital, with no respiratory diseases and normal chest radiographic findings during the annual medical checkup, were recruited. (iii) Healthy staff or students of Tokyo Medical and Dental University (TMDU), with no respiratory diseases and normal chest radiographic findings during the annual medical checkup, were additionally recruited.

The following were included in the study: (i) current smokers, ex-smokers, non-smokers, and participants with allergic rhinitis, if they showed no respiratory abnormalities; (ii) participants with diseases not related to the respiratory system, such as hypertension, hyperlipidemia, gout, or diabetes mellitus; (iii) participants with a medical history of childhood asthma, if they did not have demonstrable symptoms and were not taking any medications.

### Measurements performed for healthy controls

Healthy controls underwent FeNO, MostGraph, and spirometry measurements, in that order, to ensure that the previous measurements did not affect the subsequent ones. As FeNO is easily influenced by physical stimulation of airway epithelial cells during other testing modalities, it was performed first. Spirometry was performed last as participants were required to breathe deeply, which could cause bronchial constriction.

Those with elevated FeNO (> 36.8 ppb, which is the upper limit value for Japanese healthy controls^9,10^) or abnormal spirometry (forced vital capacity (FVC)/predicted FVC of < 80% or forced expiratory volume in 1 s (FEV1)/FVC of < 70%) results were excluded. Participants with pulmonary complications and those who could not undergo a thorough examination were also excluded. MostGraph measurements were performed thrice in accordance with the “Oscillometry Technical Standards”^1^ using pulse wave pressure, which is the general measurement method. Participants were familiar with this measurement method because they had been measured previously using white noise pressure, thrice. The best result of three measurements stimulated by the pulse wave pressure was adopted for subsequent analysis. The measurement results are greatly affected by factors such as breathing volume, body position, and tongue position^1^. As the measurements were repeated, the participants tended to become less nervous and relaxed, leading to better results. Therefore, the best result was chosen.

### Establishment of reference ranges based on the measurements of healthy controls

Males and females were analyzed separately. We obtained 24 MostGraph measurements for the average, inhaled, exhaled, and delta (Δ, difference between exhaled and inhaled) values of R5 (resistance at 5 Hz), R20 (resistance at 20 Hz), R5-R20, X5 (reactance at 5 Hz), Fres (resonant frequency), and ALX (reactance area). A power transform was used to distribute the data more normally. We applied the Yeo–Johnson transformation^11^ because it allowed for zero and negative input values. The sklearn.preprocessing.PowerTransformer^12^ function was applied to measured data. As the transformed values showed an almost normal distribution, the mean ± standard deviation (SD) × 2 were set for reference ranges of the transformed data. The inverse_transform^13^ method was used to obtain reference ranges for the measured values. Calculations and presentations were performed using the Python programs available at https://github.com/sumi-yuki/mostgraph/blob/main/YeoJohnson-male.py and https://github.com/sumi-yuki/mostgraph/blob/main/YeoJohnson-female.py.

### Recruitment of patients with asthma/cough variant asthma

Patients with asthma/CVA more than 16 years old were recruited retrospectively, from September 2013 to June 2021, in the following three ways: (i) from amongst those visiting the Allergy Center of Yokohama City Minato Red Cross Hospital; (ii) from amongst those visiting the Ikebukuro Otani Clinic; and (iii) from amongst those visiting the Shibata Ekimae Hiratanaika Clinic. For the inclusion, they were the first visit patients who were given final diagnosis of asthma based on the Japanese guidelines for adult asthma^14–17^. They had had no previous treatments for asthma and underwent MostGraph measurement via pulse wave pressure. The following were included in the study: (i) current smokers, ex-smokers, and non-smokers; (ii) participants with diseases not related to the respiratory system, such as hypertension, hyperlipidemia, gout, and diabetes mellitus. The patients were excluded if they had severe diseases like cancer, comorbid lung diseases, or infectious diseases including common cold or bronchitis. All data were collected at the initial presentation, before treatments were administered.

### Efficacy of reference ranges in distinguishing healthy controls and patients with asthma/cough variant asthma

Heat maps showing items outside the reference ranges for each participant were drawn for an overview. The number of measured items outside the reference range was used to distinguish between healthy controls and patients with asthma/CVA. Receiver operating characteristic (ROC) curves were drawn to determine cutoff values (the number outside the MostGraph reference ranges). Calculations and presentations were performed using Python, available at https://github.com/sumi-yuki/mostgraph/blob/main/YeoJohnson-male-linearregression.py and https://github.com/sumi-yuki/mostgraph/blob/main/YeoJohnson-female-linearregression.py.

### Efficacy of deep learning in distinguishing healthy controls and patients with asthma/cough variant asthma

**Figure 1:**
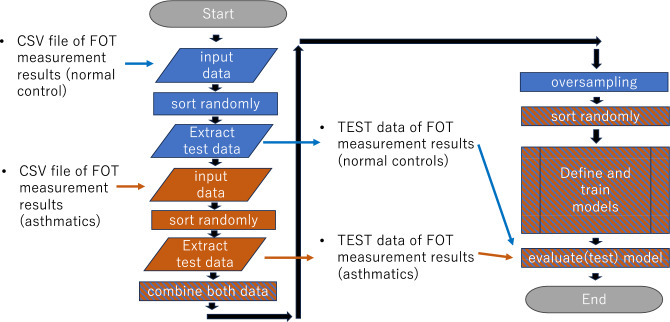
Flowchart of programs. First, test data from controls and patients were randomly extracted. The remaining data were used for training and validation. In order to balance the number of data, similar or identical data were created for oversampling, or large amounts of data were randomly discarded in the case of undersampling. The extracted test data were used to evaluate the model.

**Figure 2:**
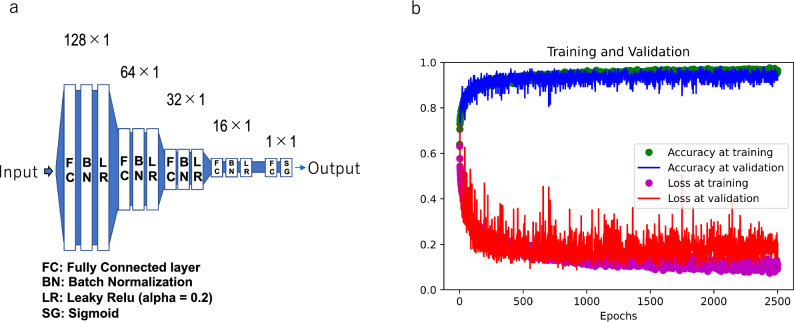
Structure of deep learning model and evaluation during training. (**a**) The structure of the five-layer deep learning model. Deep learning consisted of five fully connected layers (units = 128, 64, 32, 16, 1) with batch normalization and the LeakyReLU (alpha = 0.2) or sigmoid (final layer) activation function. (**b**) Accuracy and loss during training and validation. Representative accuracy and loss during training. As the training progressed, the loss decreased, and accuracy improved. The results were slightly different each time owing to the different random initializations. Overfitting was rarely observed during training.

To distinguish between healthy controls and patients with asthma/CVA, we applied deep learning to the MostGraph measurement results. To evaluate the models, we applied an iterated k-fold validation with shuffling. Briefly, 10 controls and 10 patients were randomly selected as test data (Fig. 1). The remaining data were used for training and validation. For the metrics, we demonstrated the accuracy of the diagnosis. The deep learning structure consisted of five fully connected layers with batch normalization and the LeakyReLU function (alpha = 0.2). A sigmoid function was used as the final activation function (Fig. 2a). Because the amount of asthmatic data was five times more than that of healthy controls, we applied oversampling to the healthy controls’ dataset in addition to weight balancing during training to handle imbalanced datasets for deep learning. The batch size was set to 32 and validation split was set to 0.05. The number of epochs was set to 2500. The reason is that up to 2500, overfitting was rarely observed during training, and a decrease in loss and an improvement in the accuracy rate were observed (Fig. 2b). The deep learning model was trained and estimated five times, and the final metric score was the average accuracy at a cutoff point of 0.5 obtained in each run.

MostGraph measurement reports consisted of the total, inhaled, exhaled, and delta values for R5, R20, R5-R20, X5, Fres, and ALX. The total value was the average of the inhaled and exhaled values, and the delta value was the difference between the inhaled and exhaled values. Considering multicollinearity, it should be possible to express with only 10 inhaled and exhaled data points for R5, R20, X5, Fres, and ALX. Therefore, we examined both the models. We also examined models in which age was added to the study items.

In addition, we compared three other models: male only, female only, and both male and female, adding sex as input data. The source code can be obtained from https://github.com/sumi-yuki/mostgraph/blob/main/deeplearning-mostgraph.py.

Other methods were also investigated. The number of test data was set to 20 in each group, oversampling the healthy controls’ dataset via SMOTE^11^, and the following metrics were assessed: sensitivity, specificity, accuracy and F1 score at the cutoff point defined by the maximum Youden's index, and AUC (area under the ROC curve) (Supplementary Fig. 3, Supplementary Table 1). The batch size was set to 32 and validation split was set to 0.05. The number of epochs was set to 1000. The source code can be obtained from https://github.com/sumi-yuki/mostgraph/blob/main/supplemental/deeplearning-5layer-smote.py. The deep learning model was trained and estimated six times, and the final metric score was the average of the metric scores (Supplementary Table 1). In addition to five-layer deep learning, we also evaluated a one-layer neural network (Supplementary Fig. 4, Supplementary Table 1) and logistic regression (Supplementary Fig. 5, Supplementary Table 1). The source codes are available from https://github.com/sumi-yuki/mostgraph/blob/main/supplemental/deeplearning-mono-smote.py and https://github.com/sumi-yuki/mostgraph/blob/main/supplemental/logistic-regression-smote.py. Undersampling methods for the asthmatic dataset were also investigated (Supplementary Fig. 6, Supplementary Table 1). The source code can be obtained from https://github.com/sumi-yuki/mostgraph/blob/main/supplemental/deeplearning-5layer-down.py.

### Ethical statement

This study was approved by the Medical Research Institute of the Tokyo Medical and Dental University (Approval Numbers: M2000-2047, M2019-054, M2018-093, M2019-156).

### Consent to participate

Written informed consent was obtained from all participants.

## Results

### Healthy controls

Of the recruited participants in the healthy control group, 215 (101 males, 114 females) were ultimately included in our study (Table 1).

**Table 1:** Demographic characteristics of healthy controls.

	Male participants (n = 101)	Female participants (n = 114)
Age (years)	39.3 ± 14.8	29.7 ± 10.8
Height (cm)	170.1 ± 5.6	158.7 ± 5.0
Weight (kg)	65.3 ± 11.3	50.8 ± 6.1
Smoking history (current/ex-smoking)	5 (5.0%)/15 (14.9%)	1 (0.88%)/4 (3.51%)
Pack-years of current or ex-smokers	323.3 ± 341.1	162.0 ± 149.9
FVC (L)	4.41 ± 0.53	3.30 ± 0.50
%FVC (%)	100.0 ± 10.5	101.9 ± 14.2
FEV_1_ (L)	3.64 ± 0.52	2.86 ± 0.42
FEV_1_/FVC (%)	82.6 ± 5.9	87.0 ± 6.79
R5 (cmH_2_O/L/s)	2.28 ± 1.02	2.69 ± 0.80
R5 exhale (cmH_2_O/L/s)	2.50 ± 1.22	3.01 ± 1.01
R5 inhale (cmH_2_O/L/s)	2.05 ± 0.92	2.36 ± 0.69
R5 exhale–inhale (cmH_2_O/L/s)	0.44 ± 0.71	0.66 ± 0.69
R20 (cmH_2_O/L/s)	2.17 ± 0.81	2.61 ± 0.72
R20 exhale (cmH_2_O/L/s)	2.30 ± 0.91	2.81 ± 0.83
R20 inhale (cmH_2_O/L/s)	2.04 ± 0.77	2.42 ± 0.66
R20 exhale–inhale (cmH_2_O/L/s)	0.26 ± 0.49	0.39 ± 0.45
R5-R20 (cmH_2_O/L/s)	0.11 ± 0.40	0.08 ± 0.47
R5-R20 exhale (cmH_2_O/L/s)	0.20 ± 0.50	0.21 ± 0.52
R5-R20 inhale (cmH_2_O/L/s)	0.01 ± 0.37	− 0.06 ± 0.47
R5-R20 exhale–inhale (cmH_2_O/L/s)	0.19 ± 0.34	0.27 ± 0.33
X5 (cmH_2_O/L/s)	− 0.18 ± 0.30	− 0.34 ± 0.35
X5 exhale (cmH_2_O/L/s)	− 0.12 ± 0.40	− 0.24 ± 0.45
X5 inhale (cmH_2_O/L/s)	− 0.24 ± 0.30	− 0.43 ± 0.34
X5 exhale–inhale (cmH_2_O/L/s)	0.12 ± 0.37	0.19 ± 0.38
Fres (Hz)	6.25 ± 1.72	6.88 ± 1.76
Fres exhale (Hz)	5.97 ± 2.24	6.41 ± 2.15
Fres inhale (Hz)	6.53 ± 1.73	7.34 ± 1.72
Fres exhale–inhale (Hz)	− 0.56 ± 2.00	− 0.93 ± 1.67
ALX (cmH_2_O/L/s·Hz)	0.89 ± 1.40	1.39 ± 1.56
ALX exhale (cmH_2_O/L/s·Hz)	0.79 ± 2.19	1.15 ± 2.12
ALX inhale (cmH_2_O/L/s·Hz)	0.98 ± 1.02	1.63 ± 1.39
ALX exhale–inhale (cmH_2_O/L/s·Hz)	− 0.18 ± 1.95	− 0.49 ± 1.78

Data are expressed as mean ± standard deviation. FVC, forced vital capacity; FEV_1_, forced expiratory volume in 1 s; R5, resistance at 5 Hz; R20, resistance at 20 Hz; X5, reactance at 5 Hz; Fres, resonant frequency; ALX, reactance area.

### Establishment of reference ranges based on measurements of healthy controls

**Figure 3:**
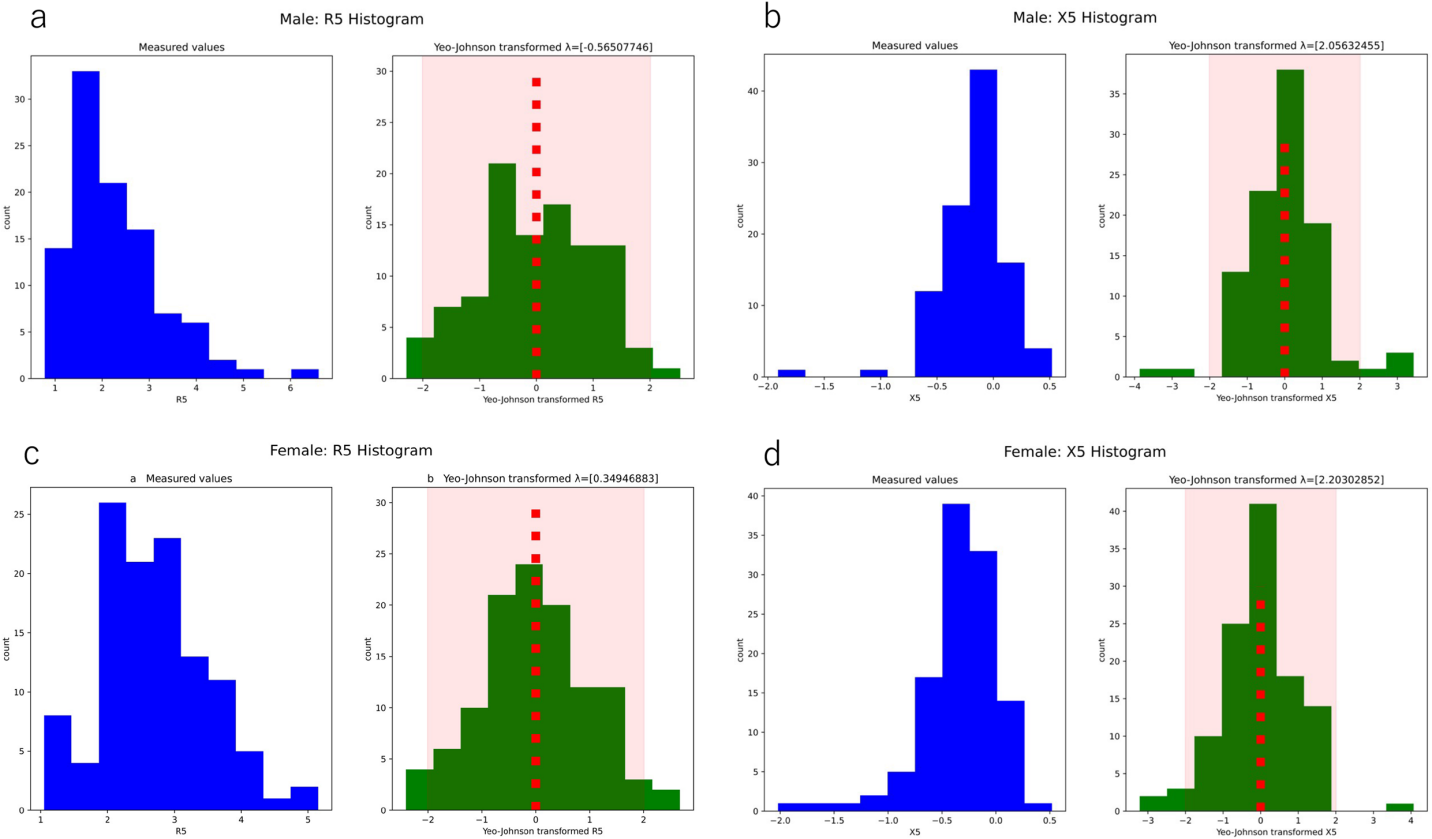
Histogram of MostGraph measurement values in healthy controls. (**a**) Histogram of R5 measured values and histograms of Yeo–Johnson power-transformed values of male participants. (**b**) Histogram of X5 measured values and histograms of Yeo–Johnson power-transformed values of male participants. (**c**) Histogram of R5 measured values and histograms of Yeo–Johnson power-transformed values of female participants. (**d**) Histogram of X5 measured values and histograms of Yeo–Johnson power-transformed values of female participants. Red dotted line indicates the mean, and pink square indicates mean ± 2 × standard deviation of the transformed value. All histograms are shown in Supplementary Fig. 1. R5, resistance at 5 Hz; X5, reactance at 5 Hz. These figures were calculated and constructed using the Python software. For male participants, “YeoJohnson-male.py” available at https://github.com/sumi-yuki/mostgraph/blob/main/YeoJohnson-male.py was used. For female participants, “YeoJohnson-female.py” available at https://github.com/sumi-yuki/mostgraph/blob/main/YeoJohnson-female.py was used.

The measured values did not show a normal distribution (Fig. 3); therefore, a power transform (Yeo-Johnson transformation^12^) was applied to make the data more normally distributed (Fig. 2). The mean ± 2 × SD of the transformed values were set as the reference range (Fig. 3). After inverse transformation, the reference ranges of the measured values were obtained (Table 2).

**Table 2:** Reference ranges of MostGraph measurement items established by healthy controls.

	Male participants (n = 101)	Female participants (n = 114)
R5 (cmH_2_O/L/s)	0.89–5.04	1.27–4.44
R5 exhale (cmH_2_O/L/s)	0.95–5.85	1.32–5.35
R5 inhale (cmH_2_O/L/s)	0.77–4.57	1.17–3.94
R5 exhale–inhale (cmH_2_O/L/s)	− 0.52–2.14	− 0.29–2.40
R20 (cmH_2_O/L/s)	1.03–4.34	1.37–4.25
R20 exhale (cmH_2_O/L/s)	1.07–4.74	1.38–4.71
R20 inhale (cmH_2_O/L/s)	0.94–4.12	1.33–4.00
R20 exhale–inhale (cmH_2_O/L/s)	− 0.62–1.33	− 0.23–1.51
R5-R20 (cmH_2_O/L/s)	− 0.61–0.99	− 0.73–1.14
R5-R20 exhale (cmH_2_O/L/s)	− 0.59–1.37	− 0.64–1.43
R5-R20 inhale (cmH_2_O/L/s)	− 0.67–0.8	− 0.90–0.97
R5-R20 exhale–inhale (cmH_2_O/L/s)	− 0.27–0.99	− 0.20–1.10
X5 (cmH_2_O/L/s)	− 0.85–0.29	− 1.16–0.19
X5 exhale (cmH_2_O/L/s)	− 0.98–0.38	− 1.29–0.33
X5 inhale (cmH_2_O/L/s)	− 0.91–0.30	− 1.20–0.17
X5 exhale–inhale (cmH_2_O/L/s)	− 0.69–0.78	− 0.62–0.86
Fres (Hz)	3.99–10.59	4.30–11.30
Fres exhale (Hz)	3.72–11.58	3.71–12.48
Fres inhale (Hz)	3.84–10.85	4.33–11.19
Fres exhale–inhale (Hz)	− 4.07–3.72	− 3.99–2.74
ALX (cmH_2_O/L/s·Hz)	− 0.06–4.05	0.01–5.73
ALX exhale (cmH_2_O/L/s·Hz)	− 0.09–3.71	− 0.12–7.32
ALX inhale (cmH_2_O/L/s·Hz)	− 0.11–4.62	− 0.01–5.70
ALX exhale–inhale (cmH_2_O/L/s·Hz)	− 2.40–4.02	− 2.93–3.67

R5, resistance at 5 Hz; R20, resistance at 20 Hz; X5, reactance at 5 Hz; Fres, resonant frequency; ALX, reactance area.

### Patients with asthma/cough variant asthma

Of the recruited participants in the patient group, 941 (294 males and 647 females) were included in our study (Table 3). The age distribution between healthy controls and patients with asthma/CVA was almost identical for males, whereas among females, the average age was lower in the healthy control group than that in the patient group.

**Table 3:** Demographics of patients with asthma and cough variant asthma.

	Male (n = 294)	Female (n = 647)
Age (years)	42.1 ± 16.0	42.5 ± 15.9
R5 (cmH_2_O/L/s)	2.77 ± 1.14	3.24 ± 1.53
R5 exhale (cmH_2_O/L/s)	3.19 ± 1.29	3.86 ± 1.75
R5 inhale (cmH_2_O/L/s)	2.72 ± 1.11	3.19 ± 1.37
R5 exhale–inhale (cmH_2_O/L/s)	0.60 ± 0.68	0.86 ± 1.00
R20 (cmH_2_O/L/s)	2.53 ± 0.86	3.04 ± 1.08
R20 exhale (cmH_2_O/L/s)	2.72 ± 0.97	3.30 ± 1.22
R20 inhale (cmH_2_O/L/s)	2.34 ± 0.81	2.77 ± 1.01
R20 exhale–inhale (cmH_2_O/L/s)	0.38 ± 0.46	0.53 ± 0.64
R5-R20 (cmH_2_O/L/s)	0.36 ± 0.47	0.39 ± 0.60
R5-R20 exhale (cmH_2_O/L/s)	0.37 ± 0.53	0.39 ± 0.73
R5-R20 inhale (cmH_2_O/L/s)	0.36 ± 0.51	0.39 ± 0.60
R5-R20 exhale–inhale (cmH_2_O/L/s)	0.23 ± 0.36	0.32 ± 0.47
X5 (cmH_2_O/L/s)	− 0.46 ± 0.63	− 0.60 ± 0.91
X5 exhale (cmH_2_O/L/s)	− 0.45 ± 0.87	− 0.64 ± 1.34
X5 inhale (cmH_2_O/L/s)	− 0.47 ± 0.56	− 0.57 ± 0.69
X5 exhale–inhale (cmH_2_O/L/s)	0.02 ± 0.73	− 0.06 ± 1.10
Fres (Hz)	8.00 ± 3.51	8.41 ± 3.74
Fres exhale (Hz)	7.95 ± 4.22	8.52 ± 4.83
Fres inhale (Hz)	8.04 ± 3.09	8.30 ± 3.11
Fres exhale–inhale (Hz)	− 0.09 ± 2.30	0.26 ± 3.18
ALX (cmH_2_O/L/s·Hz)	2.51 ± 5.42	3.42 ± 7.18
ALX exhale (cmH_2_O/L/s·Hz)	2.78 ± 8.00	4.09 ± 10.99
ALX inhale (cmH_2_O/L/s·Hz)	2.23 ± 4.82	2.76 ± 5.20
ALX exhale–inhale (cmH_2_O/L/s·Hz)	0.55 ± 7.55	1.33 ± 9.44

Data are expressed as mean ± standard deviation. Height and weight are not shown in the table because they were not recorded for some patients.

R5, resistance at 5 Hz; R20, resistance at 20 Hz; X5, reactance at 5 Hz; Fres, resonant frequency; ALX, reactance area.

### Efficacy of reference range in distinguishing healthy controls from patients with asthma/cough variant asthma

**Figure 4:**
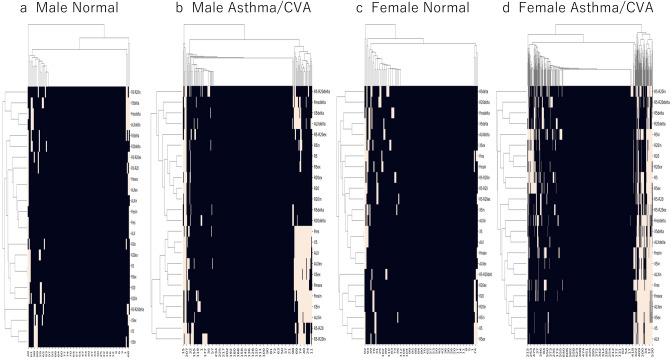
Heatmap clustered in a hierarchical manner of evaluated MostGraph measurement values by reference ranges. Beige color indicates items that are outside the reference ranges. (**a**) Heatmap of the male healthy controls. (**b**) Heatmap of the male asthmatic patients. (**c**) Heatmap of the female healthy controls. (**d**) Heatmap of the female asthmatic patients. The horizontal axis represents participants arranged by hierarchical clustering. The vertical axis indicates each MostGraph measurement item arranged by hierarchical clustering. These figures were constructed using Python. For male participants, “heatmap-male.py” available at https://github.com/sumi-yuki/mostgraph/blob/main/heatmap-male.py was used. For female participants, “heatmap-female.py” available at https://github.com/sumi-yuki/mostgraph/blob/main/heatmap-female.py was used.

**Figure 5:**
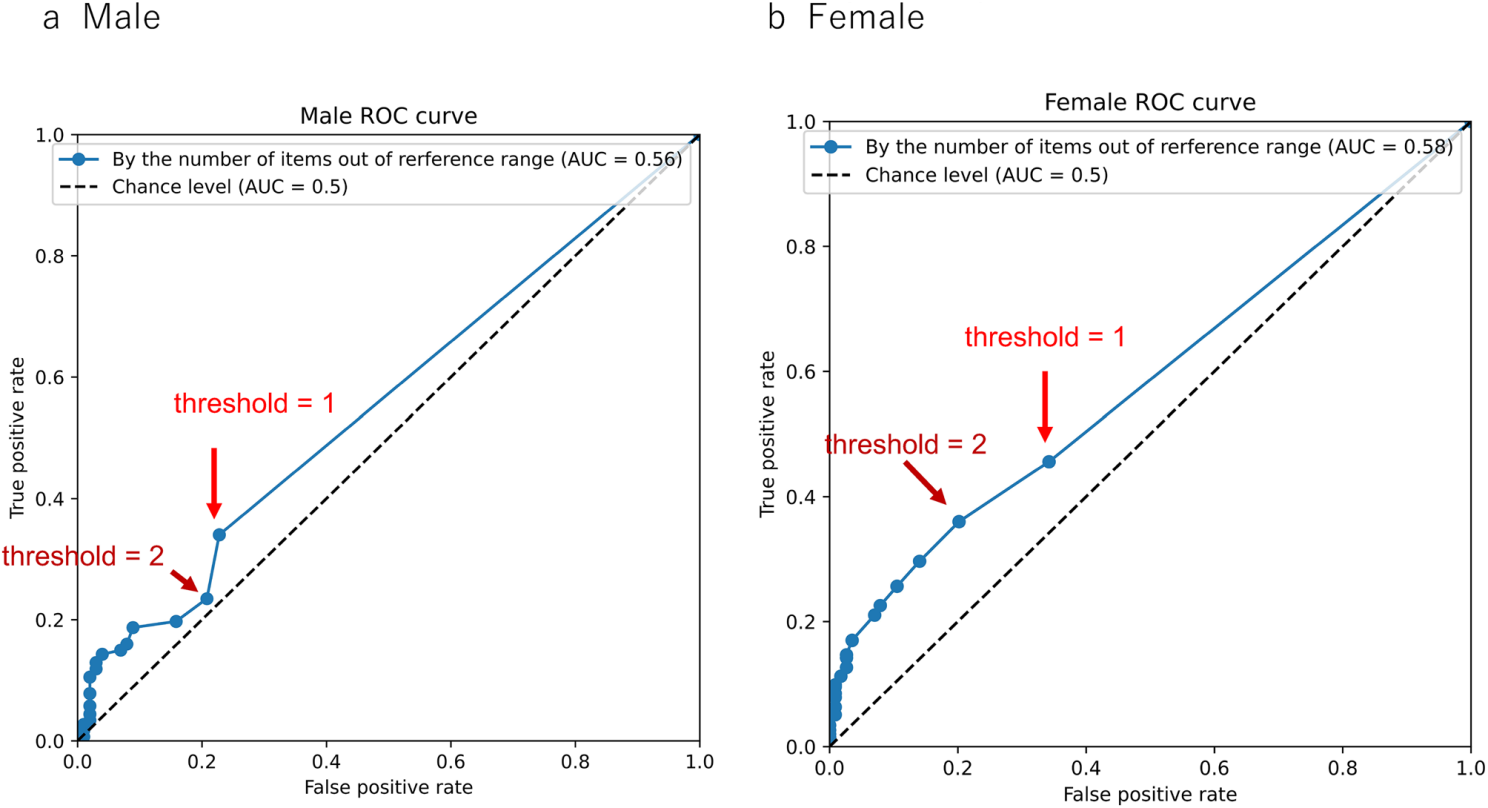
Receiver operating characteristic (ROC) curve to discriminate between healthy controls and patients with asthma by the number of abnormal MostGraph measured items. (a) Male participants. (b) Female participants. These figures were constructed using Python. For male participants, “heatmap-male.py” available at https://github.com/sumi-yuki/mostgraph/blob/main/heatmap-male.py was used. For female participants, “heatmap-female.py” available at https://github.com/sumi-yuki/mostgraph/blob/main/heatmap-female.py was used.

Heat maps of the MostGraph measurement results based on the reference range are shown in Fig. 4. These figures show that more items were outside the reference ranges in patients with asthma/CVA than in healthy controls. The ROC curve, which is used to discriminate between healthy controls and patients with asthma using the number of abnormal MostGraph-measured items, is shown in Fig. 5. Based on these results, discrimination between healthy controls and patients with asthma/CVA based on the number of abnormal items is difficult.

### Efficacy of deep learning in distinguishing healthy controls and patients with asthma/cough variant asthma

The diagnostic accuracies of the deep learning model are listed in Table 4. The accuracy did not differ when all MostGraph-measured items were used as inputs to the model compared to when only 10 measurement items were used as inputs. The results of deep learning, after adding age to the input, are presented in Table 5. However, even the addition of age did not improve diagnostic accuracy.

**Table 4:** Diagnostic accuracy of the deep learning model using all MostGraph-measured items as inputs to the model (by sex) and using only 10 measurement items as inputs (without age).

	All MostGraph-measured items		Values recorded for 10 items during exhalation and inhalation	
	Diagnostic accuracy for healthy controls	Diagnostic accuracy for patients with asthma	Diagnostic accuracy for healthy controls	Diagnostic accuracy for patients with asthma
Both sexes	0.64 ± 0.05	0.91 ± 0.07	0.71 ± 0.05	0.84 ± 0.11
Male participants	0.70 ± 0.09	0.63 ± 0.18	0.61 ± 0.21	0.64 ± 0.10
Female participants	0.76 ± 0.12	0.76 ± 0.10	0.79 ± 0.19	0.75 ± 0.06

**Table 5:** Diagnostic accuracy of the deep learning model using all MostGraph-measured items as inputs to the model (by sex) and using only 10 measurement items as inputs (including age).

	All MostGraph-measured items		10 inhaled and exhaled items	
	Diagnostic accuracy for healthy controls	Diagnostic accuracy for patients with asthma	Diagnostic accuracy for healthy controls	Diagnostic accuracy for patients with asthma
Both sexes	0.52 ± 0.08	0.84 ± 0.04	0.53 ± 0.12	0.85 ± 0.09
Male participants	0.48 ± 0.06	0.76 ± 0.15	0.36 ± 0.18	0.76 ± 0.14
Female participants	0.51 ± 0.11	0.84 ± 0.08	0.52 ± 0.13	0.85 ± 0.13

Based on these results, we adopted a single model for both males and females and input only the measurement results calculated during exhalation and inhalation for each item. We have published the MostGraph measurement results classifier on https://sumi-yuki.github.io/mostgraph/index.html and https://plaza.umin.ac.jp/~pulm/mostgraph/index.html.

Next, under identical conditions, we compared the diagnostic accuracy of diagnosis by deep learning and diagnosis based on the presence of one or more items outside the reference range (Table 6). The results indicated that deep learning outperformed the reference range and could discriminate between healthy controls and patients with asthma/CVA with an accuracy of approximately 78% (Table 6, Supplementary Table 1).

**Table 6:** Diagnostic accuracy of the deep learning model and that judged by the numbers of measured values that lie outside the reference range.

	Diagnostic accuracy for healthy controls	Diagnostic accuracy for patients with asthma
Deep learning	0.71 ± 0.05	0.84 ± 0.11
Reference ranges (male participants, cutoff point = 1)	0.77	0.38
Reference ranges (female participants, cutoff point = 1)	0.66	0.46

The five-layer deep learning model had a lower loss than the single-layer neural network models (Supplementary Fig. 3, 4), but the accuracy of discrimination was almost the same (Supplementary Table 1). The deep learning models yielded better results than the traditional logistic regression model (Supplementary Fig. 3, 5, Supplementary Table 1). Undersampling for asthma datasets yielded poor results (Supplementary Fig. 6, Supplementary Table 1).

## Discussion

MostGraph measurement results do not have official reference ranges; therefore, clinicians are often struggle with interpretation of results. We showed that reference ranges were too broad to use in the clinical setting, and that pattern recognition of measurement results by deep learning might be beneficial for clinical diagnosis. This is the first report to demonstrate the usefulness of modern deep learning techniques for interpretation of FOT results. Although some researchers^18–22^ have analyzed FOT results via machine learning, they used other techniques such as k-nearest neighbors, random forest, AdaBoost with decision trees, feature-based dissimilarity space classifier, support vector machines, radial basis function kernels, and Extreme Gradient Boosting. In a past study^18^, supervised machine learning techniques could classify patients with asthma without airway obstruction and those with airway obstruction by FOT with an accuracy similar to that in the present study. However, as we classified healthy participants and asthmatic participants even without obstruction, the conditions for separating the two were more difficult in this study and the number of participants was much larger.

In this study, we established the reference ranges for MostGraph measurements in healthy Japanese adults and examined whether the established reference ranges were useful in differentiating between healthy controls and patients with asthma. These reference ranges were narrower compared to those of a previous study^6^ on respiratory reactance (Table 7). Herein, we attempted to improve the predictions using age, height, and weight in the multiple regression analysis; however, this was not successful (Supplementary Fig. 2). One study revealed weak or absent correlation between MostGraph-measured values and age, height, or weight among both males and females^6^. In actual clinical settings, these reference ranges are too wide to be used, complicating differentiation between healthy controls and patients with asthma/CVA based on the number of abnormal items.

**Table 7:** Comparison of MostGraph-measurement reference ranges (2-SD away from mean) between this study and a previous study^4^.

	Male		Female	
	This study	Previous study	This study	Previous study
R5 (cmH_2_O/L/s)	~ 5.04	~ 4.77	~ 4.44	~ 5.87
R5 exhale (cmH_2_O/L/s)	~ 5.85	~ 5.08	~ 5.35	~ 6.58
R5 inhale (cmH_2_O/L/s)	~ 4.57	~ 4.7	~ 3.94	~ 5.45
R20 (cmH_2_O/L/s)	~ 4.34	~ 3.41	~ 4.25	~ 4.46
X5 (cmH_2_O/L/s)	~ −0.85	~ −1.79	~ −1.16	~ −2.25
Fres (Hz)	~ 10.59	~ 15	~ 11.30	~ 14.88
ALX (cmH_2_O/L/s·Hz)	~ 4.05	~ 17.2	~ 5.73	~ 20.52

SD, standard deviation; R5, resistance of 5 Hz; R20, resistance of 20 Hz; X5, reactance of 5 Hz; Fres, resonant frequency; ALX, reactance area.

The diagnostic accuracy for differentiation between healthy controls and patients with bronchial asthma/CVA by deep learning was approximately 78% in the present study. Even when separate models were created for males and females, the accuracy did not improve. The results of the MostGraph measurements were reported as exhaled, inhaled, total, and delta values for each item, where the total value was approximately the average of the exhaled and inhaled measurements, and the delta value was the difference between the exhaled and inhaled measurements. The diagnostic accuracy did not differ between the use of all reported items as inputs to the model and the use of only 10 measurements as inputs. In addition, adding age as input data did not improve diagnostic accuracy. Therefore, we used a single model for males and females, inputting sex and 10 MostGraph measurements, which were recorded during both exhalation and inhalation. Deep learning, which considers multiple items as a whole, was more effective in discriminating lung abnormalities than reference ranges, which evaluate each item individually.

We tried multiple methods for pattern recognition in this study: the conventional multiple logistic regression analysis and other neural network models. Accordingly, a complex, multi-layer deep learning model yielded lower loss (Supplementary Fig. 3) compared to a single-layer neural network (Supplementary Fig. 4). Because the final accuracy of discrimination was almost the same (Supplementary Table 1) owing to binary classification, these two methods do not differ in terms of practicality in this case. If a larger number of training datasets is used, other results may be obtained. The number of training datasets seems to be important because the undersampling method yielded poor results. Even when similar training data was given using methods such as SMOTE, the results did not seem to improve much. By default, the cutoff of the deep learning output is set to 0.5. In this study, we found that setting a high cutoff point gave better results for five-layer deep learning. From these results, it seems better to set the cutoff according to the model created.

The advantage of multi-layer deep learning lies in its future expandability. One might aim for higher accuracy by changing the network configuration. If measurement results and diagnoses could be obtained in real-time as training data from machines worldwide, one could perform additional training of the deep learning network and make judgments based on big data. In the conventional method, one has to create an entirely new judgment machine using all the data. In addition, models trained on big data can be adapted to each facility or site by adding data from each site (known as fine tuning).

The inability to see how deep learning systems make their decisions is known as the black box problem. Unwanted results output by deep learning systems can be difficult to fix and can cause ethical problems. We do not think that black box pattern recognition is a disadvantage in this case. It can be compared to visual inspection of a patient. The results of the visual inspection are used simply for reference, just as the deep learning diagnosis in this case. When examining a patient, it is important to visually observe the patient and to assess their condition as good, bad, or in pain. A clinician can instinctively determine a patient's general condition from their facial expressions by recognizing overall patterns without considering any theoretical basis for such judgement. Examination of individual areas of the face, such as the eyes and mouth, provides important findings but that differ from information about general health conditions assessed via visual inspection. A patient's condition can also be inferred from the way they speak. In these cases, the overall pattern is evaluated rather than individual pitch, tempo, intonation, etc. We believe that an approach that recognizes the overall pattern without considering the theoretical basis for the meaning of individual measurements can add value.

The main limitation of this study is that the numbers of healthy participants were relatively small. However, even if the number of participants increases, the reference ranges will likely not be narrowed, and the conclusion that the reference ranges are not clinically useful may not change.

In conclusion, pattern recognition using deep learning is an effective method for evaluation of MostGraph measurement results.

### Supplementary Information

Supplementary Information.

## Data Availability

The best-trained TensorFlow model data is available at https://github.com/sumi-yuki/mostgraph. Individual clinical data cannot be provided because informed consent was obtained on the condition of participants’ anonymity, in that a large number of participant data will be gathered, processed, and presented in a manner such that individual participants cannot be identified. Requests for data from this study other than the data uploaded to GitHub above can be addressed to Yuki Sumi (E-mail: sumi-alg@umin.ac.jp).

## Code availability

All files are available publicly at https://github.com/sumi-yuki/mostgraph. The analyses reported in this study used Python (v.3.9.16), Pandas (v.1.5.3), matplotlib (v.3.7.1)^23^, scikit-learn (v.1.3.0), seaborn (v.0.12.2)^24^, TensorFlow (v.2.10.0), and tensorflowjs (v.3.21.0), running on the Anaconda software on MacOS. Data in Tables 1, 3, 4, 5, 6, and Supplementary Table 1 were calculated using Microsoft Excel for Mac ver.16.61.1. Data in Tables 2 and 3, Fig. 3, and Supplementary Fig. 1 were calculated and displayed using the Python software. For male participants, “YeoJohnson-male.py” available at https://github.com/sumi-yuki/mostgraph/blob/main/YeoJohnson-male.py was used. For female participants, “YeoJohnson-female.py” available at https://github.com/sumi-yuki/mostgraph/blob/main/YeoJohnson-female.py was used. Figures 4 and 5 were displayed using Python. For male participants, “heatmap-male.py” available at https://github.com/sumi-yuki/mostgraph/blob/main/heatmap-male.py was used. For female participants, “heatmap-female.py” available at https://github.com/sumi-yuki/mostgraph/blob/main/heatmap-female.py was used. Figures 2b are displayed using the Python program “deeplearning-mostgraph.py” available at https://github.com/sumi-yuki/mostgraph/blob/main/deeplearning-mostgraph.py. Supplementary Fig. 2 was calculated and displayed using Python programs. For male participants, “YeoJohnson-male-linearregression.py” available at https://github.com/sumi-yuki/mostgraph/blob/main/YeoJohnson-male-linearregression.py was used. For female participants, “YeoJohnson-female-linearregression.py” available at https://github.com/sumi-yuki/mostgraph/blob/main/YeoJohnson-female-linearregression.py was used. Supplementary Fig. 3 was calculated and displayed using the Python program “deeplearning-5layer-smote.py” available at https://github.com/sumi-yuki/mostgraph/blob/main/supplemental/deeplearning-5layer-smote.py. Supplementary Fig. 4 was displayed using the Python program “deeplearning-mono-smote.py” available at https://github.com/sumi-yuki/mostgraph/blob/main/supplemental/deeplearning-mono-smote.py. Supplementary Fig. 5 was displayed using the Python program “logistic-regression-smote.py” available at https://github.com/sumi-yuki/mostgraph/blob/main/supplemental/logistic-regression-smote.py. Supplementary Fig. 6 was displayed using the Python program “deeplearning-5layer-down.py” available at https://github.com/sumi-yuki/mostgraph/blob/main/supplemental/deeplearning-5layer-down.py. The website that performs the judgment of MostGraph measurement results was published at https://sumi-yuki.github.io/mostgraph/ and https://plaza.umin.ac.jp/~pulm/mostgraph/index.html

## References

[1] King GG (2020). Technical standards for respiratory oscillometry. Eur. Respir. J..

[2] Oostveen E (2003). The forced oscillation technique in clinical practice: methodology, recommendations and future developments. Eur. Respir. J..

[3] Shirai T, Kurosawa H (2016). Clinical application of the forced oscillation technique. Intern. Med..

[4] Bhattarai P (2020). Clinical application of forced oscillation technique (FOT) in early detection of airway changes in smokers. J. Clin. Med..

[5] Milne S (2019). Respiratory system reactance reflects communicating lung volume in chronic obstructive pulmonary disease. J. Appl. Physiol..

[6] Abe Y (2016). Reference values of MostGraph measures for middle-aged and elderly Japanese individuals who participated in annual health checkups. Respir. Investig..

[7] Global Initiative for Asthma, Global Strategy for Asthma Management and Prevention (Updated 2023), preprint Available from: https://ginasthma.org/wp-content/uploads/2023/07/GINA-2023-Full-report-23_07_06-WMS.pdf (2023).

[8] Sumi Y (2007). Airway remodeling in asthma. Allergol. Int..

[9] Matsunaga K (2011). Exhaled nitric oxide cutoff values for asthma diagnosis according to rhinitis and smoking status in Japanese subjects. Allergol. Int..

[10] Matsunaga K (2010). Reference ranges for exhaled nitric oxide fraction in healthy Japanese adult population. Allergol. Int..

[11] Yeo I-K (2000). A new family of power transformations to improve normality or symmetry. Biometrika.

[12] scikit-learn sklearn.preprocessing.PowerTransformer. Available from: https://scikit-learn.org/stable/modules/generated/sklearn.preprocessing.PowerTransformer.html

[13] scikit-learn sklearn.preprocessing.PowerTransformer. Available from: https://scikit-learn.org/stable/modules/generated/sklearn.preprocessing.PowerTransformer.html#sklearn.preprocessing.PowerTransformer.inverse_transform

[14] Nakamura Y (2020). Japanese guidelines for adult asthma 2020. Allergol. Int..

[15] Ichinose M (2017). Japanese guidelines for adult asthma 2017. Allergol. Int..

[16] Ohta K (2014). Japanese guideline for adult asthma 2014. Allergol. Int..

[17] Ohta K (2011). Japanese guideline for adult asthma. Allergol. Int..

[18] Amaral JLM (2017). High-accuracy detection of airway obstruction in asthma using machine learning algorithms and forced oscillation measurements. Comput. Methods Programs Biomed..

[19] Amaral JL (2020). Differential diagnosis of asthma and restrictive respiratory diseases by combining forced oscillation measurements, machine learning and neuro-fuzzy classifiers. Med. Biol. Eng. Comput..

[20] Amaral JL (2015). Machine learning algorithms and forced oscillation measurements to categorise the airway obstruction severity in chronic obstructive pulmonary disease. Comput. Methods Programs Biomed..

[21] Andrade DSM (2021). Machine learning associated with respiratory oscillometry: a computer-aided diagnosis system for the detection of respiratory abnormalities in systemic sclerosis. Biomed. Eng. Online..

[22] Marinho CL (2017). Respiratory resistance and reactance in adults with sickle cell anemia: Correlation with functional exercise capacity and diagnostic use. PLoS One.

[23] Hunter JD (2007). Matplotlib: A 2D graphics environment. Comput. Sci. Eng..

[24] Waskom ML (2021). seaborn: statistical data visualization. J. Open Source Softw..

