# Supplementary material for: Pattern recognition of forced oscillation technique measurement results using deep learning can identify asthmatic patients more accurately than setting reference ranges
Source: Sci Rep. 2023 Dec 7;13:21608. doi: 10.1038/s41598-023-48042-3 (PMC10703832; doi:10.1038/s41598-023-48042-3)
Supplement: Supplementary file 1 — Supplementary Information. [file 41598_2023_48042_MOESM1_ESM.pdf]

Pattern recognition of forced oscillation technique measurement results using deep learning can identify asthmatic patients more accurately than setting reference range

SUPPLEMENTARY MATERIAL

Supplementary Figure 1

Histogram of MostGraph measurement values in healthy controls and histograms of the Yeo-Johnson power transformed values. These are supplementary histograms of Figure 3 showing all MostGraph measurement items.

Red dotted line indicates the mean and pink square indicates mean ± 2 × Standard Deviation of the transformed value.

These values and figures were calculated and displayed in Python programs. For male participants, "YeoJohnson-male.py" available at https://github.com/sumi-yuki/mostgraph/blob/main/YeoJohnson-male.py was used. For female participants, "YeoJohnson-female.py" available at https://github.com/sumi-yuki/mostgraph/blob/main/YeoJohnson-female.py was used.

Male: R5 Histogram

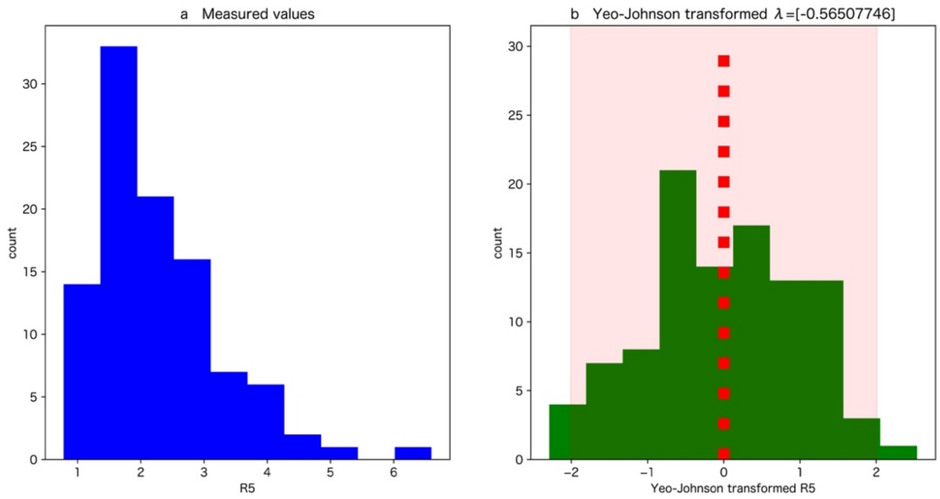

Male: R5in Histogram

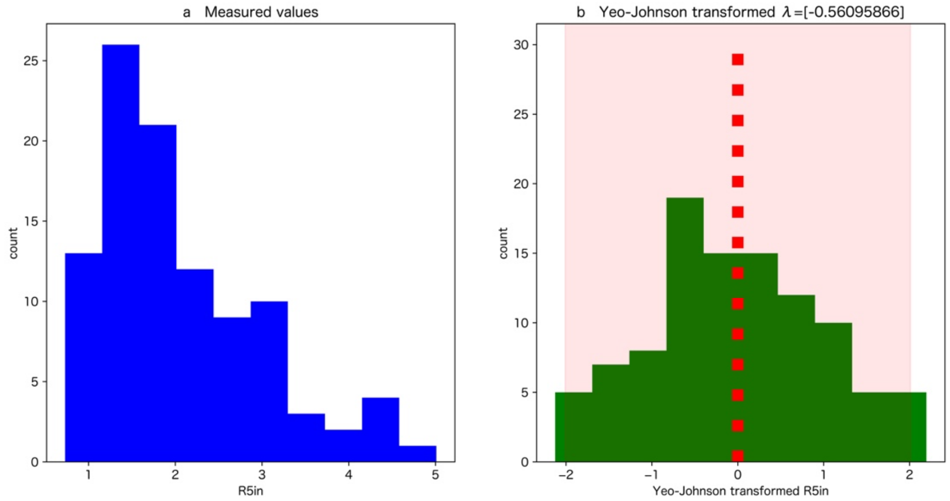

Male: R5ex Histogram

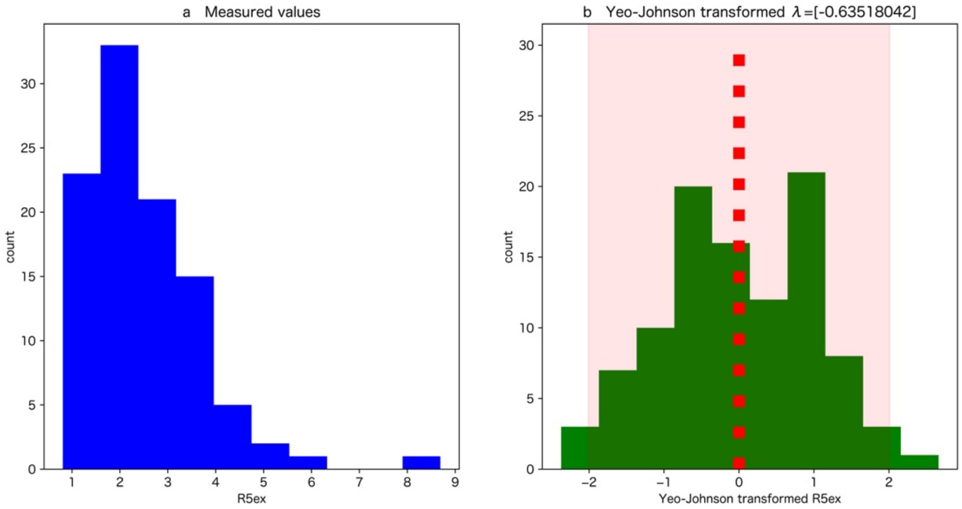

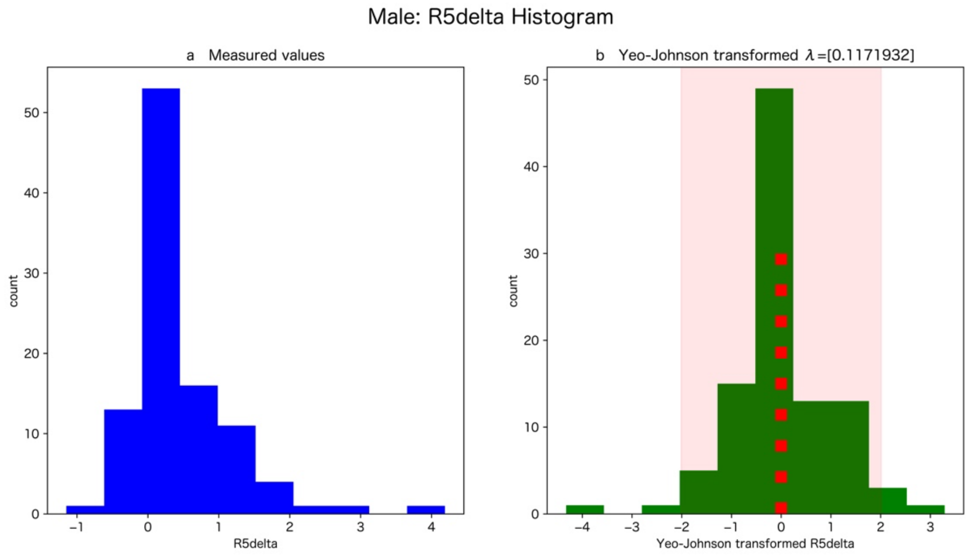
Male: R5delta Histogram
a Measured values
count
R5delta
b Yeo-Johnson transformed λ =[0.1171932]
count
Yeo-Johnson transformed R5delta

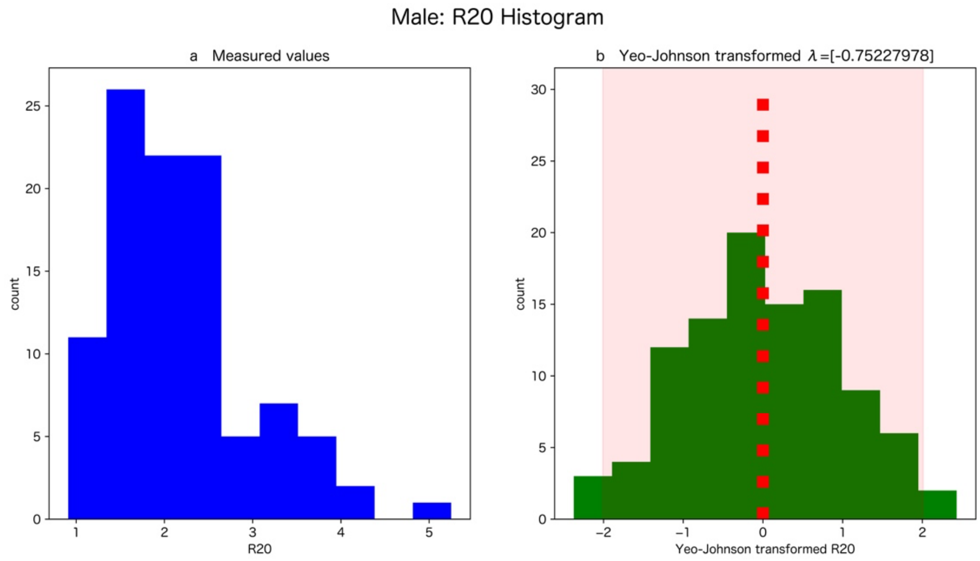
Male: R20 Histogram
a Measured values
count
R20
b Yeo-Johnson transformed λ =[-0.75227978]
count
Yeo-Johnson transformed R20

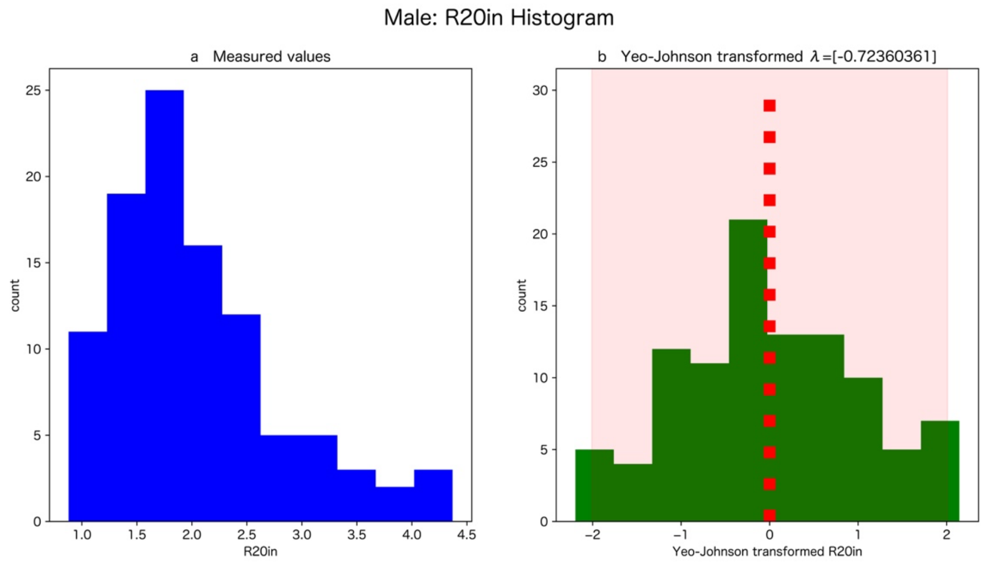
Male: R20in Histogram
a Measured values
count
R20in
b Yeo-Johnson transformed λ =[-0.72360361]
count
Yeo-Johnson transformed R20in

Male: R20ex Histogram

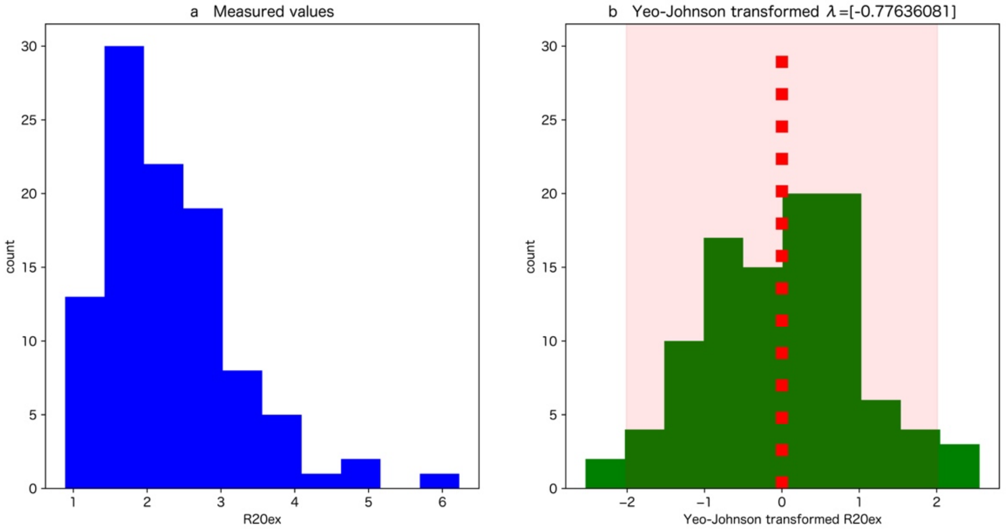

Male: R20delta Histogram

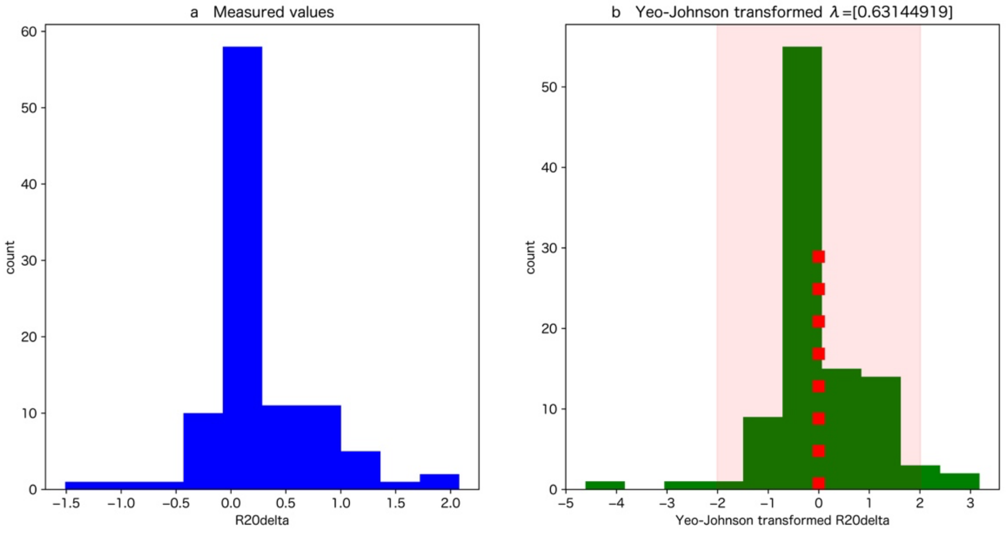

Male: R5-R20 Histogram

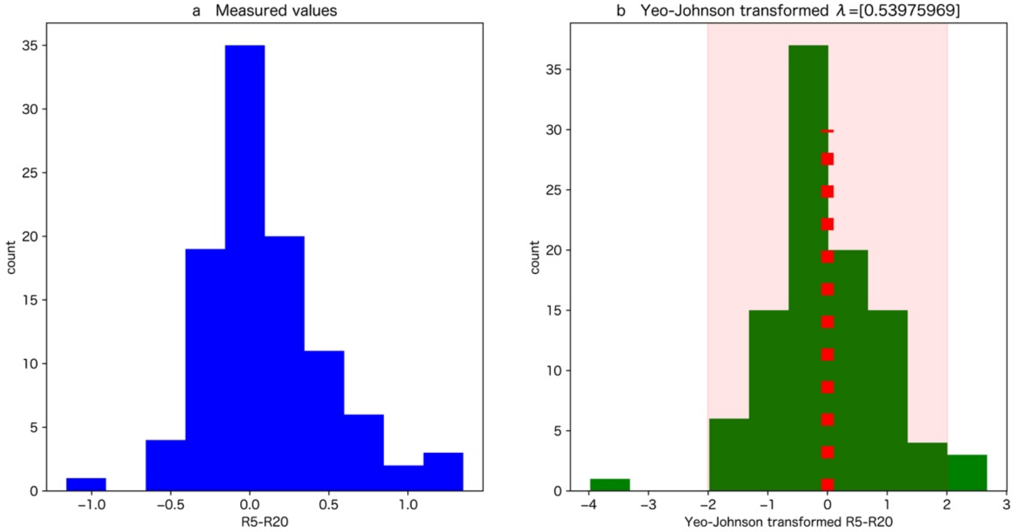

Male: R5-R20in Histogram

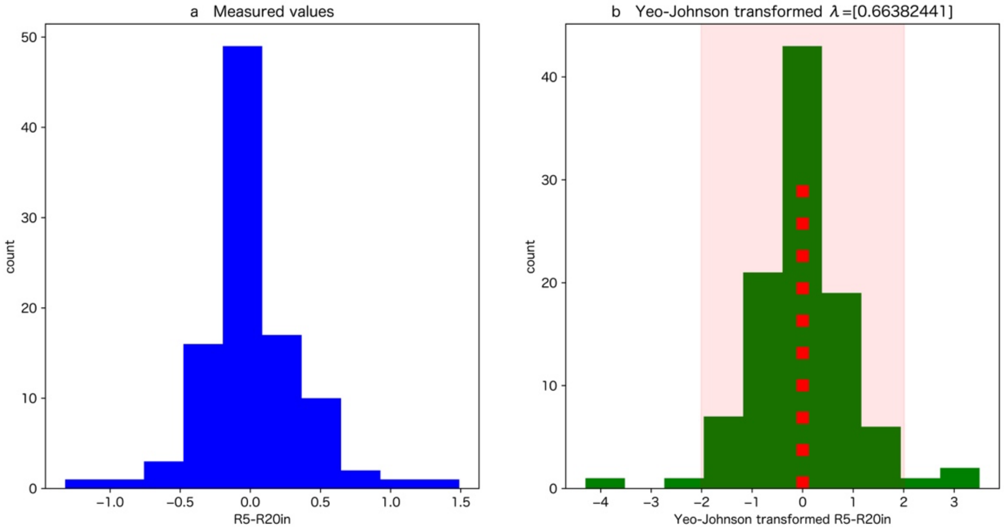

Male: R5-R20ex Histogram

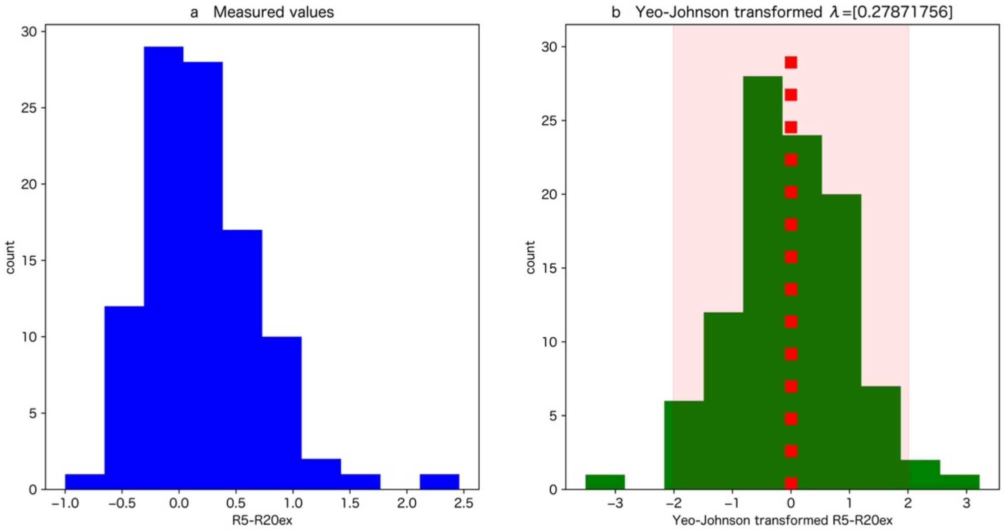

Male: R5-R20delta Histogram

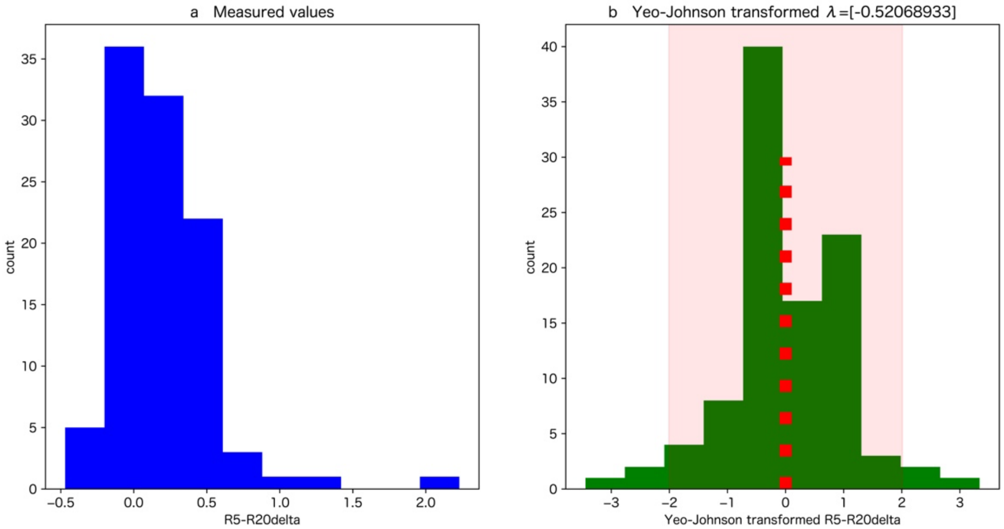

Male: X5 Histogram

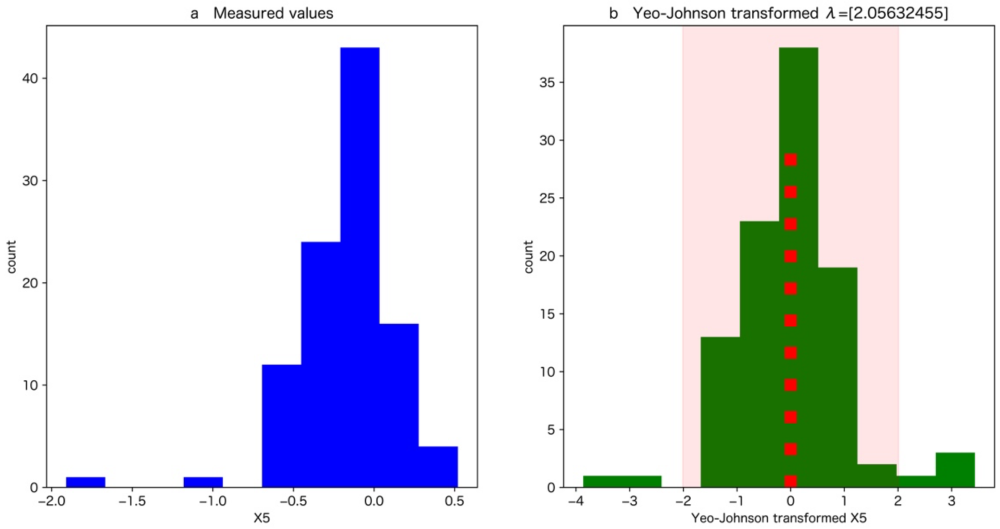

Male: X5in Histogram

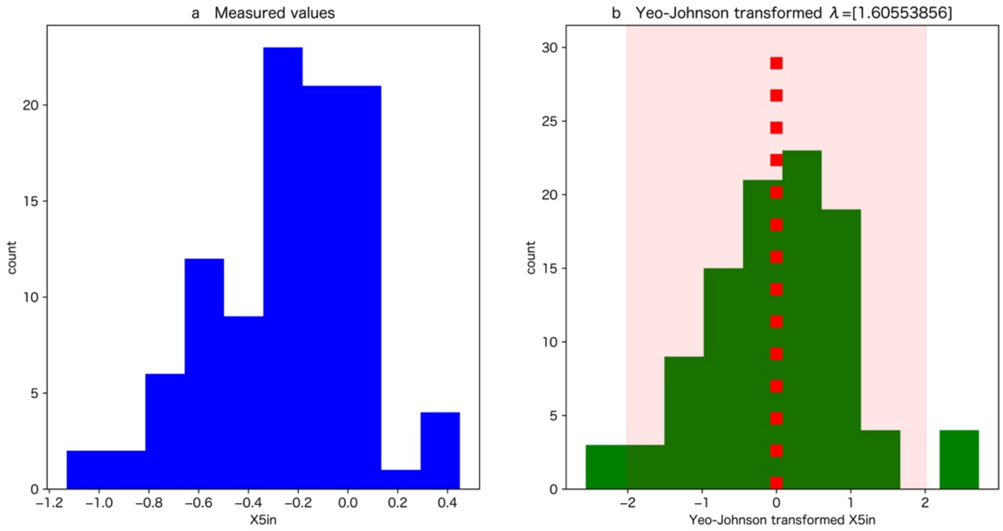

Male: X5ex Histogram

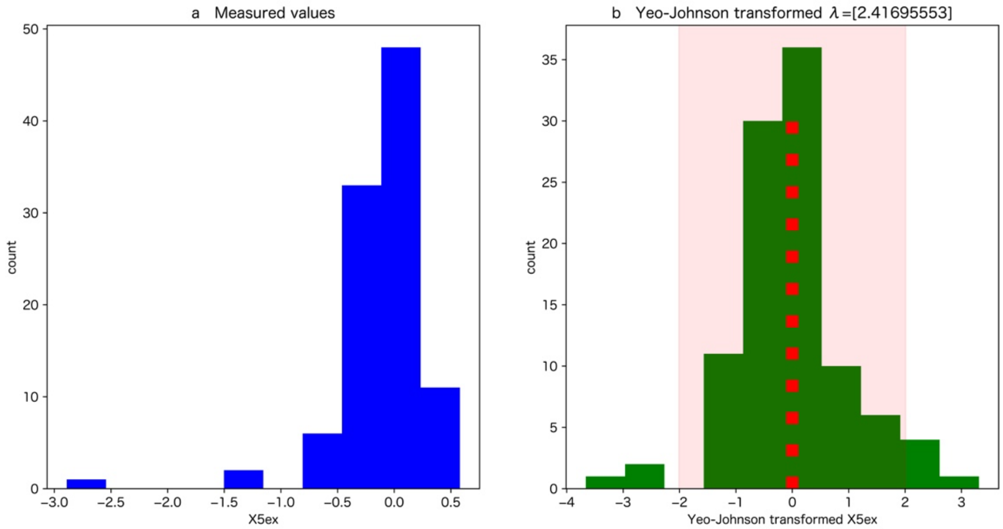

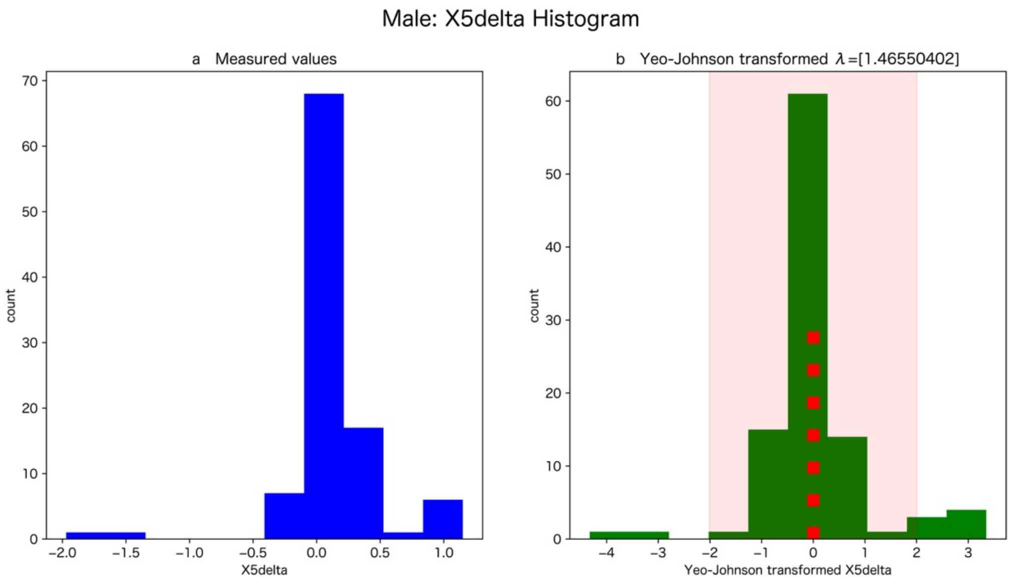
Male: X5delta Histogram
a Measured values
count
X5delta
b Yeo-Johnson transformed λ=[1.46550402]
count
Yeo-Johnson transformed X5delta

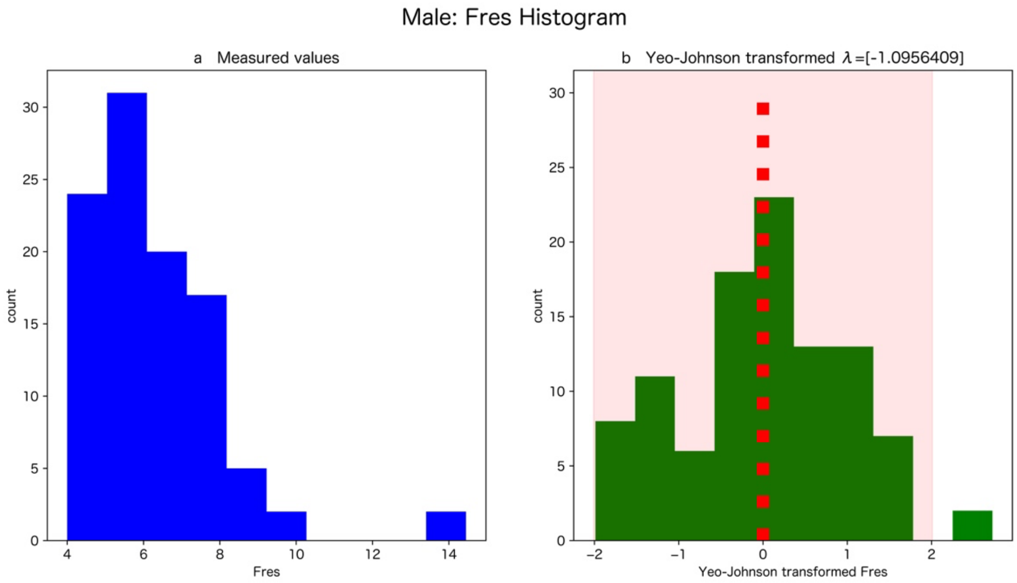
Male: Fres Histogram
a Measured values
count
Fres
b Yeo-Johnson transformed λ=[-1.0956409]
count
Yeo-Johnson transformed Fres

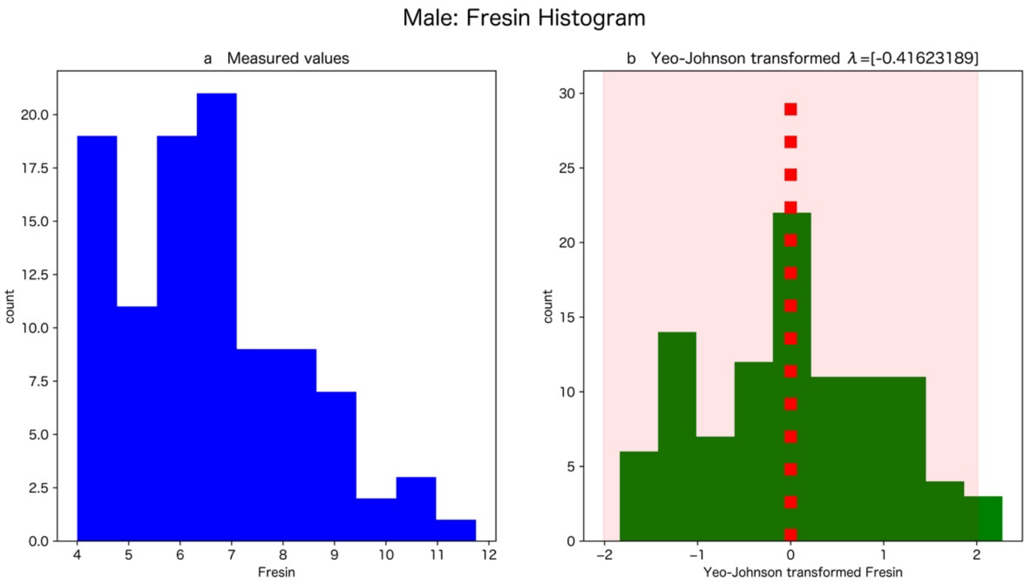
Male: Fresin Histogram
a Measured values
count
Fresin
b Yeo-Johnson transformed λ=[-0.41623189]
count
Yeo-Johnson transformed Fresin

Male: Fresex Histogram

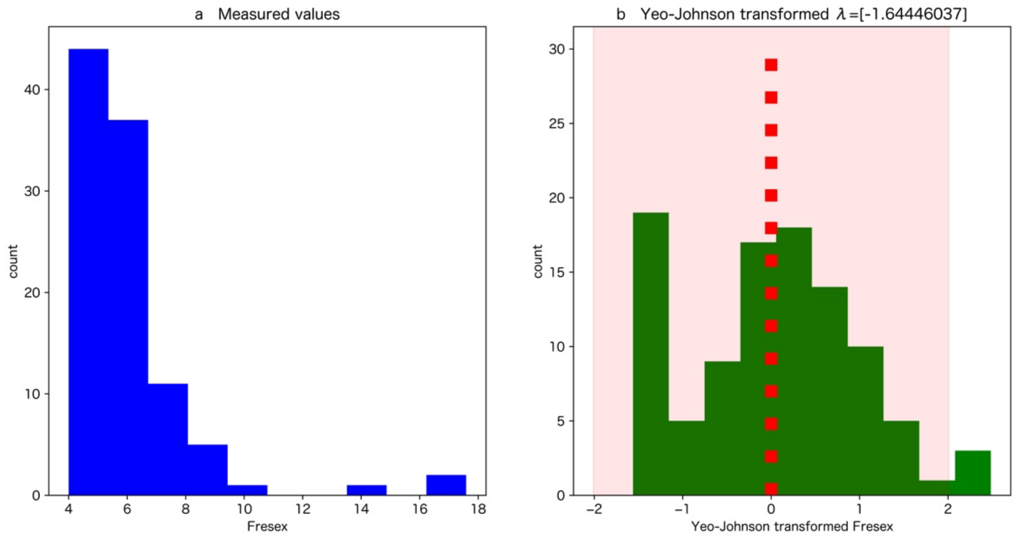

Male: Fresdelta Histogram

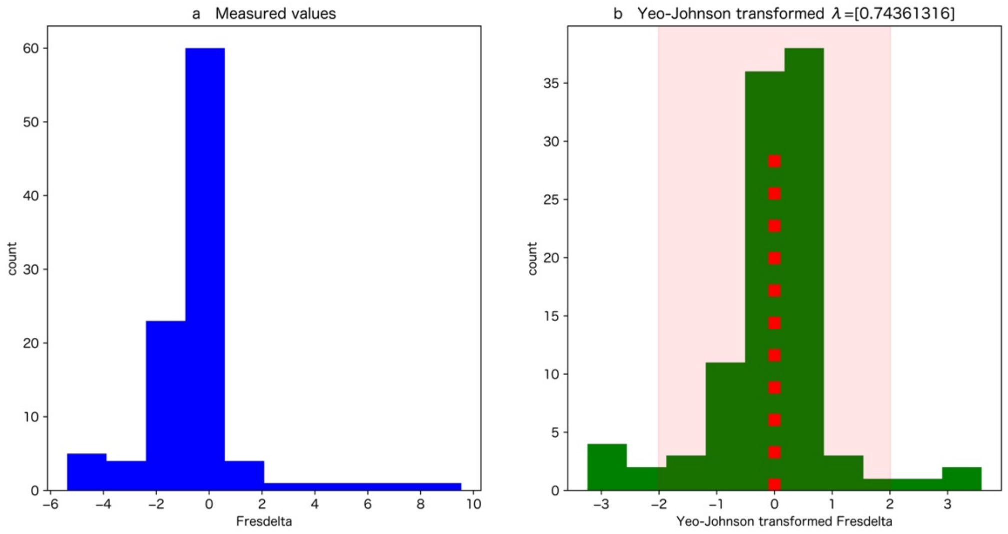

Male: ALX Histogram

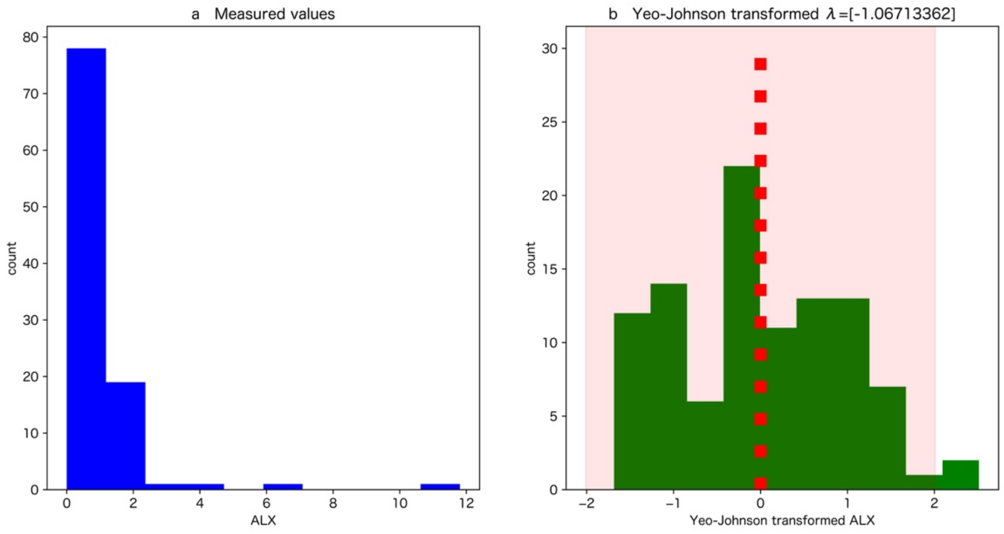

Male: ALXin Histogram

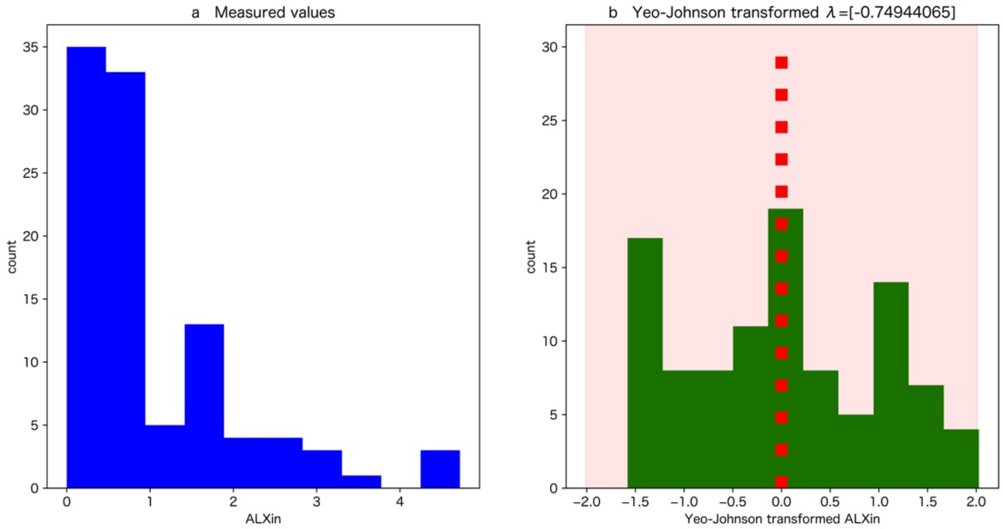

Male: ALXex Histogram

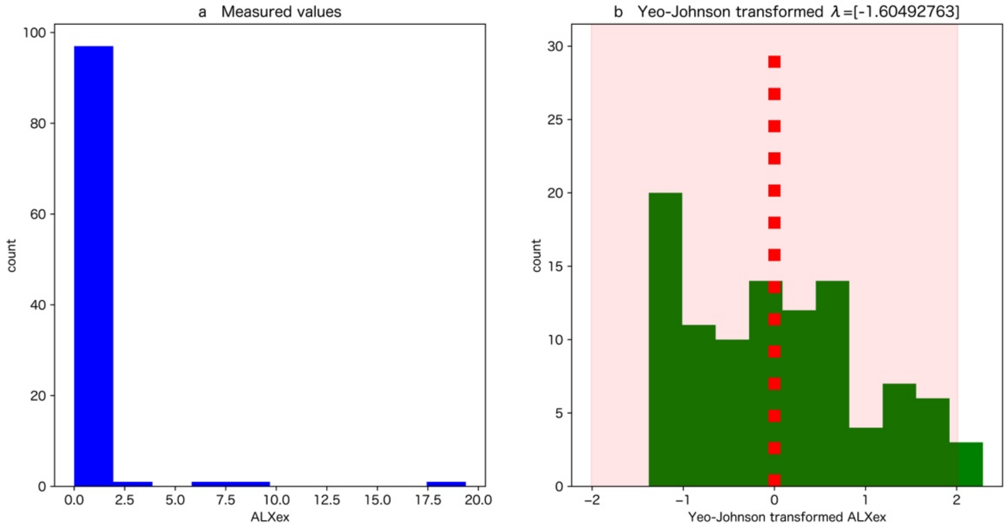

Male: ALXdelta Histogram

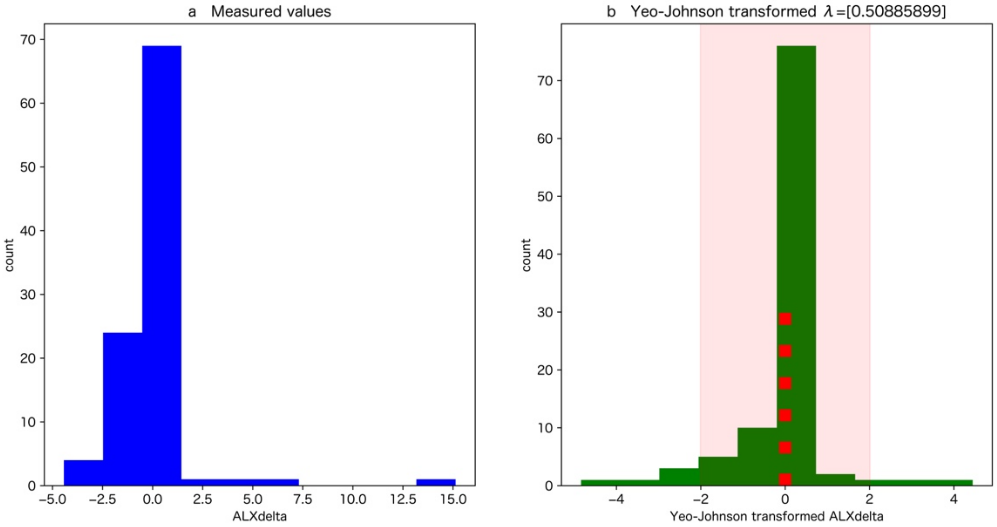

Female: R5 Histogram

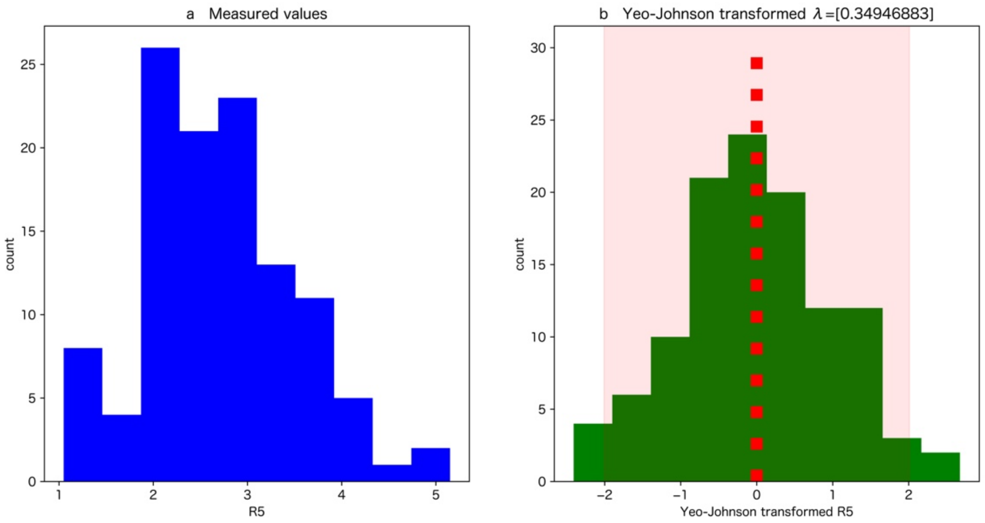

Female: R5in Histogram

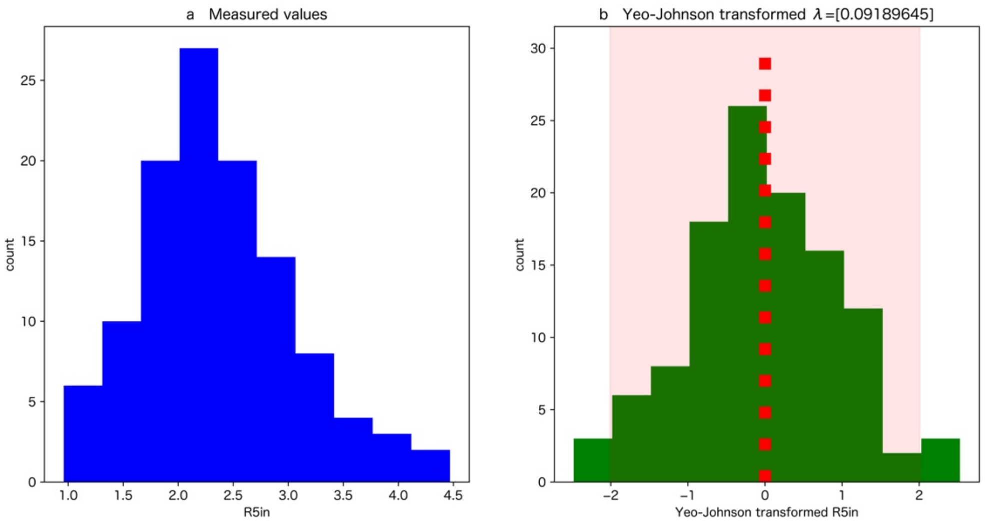

Female: R5ex Histogram

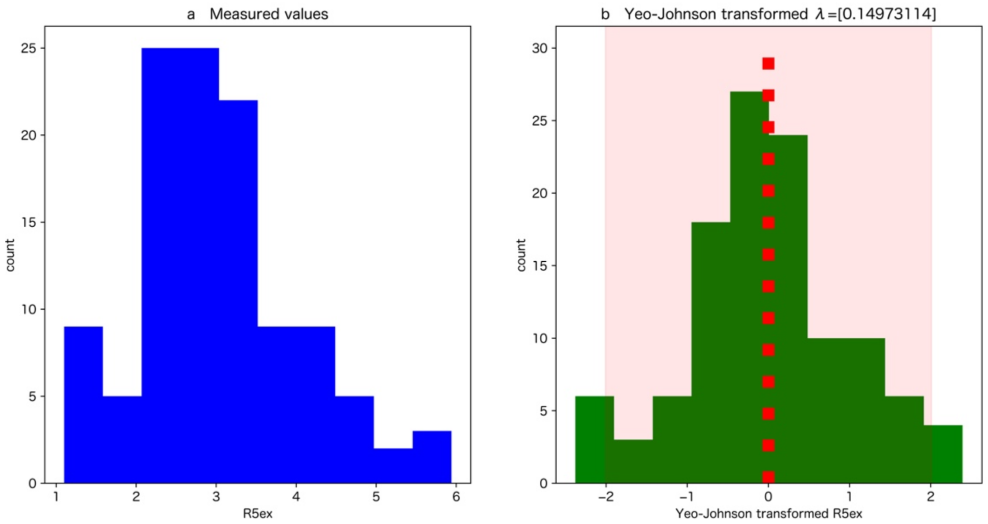

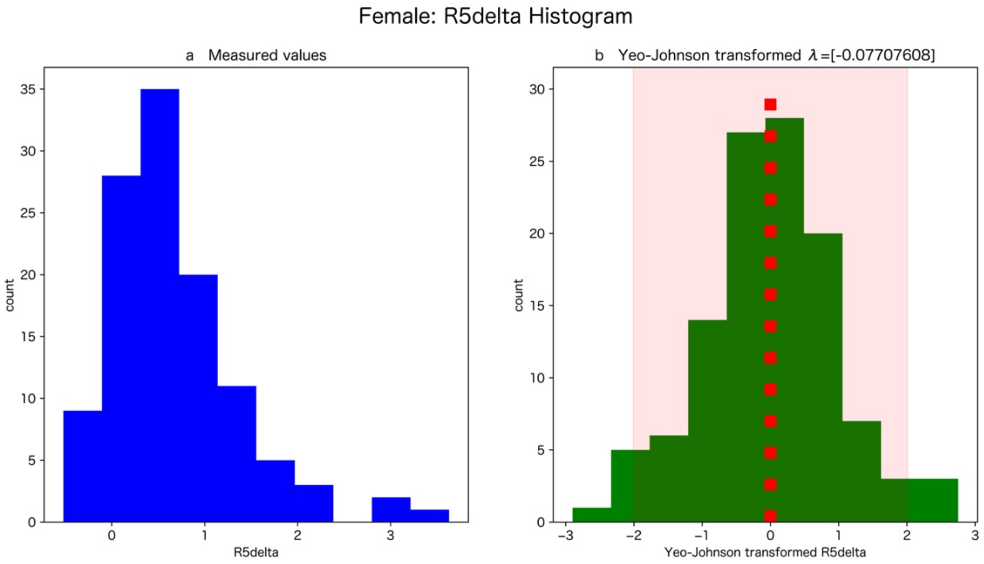
Female: R5delta Histogram
a Measured values
count
R5delta
b Yeo-Johnson transformed λ=[-0.07707608]
count
Yeo-Johnson transformed R5delta

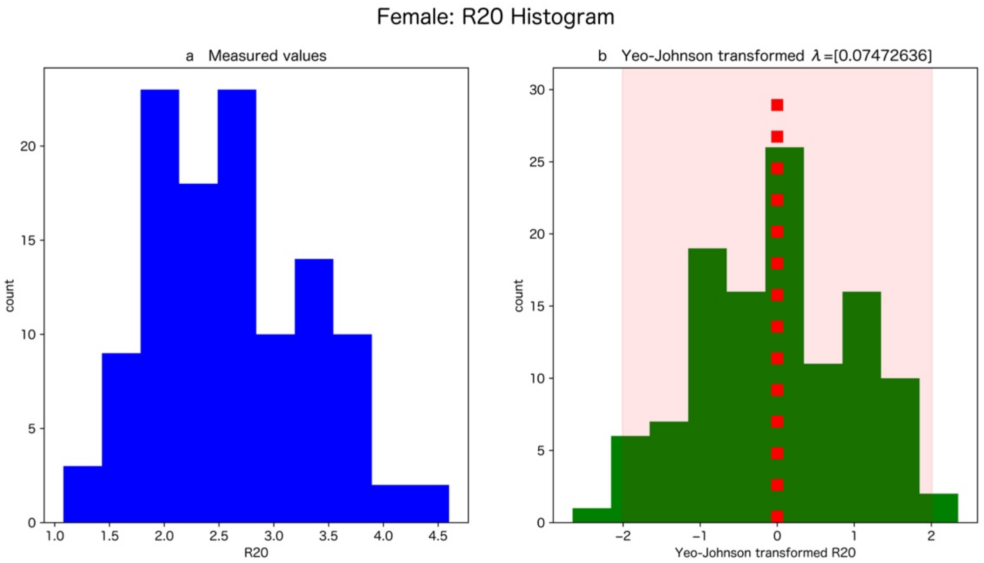
Female: R20 Histogram
a Measured values
count
R20
b Yeo-Johnson transformed λ=[0.07472636]
count
Yeo-Johnson transformed R20

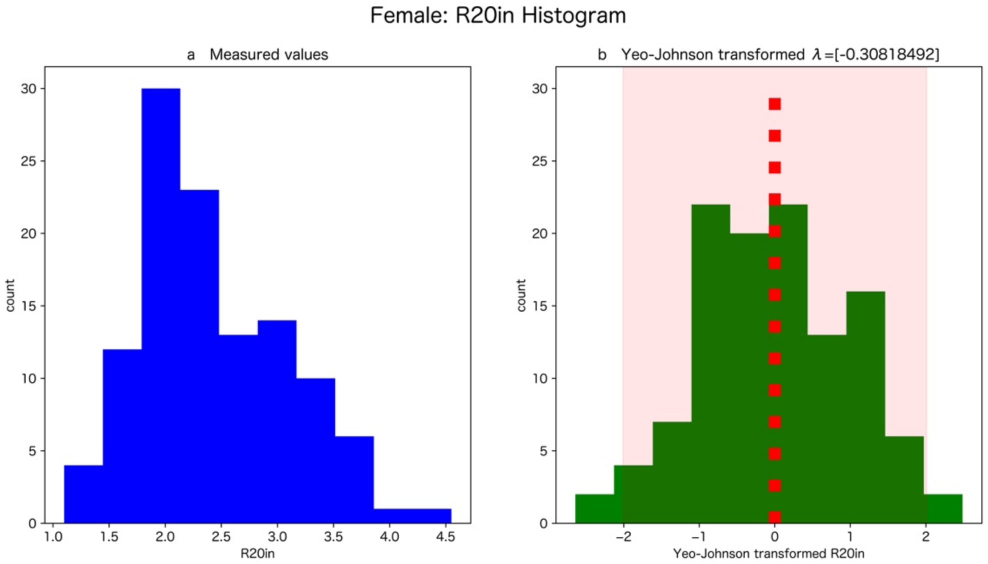
Female: R20in Histogram
a Measured values
count
R20in
b Yeo-Johnson transformed λ=[-0.30818492]
count
Yeo-Johnson transformed R20in

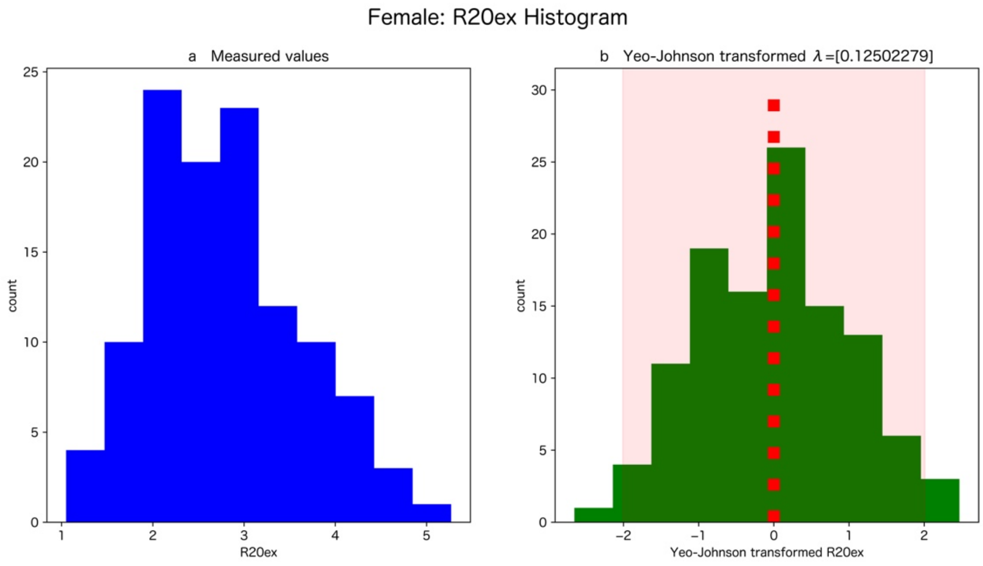
Female: R20ex Histogram
a Measured values
b Yeo-Johnson transformed λ=[0.12502279]
count
R20ex
Yeo-Johnson transformed R20ex

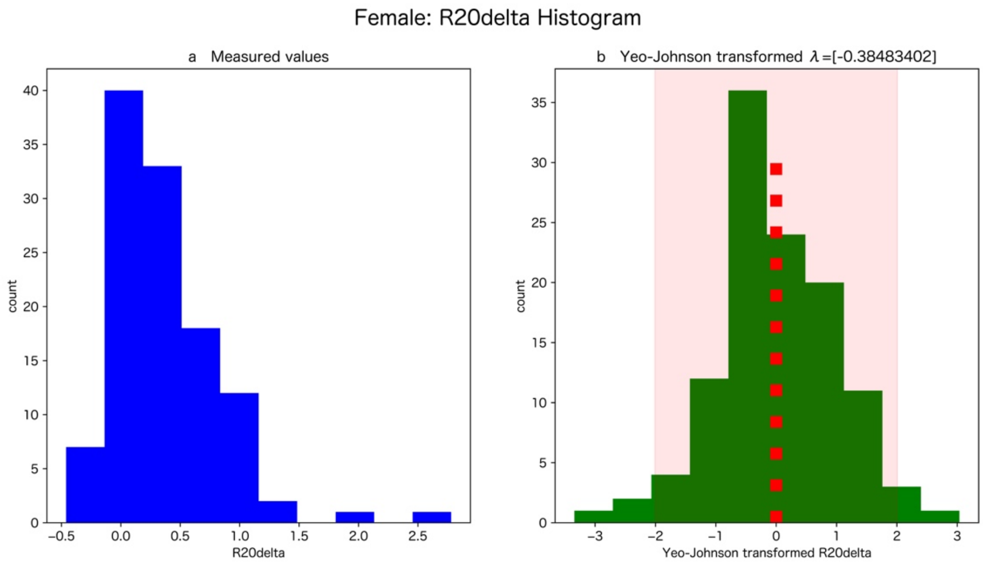
Female: R20delta Histogram
a Measured values
b Yeo-Johnson transformed λ=[-0.38483402]
count
R20delta
Yeo-Johnson transformed R20delta

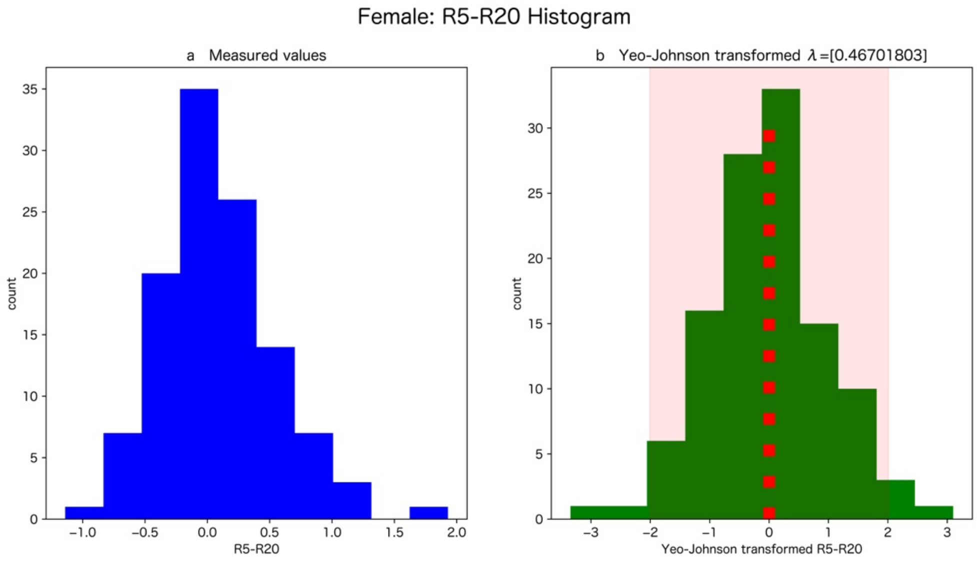
Female: R5-R20 Histogram
a Measured values
b Yeo-Johnson transformed λ=[0.46701803]
count
R5-R20
Yeo-Johnson transformed R5-R20

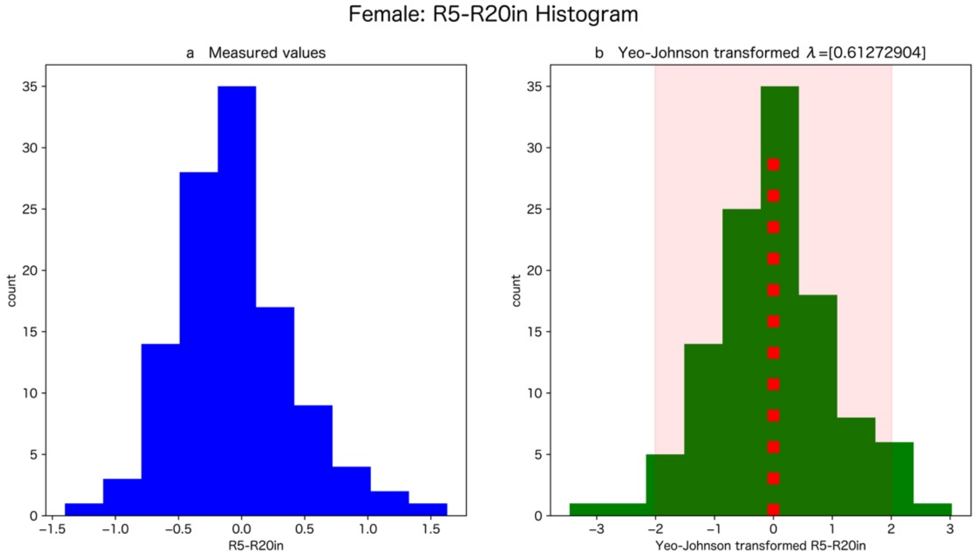
Female: R5-R20in Histogram
a Measured values
b Yeo-Johnson transformed λ=[0.61272904]
count
R5-R20in
Yeo-Johnson transformed R5-R20in

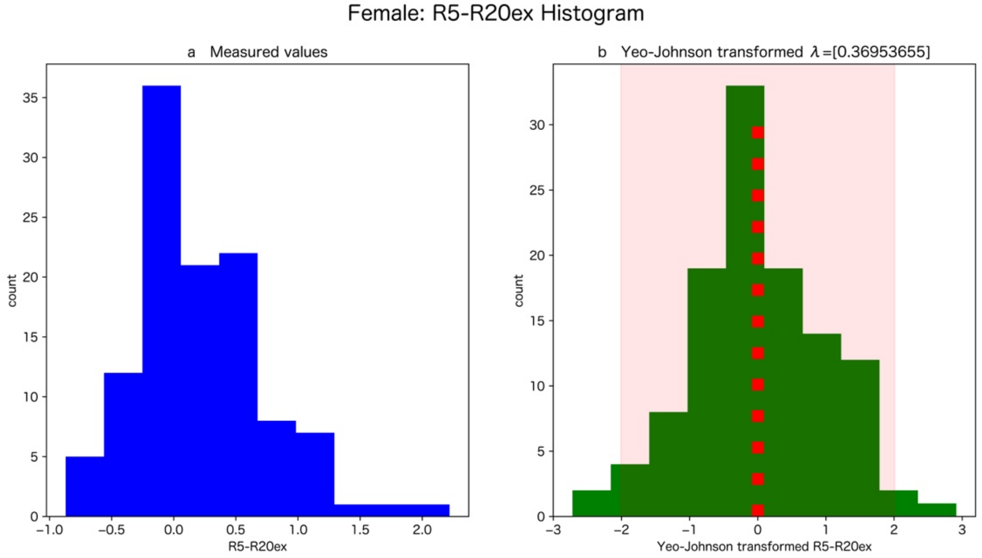
Female: R5-R20ex Histogram
a Measured values
b Yeo-Johnson transformed λ=[0.36953655]
count
R5-R20ex
Yeo-Johnson transformed R5-R20ex

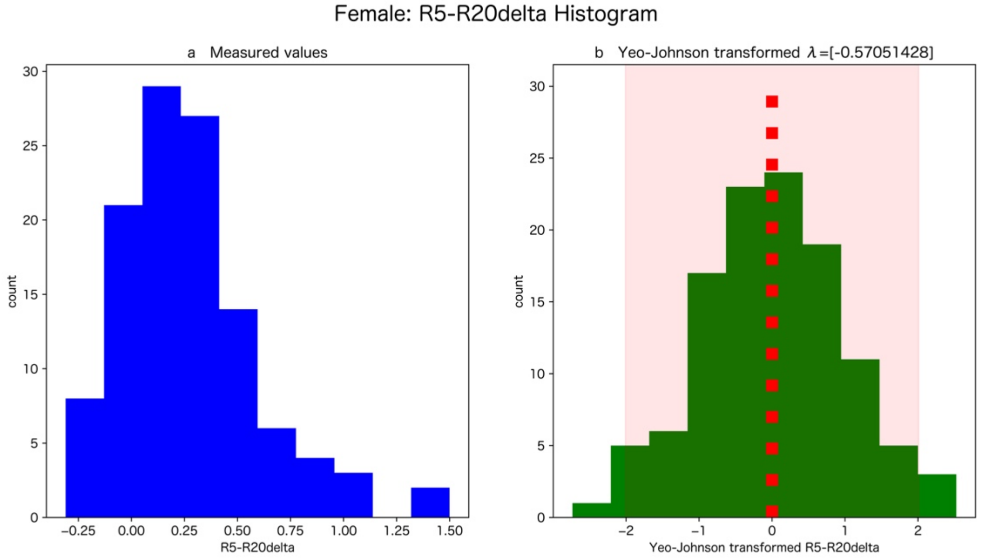
Female: R5-R20delta Histogram
a Measured values
b Yeo-Johnson transformed λ=[-0.57051428]
count
R5-R20delta
Yeo-Johnson transformed R5-R20delta

Female: X5 Histogram

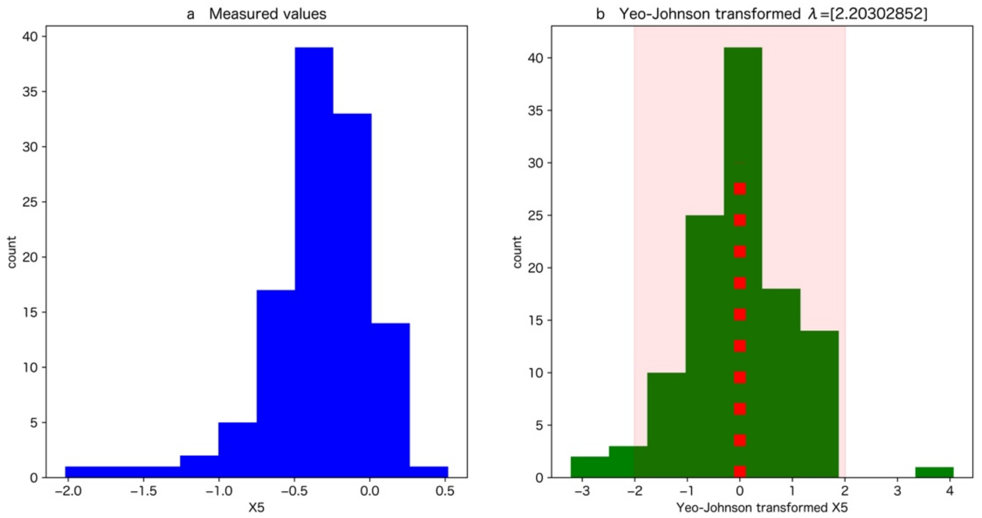

Female: X5in Histogram

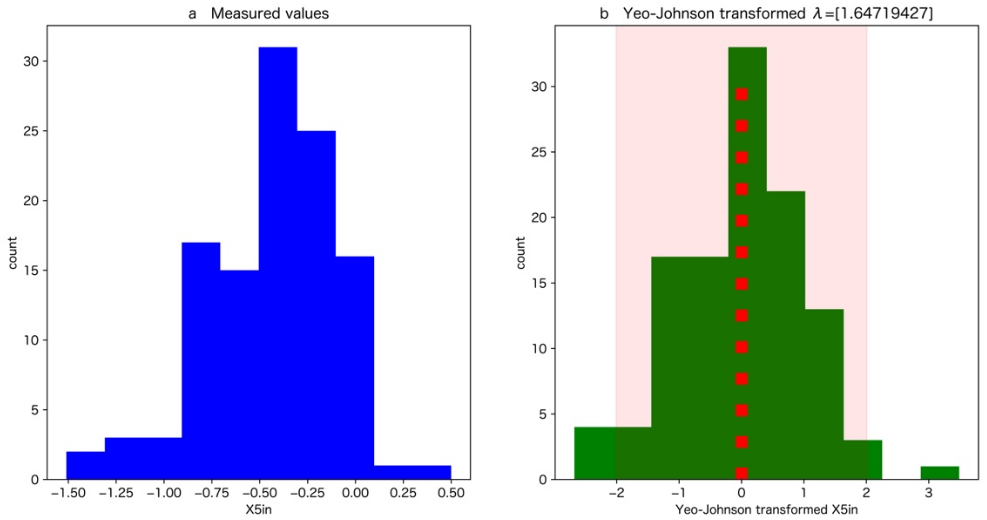

Female: X5ex Histogram

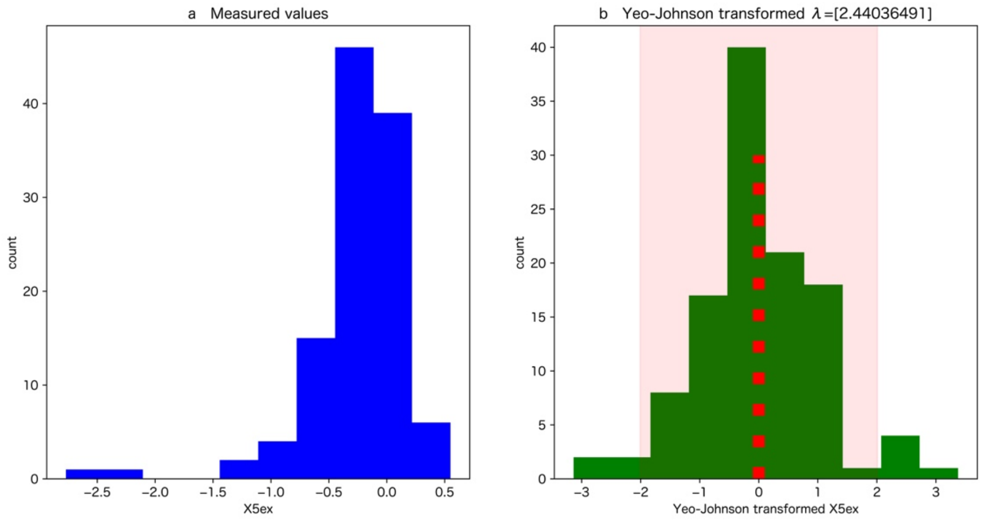

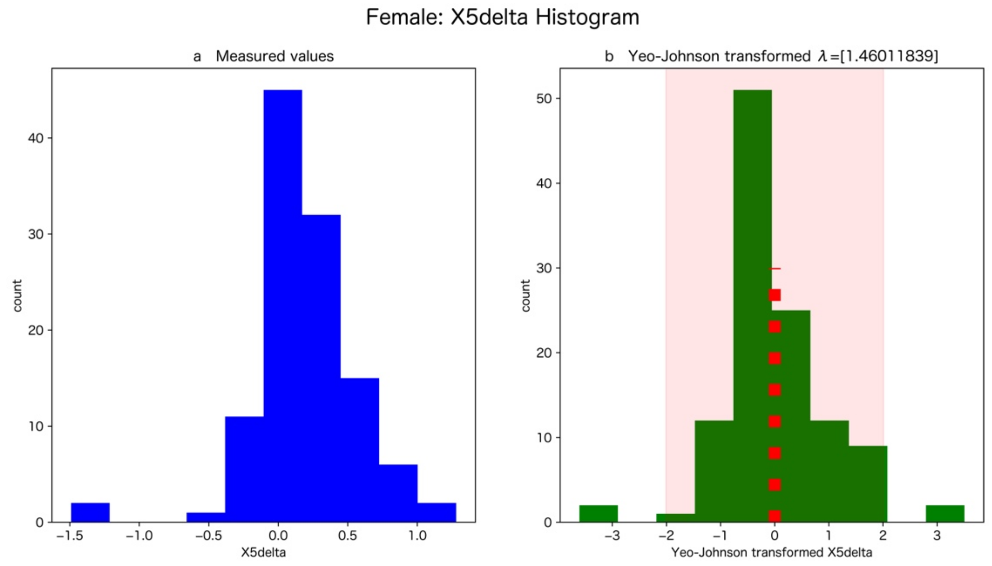
Female: X5delta Histogram
a Measured values
count
X5delta
b Yeo-Johnson transformed λ=[1.46011839]
Yeo-Johnson transformed X5delta

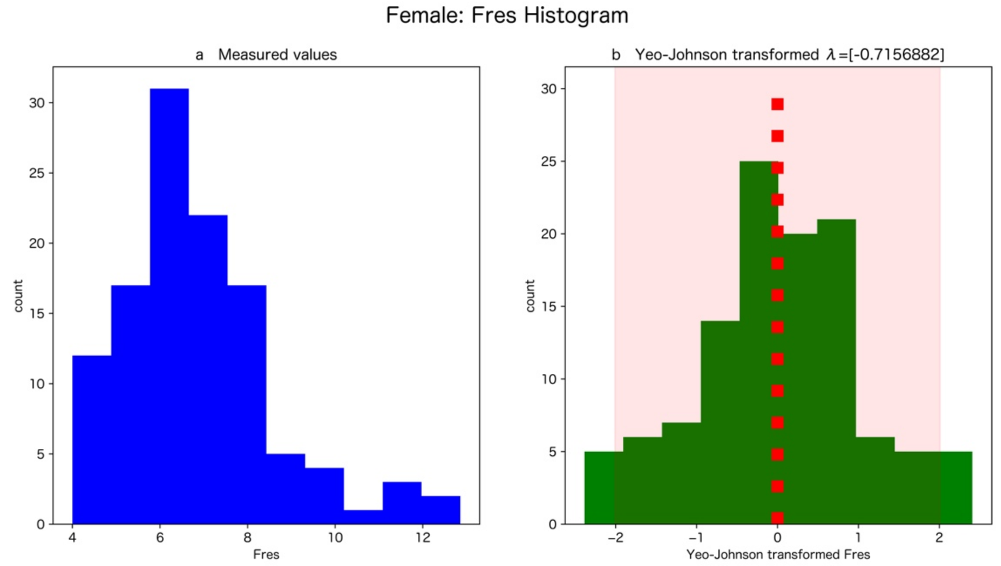
Female: Fres Histogram
a Measured values
count
Fres
b Yeo-Johnson transformed λ=[-0.7156882]
Yeo-Johnson transformed Fres

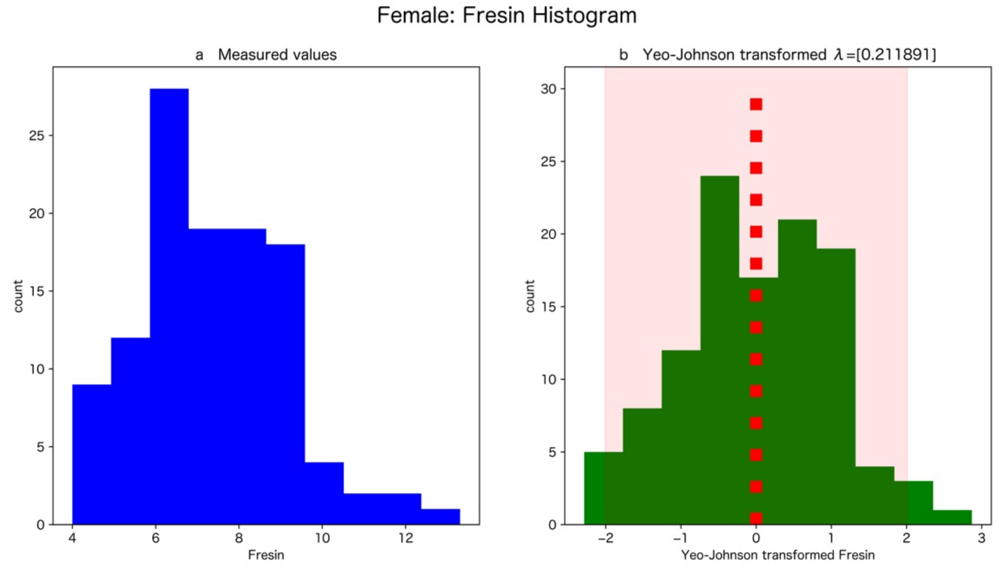
Female: Fresin Histogram
a Measured values
count
Fresin
b Yeo-Johnson transformed λ=[0.211891]
Yeo-Johnson transformed Fresin

Female: Fresex Histogram

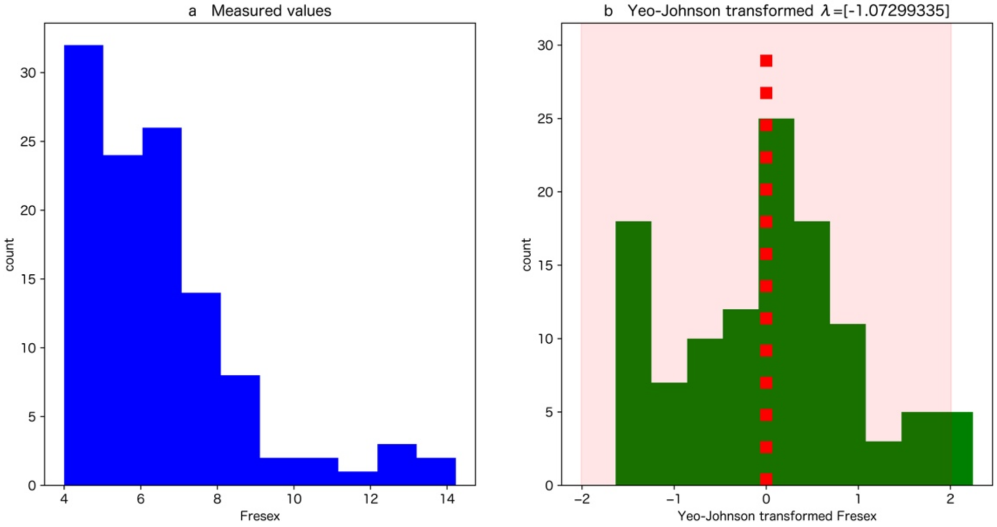

Female: Fresdelta Histogram

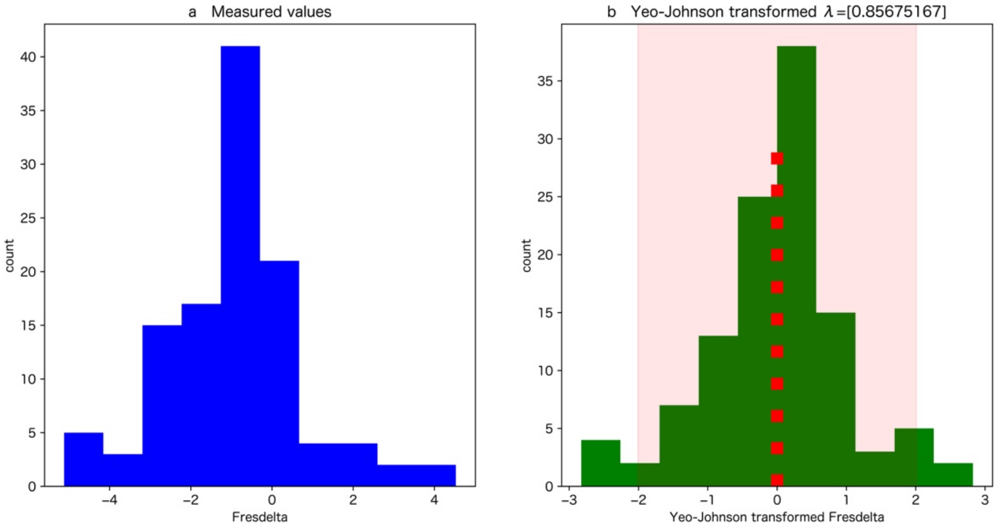

Female: ALX Histogram

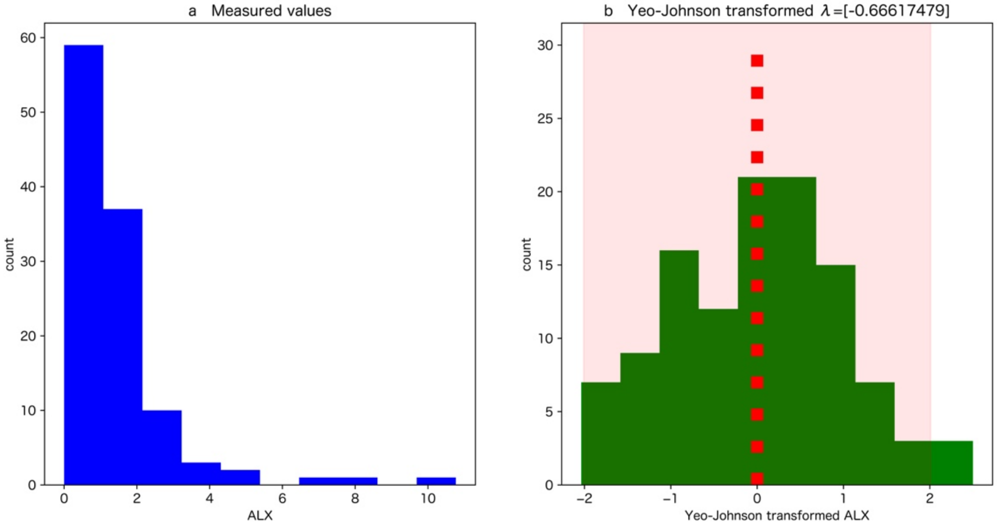

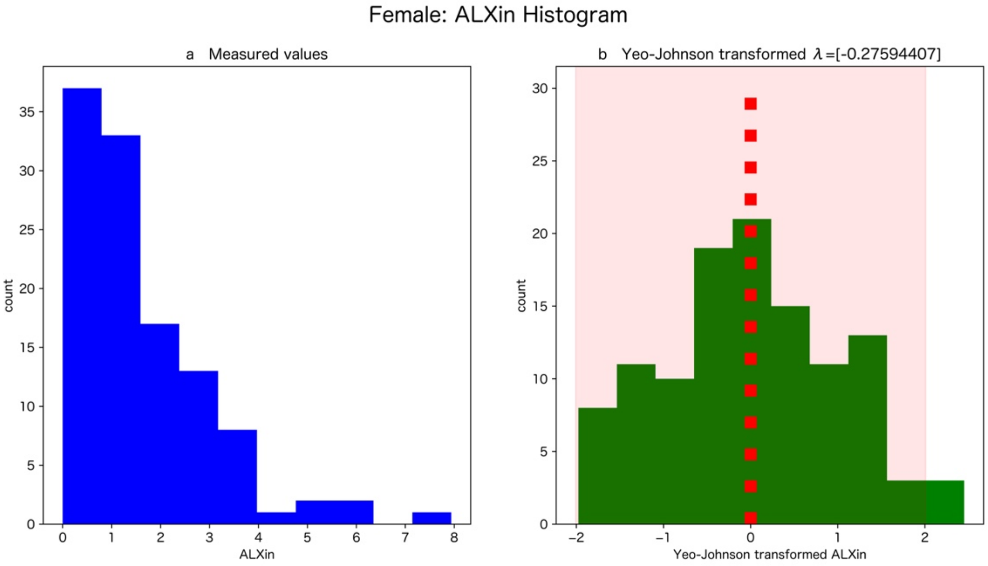
Female: ALXin Histogram
a Measured values
count
ALXin
b Yeo-Johnson transformed λ=[-0.27594407]
count
Yeo-Johnson transformed ALXin

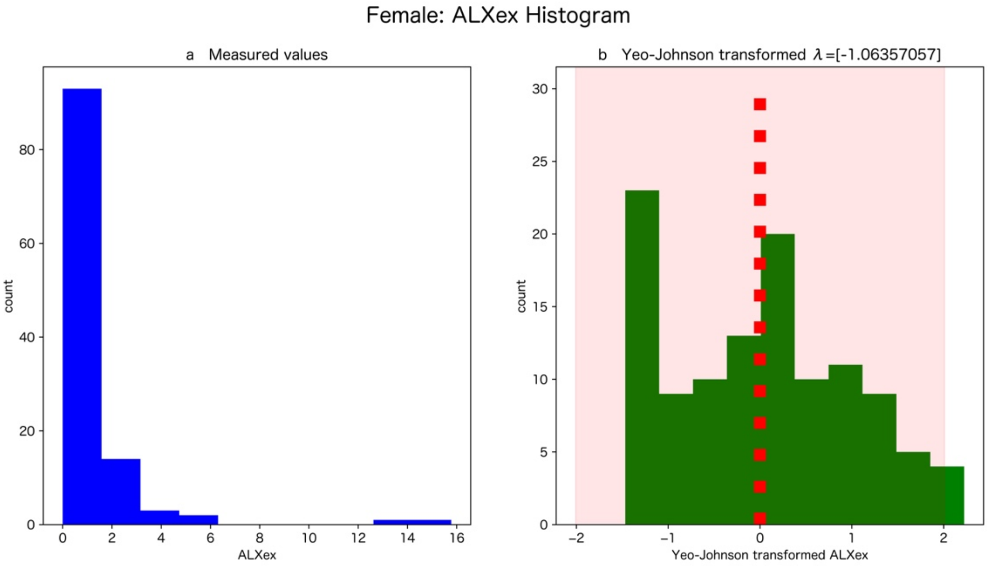
Female: ALXex Histogram
a Measured values
count
ALXex
b Yeo-Johnson transformed λ=[-1.06357057]
count
Yeo-Johnson transformed ALXex

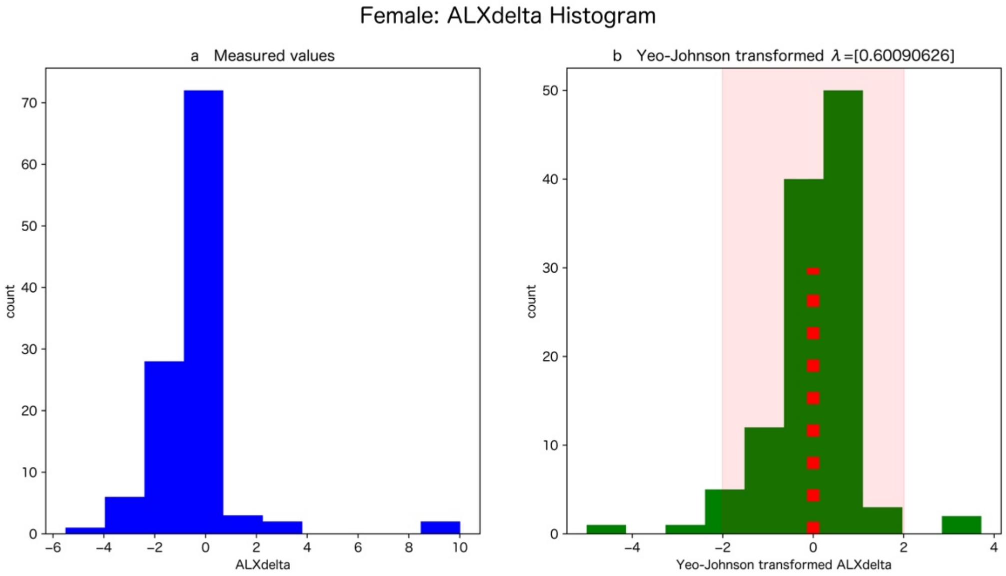
Female: ALXdelta Histogram
a Measured values
count
ALXdelta
b Yeo-Johnson transformed λ=[0.60090626]
count
Yeo-Johnson transformed ALXdelta

Supplementary Figure 2

Comparison of reference ranges established by mean ± 2 × Standard Deviation (SD) of the Yeo-Johnson transformed values (pink square) to the multiple linear regression analysis using age, height, and weight as explanatory variables (green or blue line).

For multiple linear regression analysis, the differences between the Yeo-Johnson transformed measured values and predicted values were used to calculate the SD. The predicted values ± 2 × SD were set for the reference ranges of the power transformed values.

The X-axis represents the measured values and the Y-axis represents the predicted values. The black dots indicate the predicted value of each healthy control calculated by sex, age, height, and weight, showing the relationship with the measured values. Ideally, these should be represented on the black lines. The green bars denote the reference ranges of the Yeo-Johnson transformed values, and the blue bars denote the reference ranges of the MostGraph measured values. Most of these lines intersect the black line, so we know that our predictions are correct; however, we were unable to narrow the ranges of the pink square.

According to these results, setting references by multiple regression analysis did not show better predictions than using mean ± 2 × SD of the Yeo-Johnson transformed values.

These values were calculated and displayed in Python programs. For male participants, "YeoJohnson-male-linearregression.py" available at https://github.com/sumi-yuki/mostgraph/blob/main/YeoJohnson-male-linearregression.py was used. For female participants, "YeoJohnson-female-linearregression.py" available at https://github.com/sumi-yuki/mostgraph/blob/main/YeoJohnson-female-linearregression.py was used.

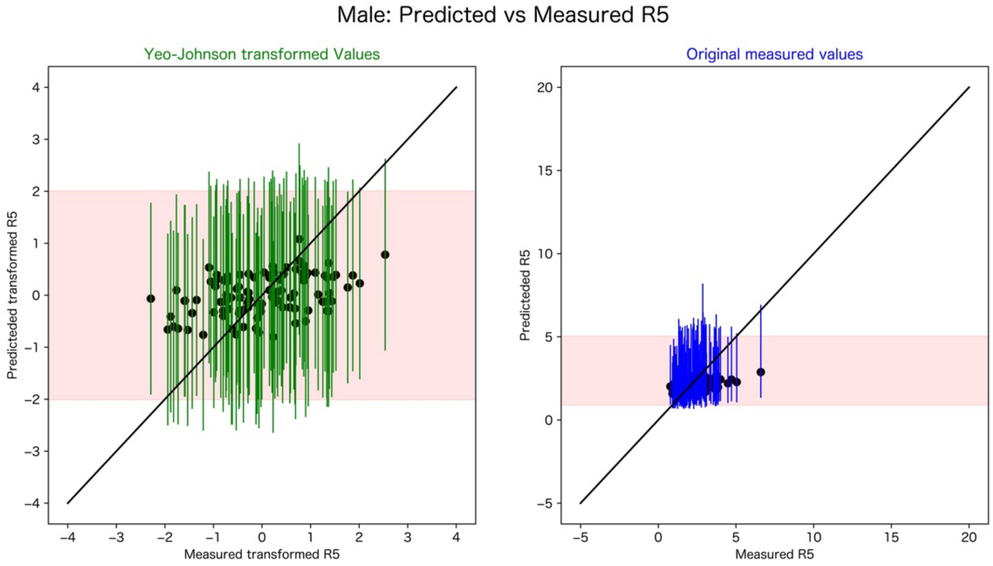
Male: Predicted vs Measured R5
Yeo-Johnson transformed Values
Original measured values
Predicteded transformed R5
Measured transformed R5
Predicteded R5
Measured R5

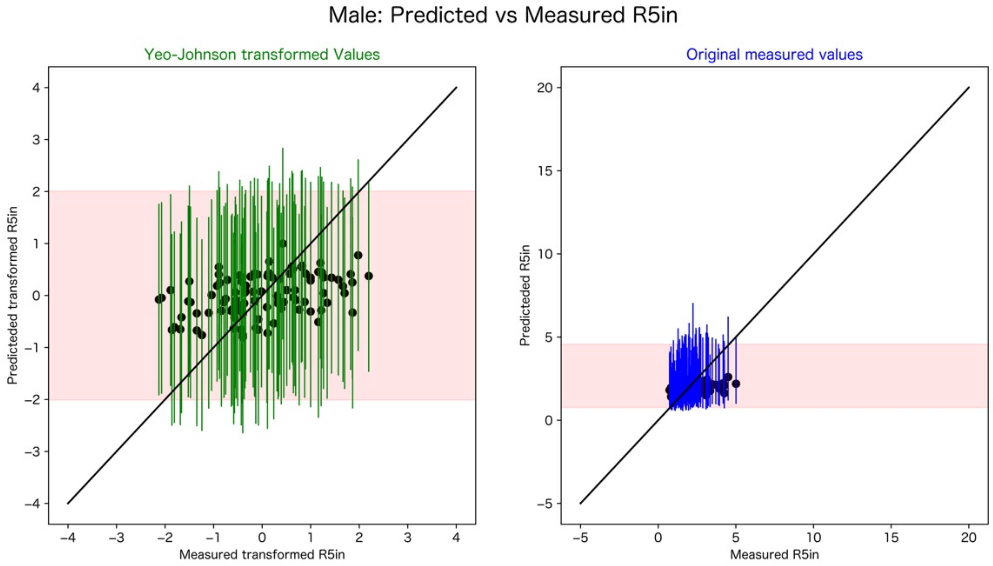
Male: Predicted vs Measured R5in
Yeo-Johnson transformed Values
Original measured values
Predicteded transformed R5in
Measured transformed R5in
Predicteded R5in
Measured R5in

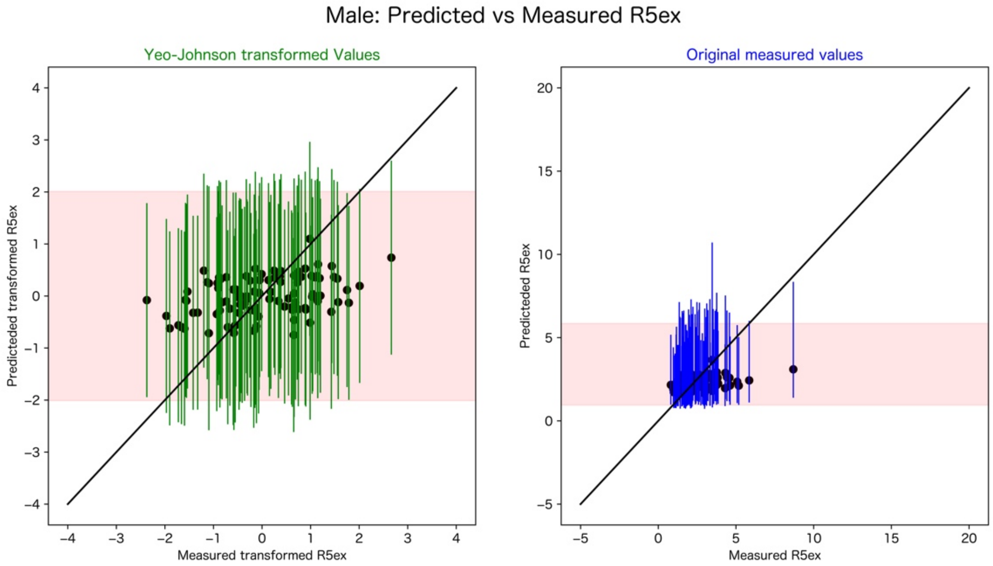
Male: Predicted vs Measured R5ex
Yeo-Johnson transformed Values
Original measured values
Predicteded transformed R5ex
Measured transformed R5ex
Predicteded R5ex
Measured R5ex

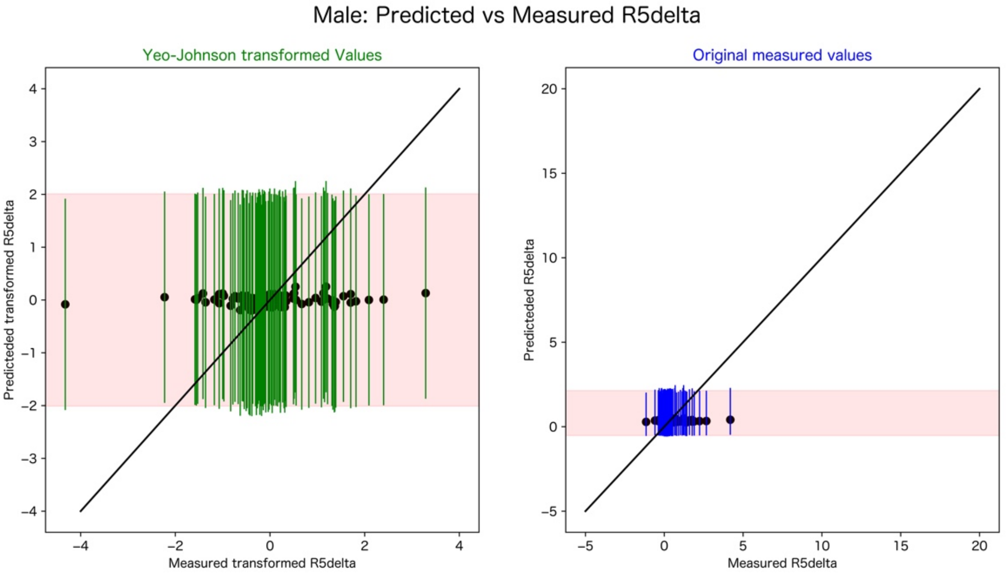
Male: Predicted vs Measured R5delta
Yeo-Johnson transformed Values
Predicteded transformed R5delta
Measured transformed R5delta
Original measured values
Predicteded R5delta
Measured R5delta

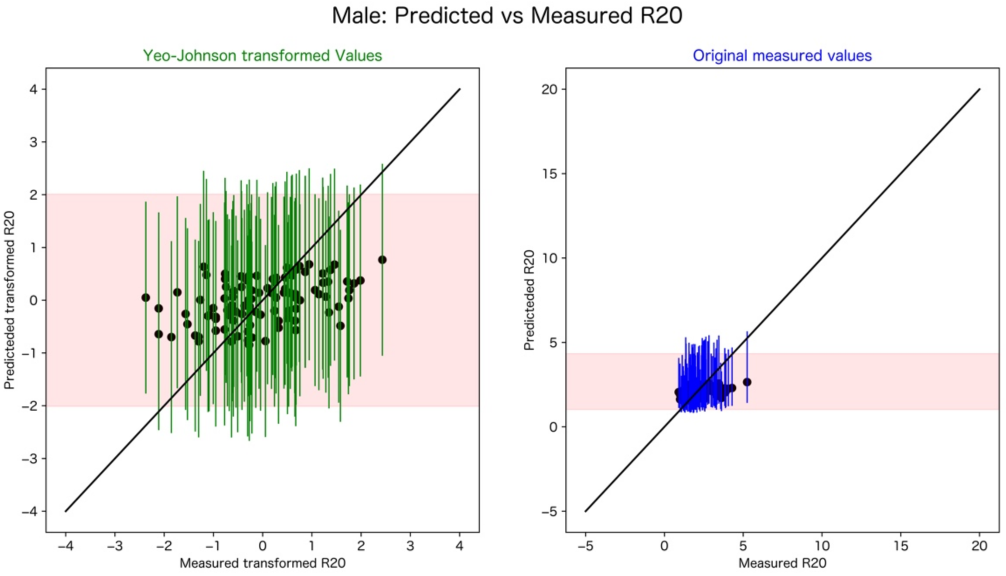
Male: Predicted vs Measured R20
Yeo-Johnson transformed Values
Predicteded transformed R20
Measured transformed R20
Original measured values
Predicteded R20
Measured R20

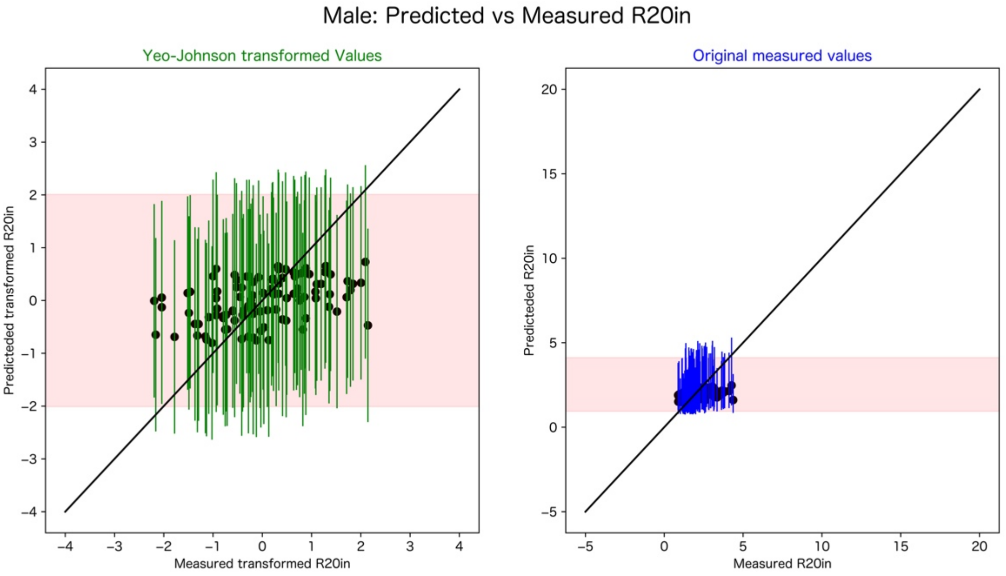
Male: Predicted vs Measured R20in
Yeo-Johnson transformed Values
Predicteded transformed R20in
Measured transformed R20in
Original measured values
Predicteded R20in
Measured R20in

Male: Predicted vs Measured R20ex

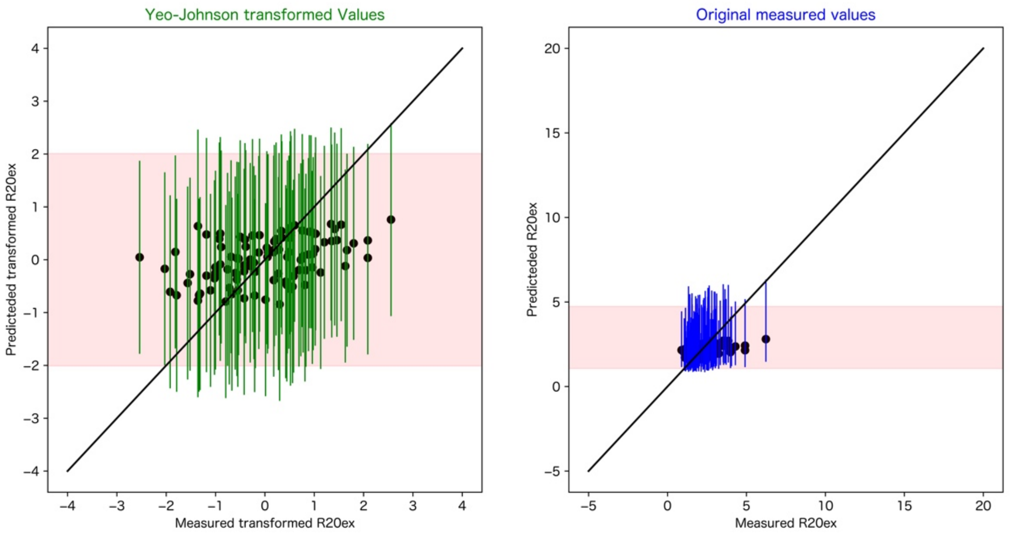

Male: Predicted vs Measured R20delta

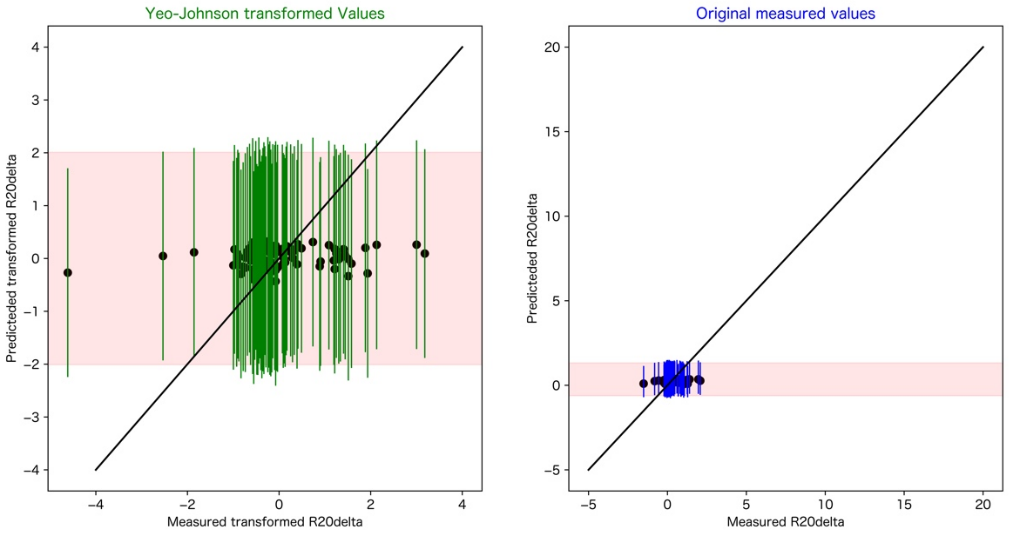

Male: Predicted vs Measured R5-R20

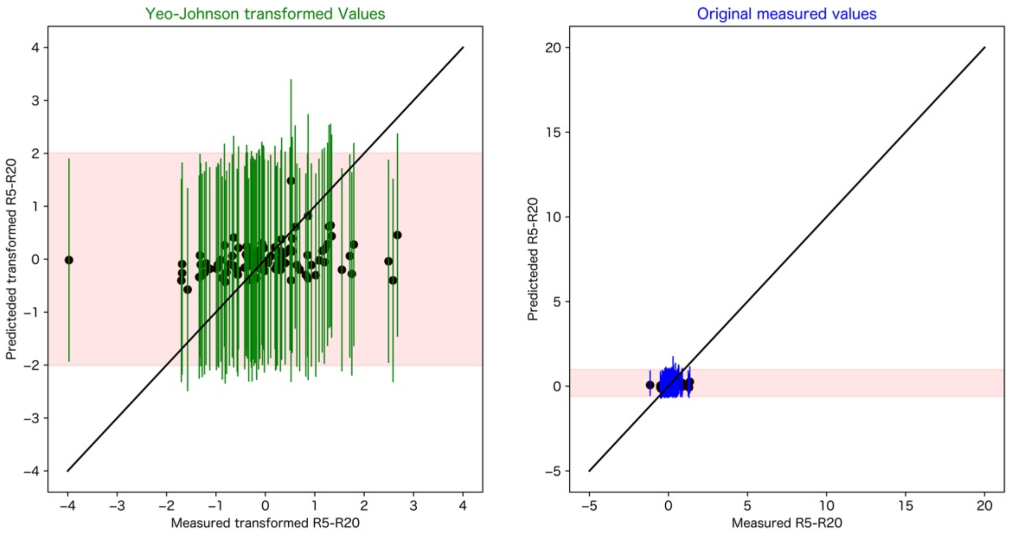

Male: Predicted vs Measured R5-R20in

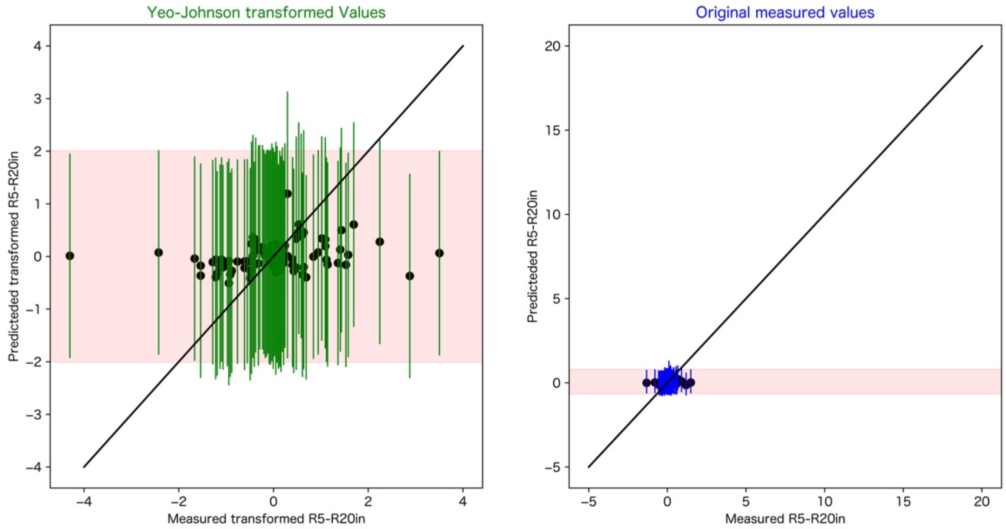

Male: Predicted vs Measured R5-R20ex

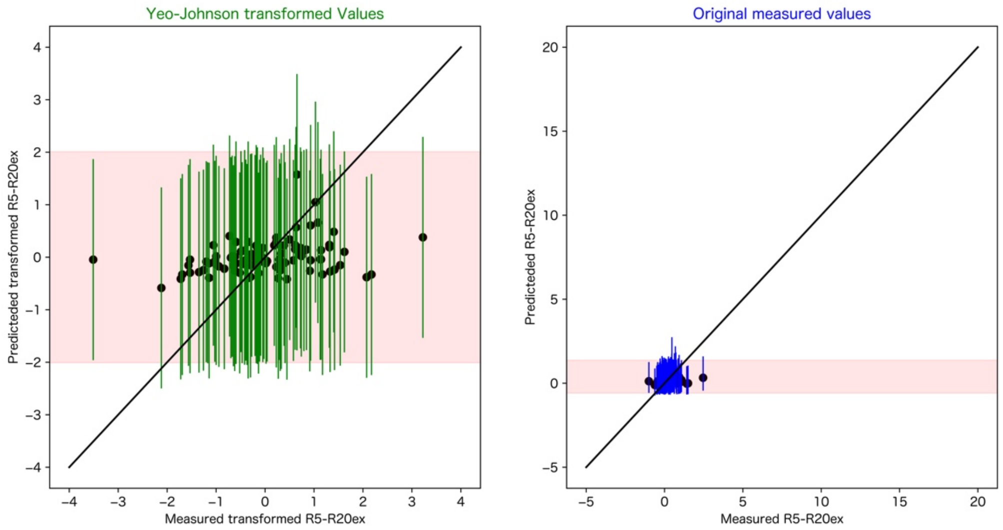

Male: Predicted vs Measured R5-R20delta

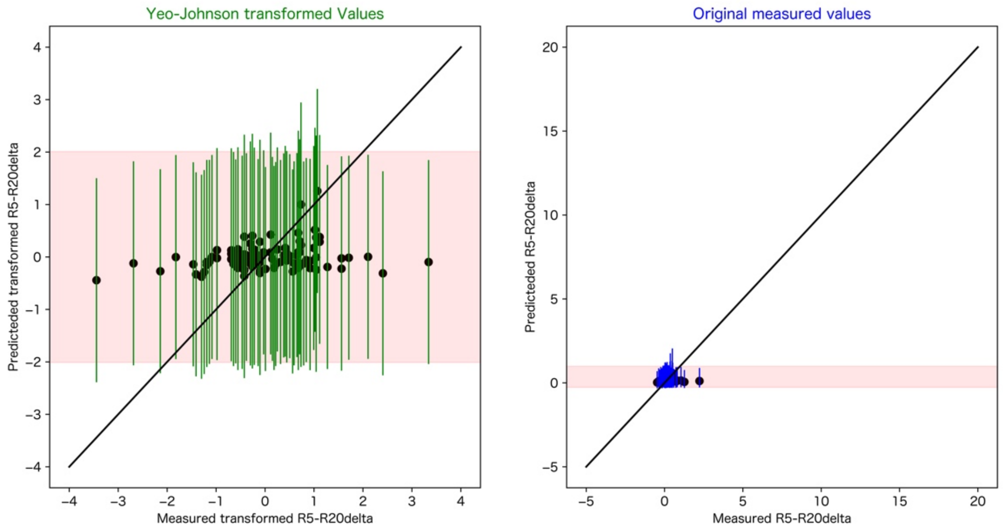

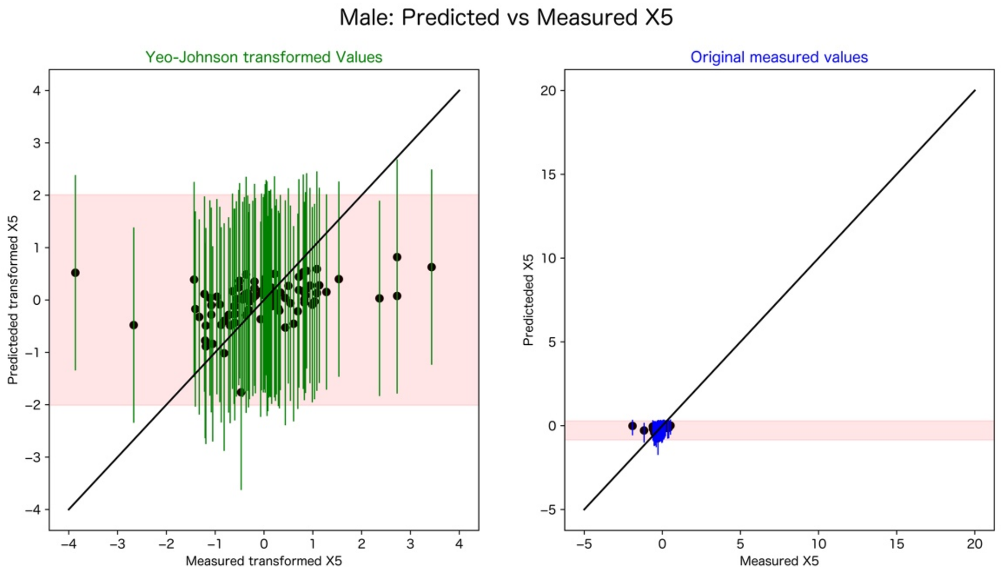
Male: Predicted vs Measured X5
Yeo-Johnson transformed Values
Original measured values
Predicteded transformed X5
Measured transformed X5
Predicteded X5
Measured X5

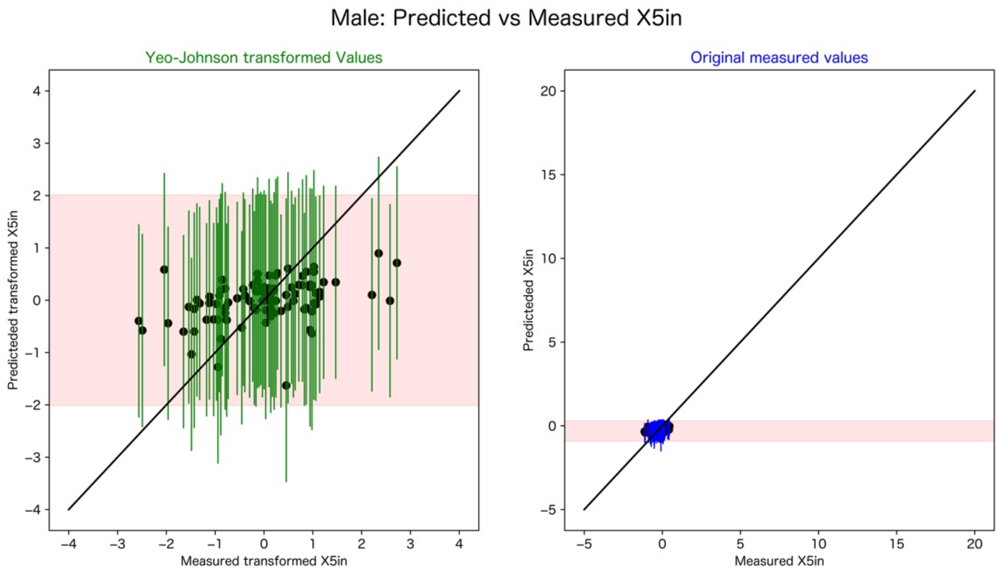
Male: Predicted vs Measured X5in
Yeo-Johnson transformed Values
Original measured values
Predicteded transformed X5in
Measured transformed X5in
Predicteded X5in
Measured X5in

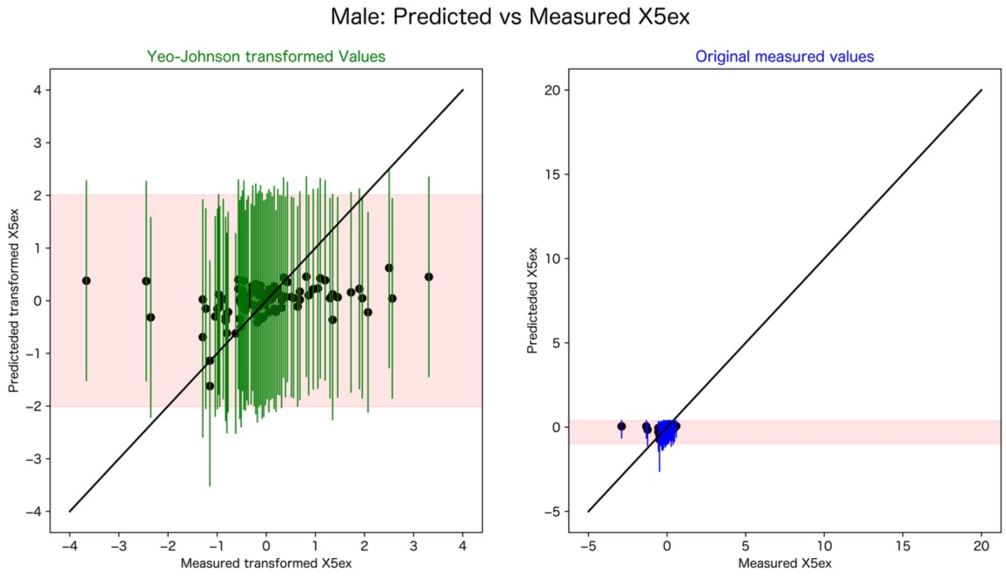
Male: Predicted vs Measured X5ex
Yeo-Johnson transformed Values
Original measured values
Predicteded transformed X5ex
Measured transformed X5ex
Predicteded X5ex
Measured X5ex

Male: Predicted vs Measured X5delta

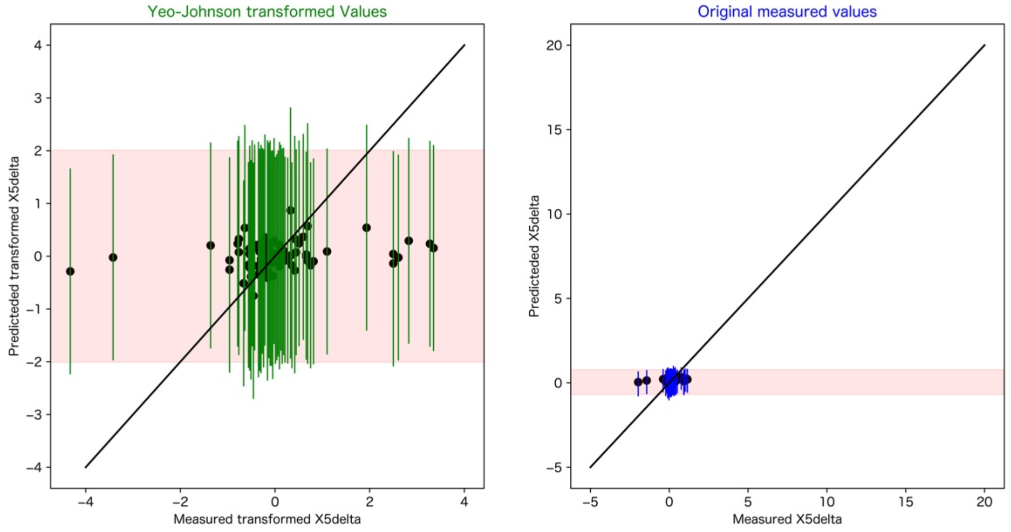

Male: Predicted vs Measured Fres

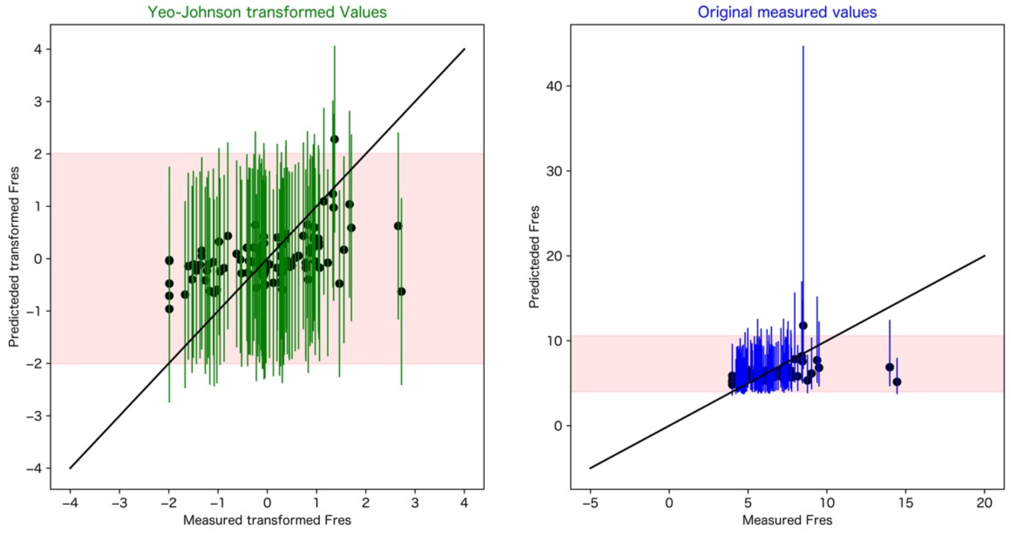

Male: Predicted vs Measured Fresin

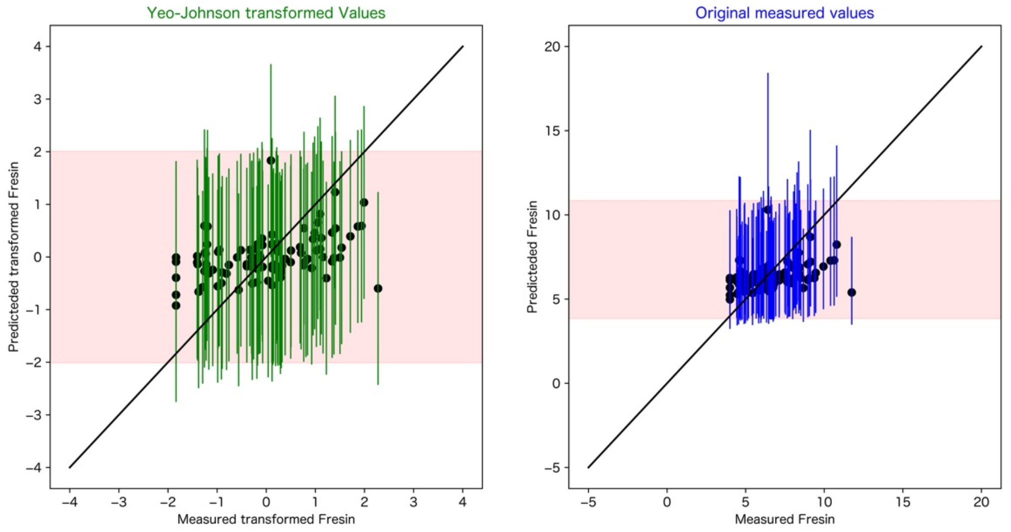

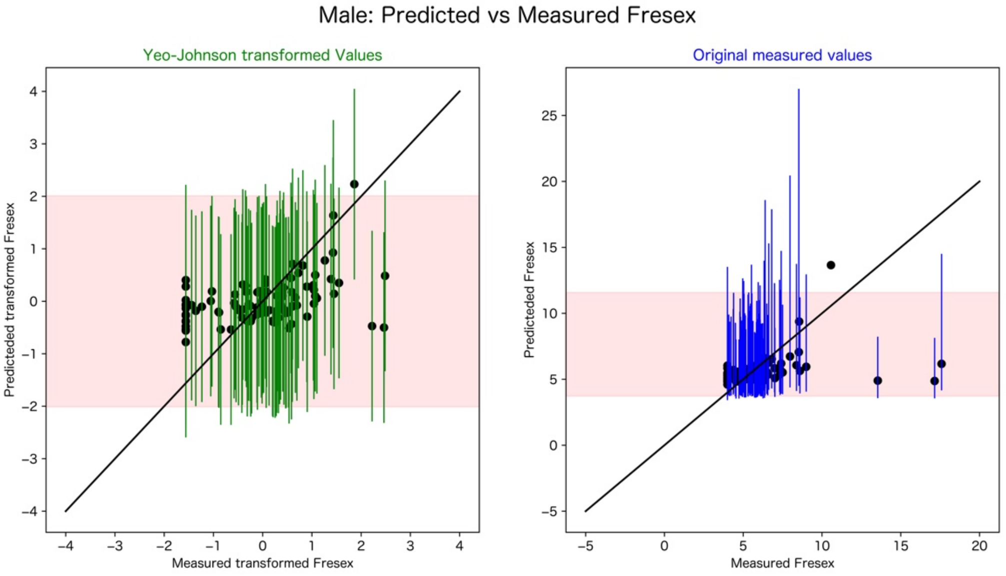
Male: Predicted vs Measured Fresex
Yeo-Johnson transformed Values
Original measured values
Predicteded transformed Fresex
Measured transformed Fresex
Predicteded Fresex
Measured Fresex

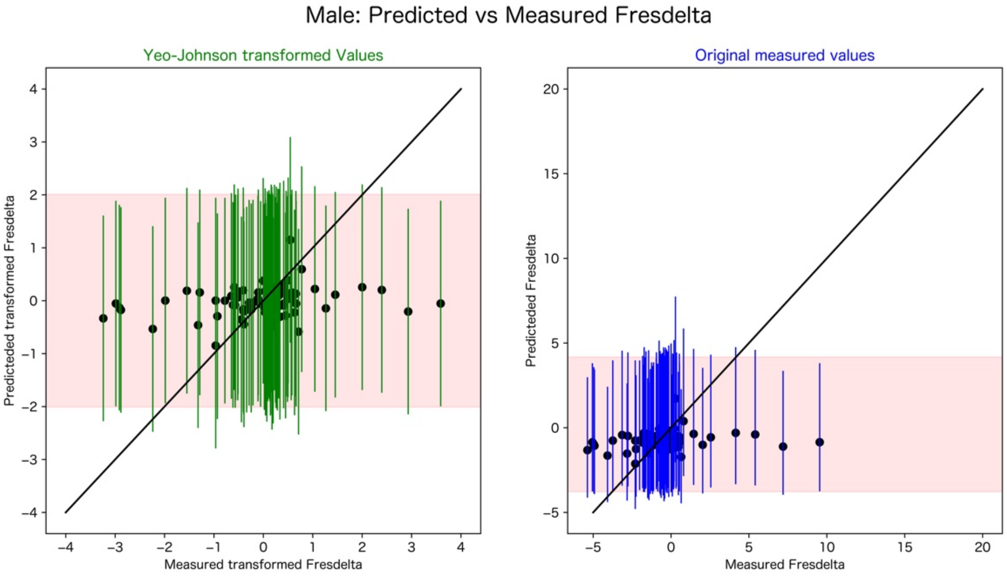
Male: Predicted vs Measured Fresdelta
Yeo-Johnson transformed Values
Original measured values
Predicteded transformed Fresdelta
Measured transformed Fresdelta
Predicteded Fresdelta
Measured Fresdelta

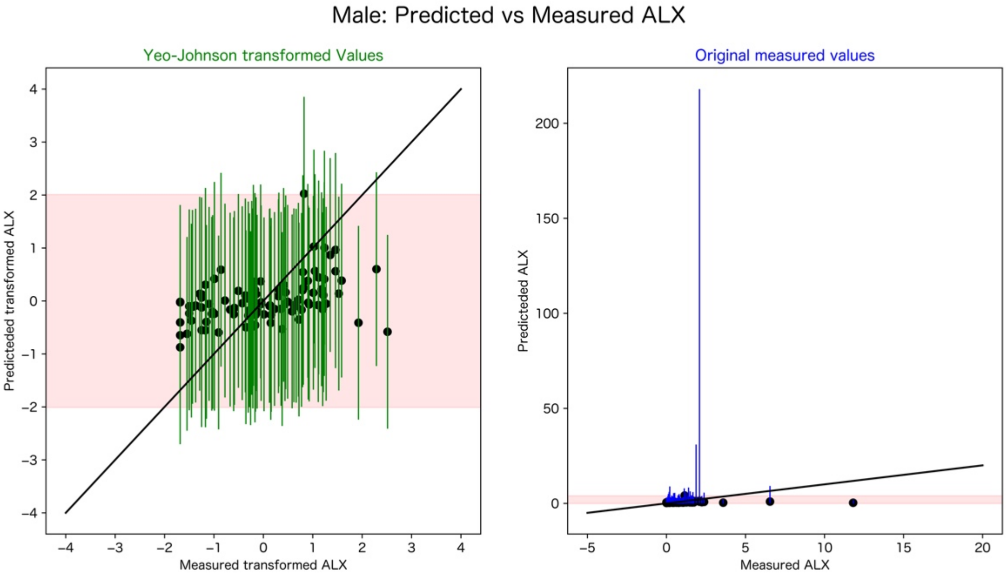
Male: Predicted vs Measured ALX
Yeo-Johnson transformed Values
Original measured values
Predicteded transformed ALX
Measured transformed ALX
Predicteded ALX
Measured ALX

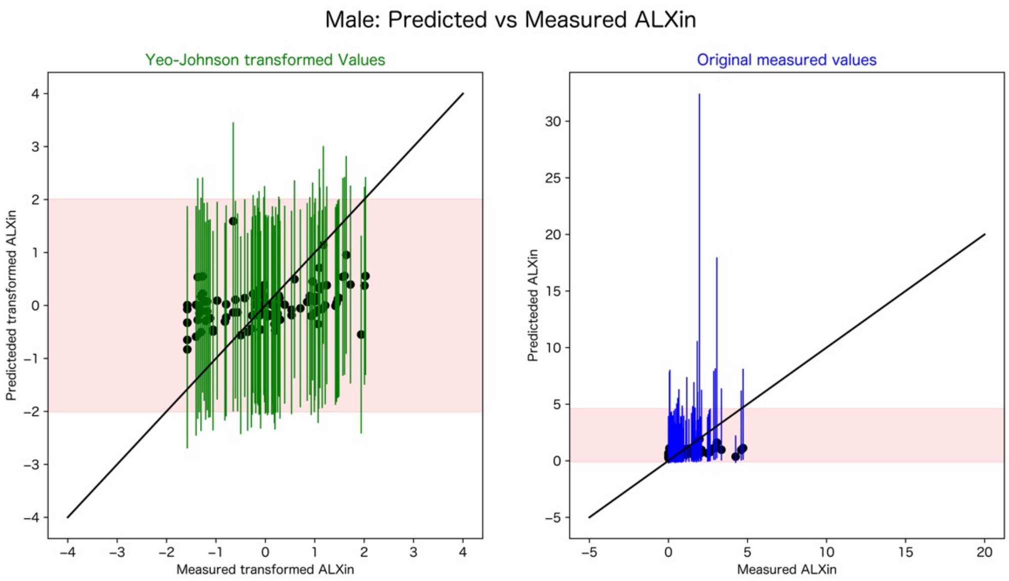
Male: Predicted vs Measured ALXin
Yeo-Johnson transformed Values
Original measured values
Predicteded transformed ALXin
Measured transformed ALXin
Predicteded ALXin
Measured ALXin

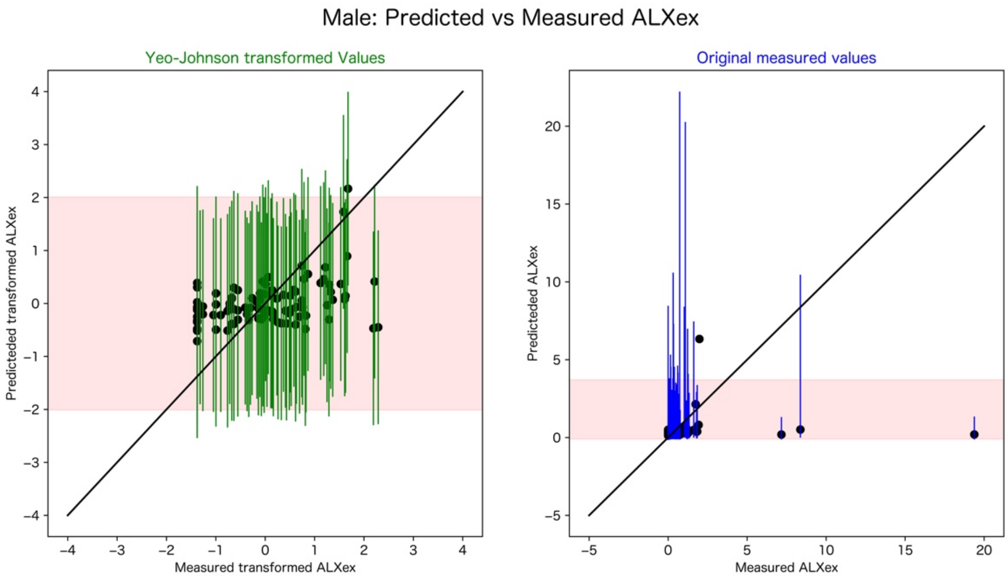
Male: Predicted vs Measured ALXex
Yeo-Johnson transformed Values
Original measured values
Predicteded transformed ALXex
Measured transformed ALXex
Predicteded ALXex
Measured ALXex

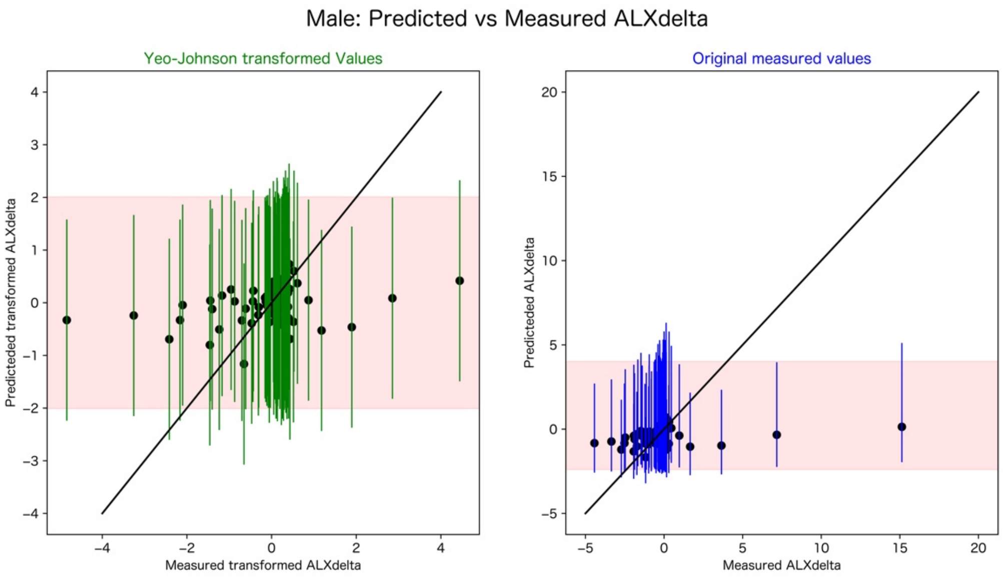
Male: Predicted vs Measured ALXdelta
Yeo-Johnson transformed Values
Original measured values
Predicteded transformed ALXdelta
Measured transformed ALXdelta
Predicteded ALXdelta
Measured ALXdelta

Female: Predicted vs Measured R5

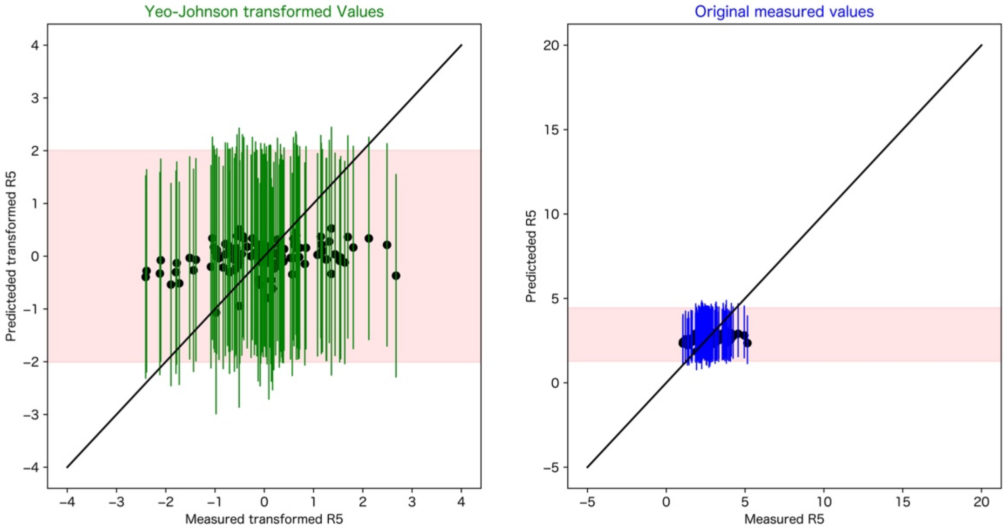

Female: Predicted vs Measured R5in

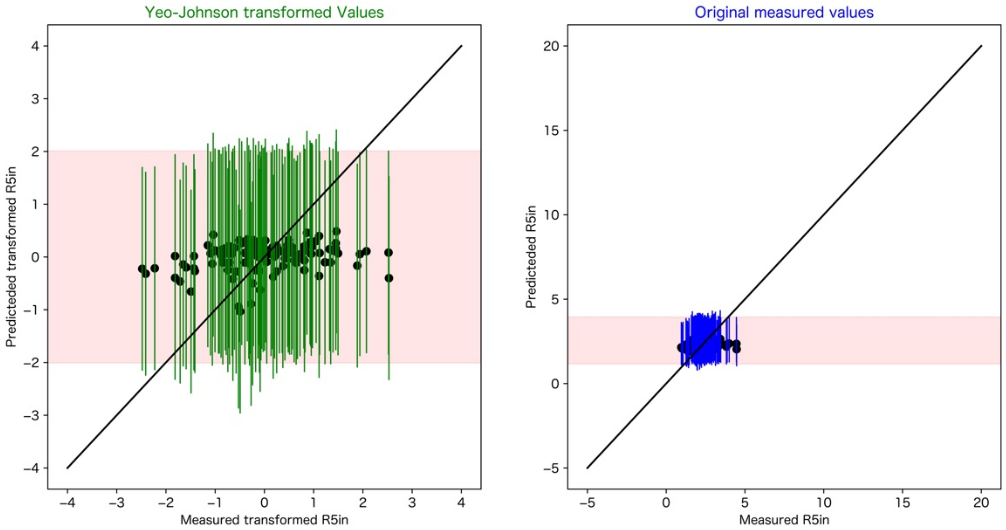

Female: Predicted vs Measured R5ex

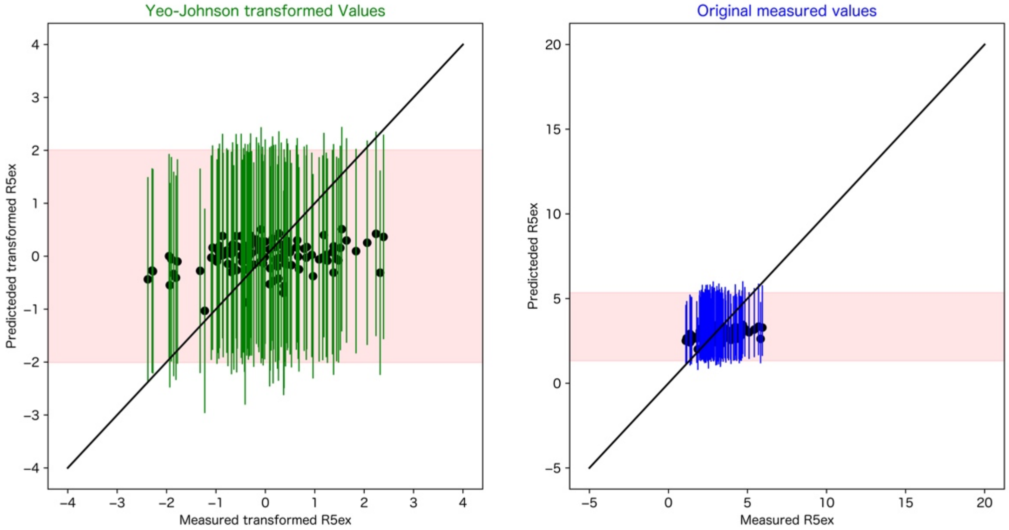

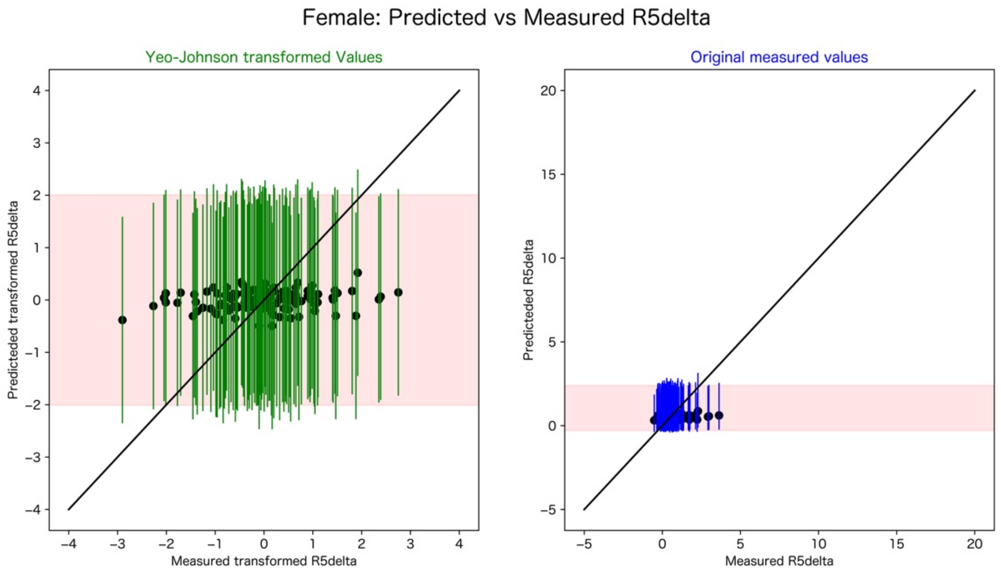
Female: Predicted vs Measured R5delta
Yeo-Johnson transformed Values
Original measured values
Predicteded transformed R5delta
Measured transformed R5delta
Predicteded R5delta
Measured R5delta

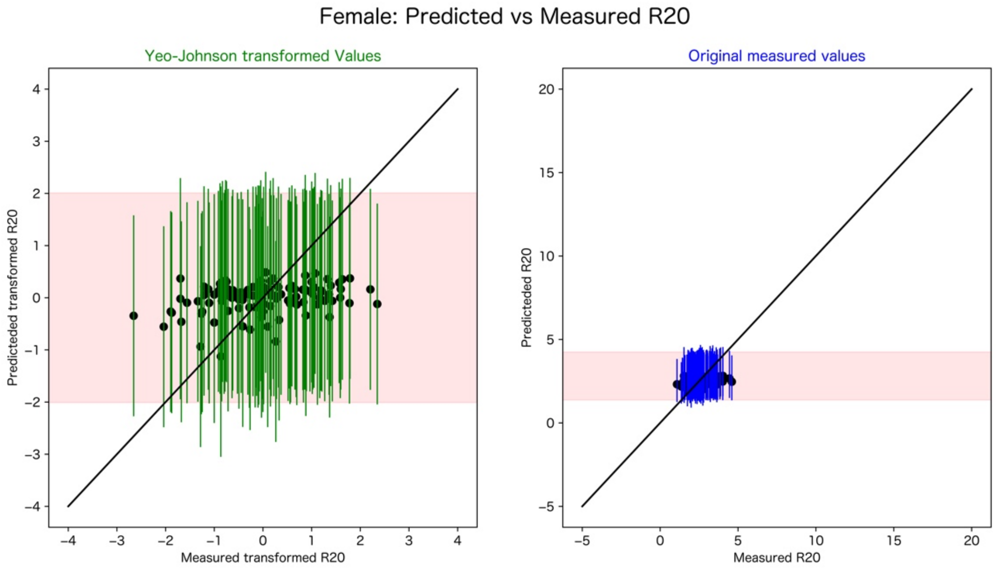
Female: Predicted vs Measured R20
Yeo-Johnson transformed Values
Original measured values
Predicteded transformed R20
Measured transformed R20
Predicteded R20
Measured R20

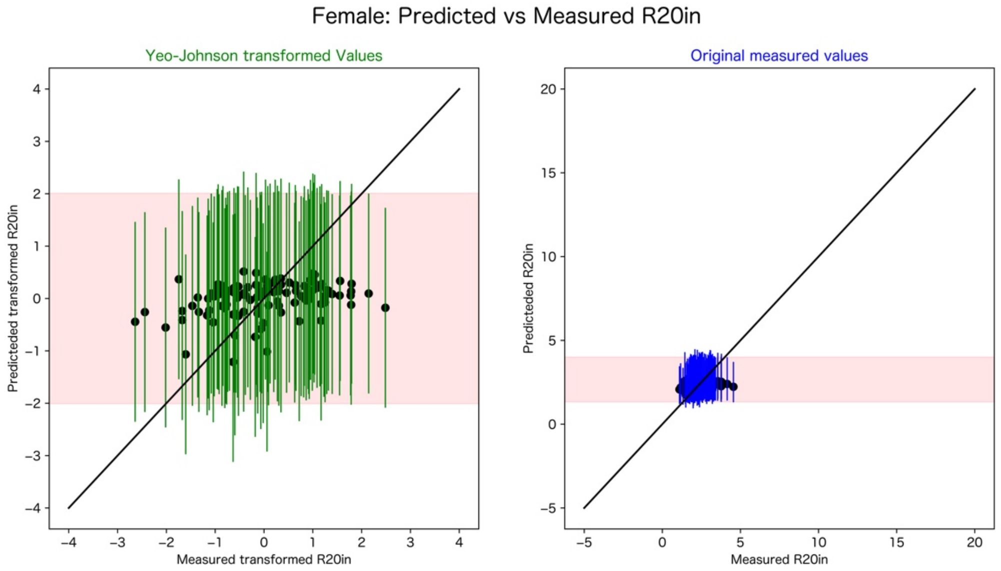
Female: Predicted vs Measured R20in
Yeo-Johnson transformed Values
Original measured values
Predicteded transformed R20in
Measured transformed R20in
Predicteded R20in
Measured R20in

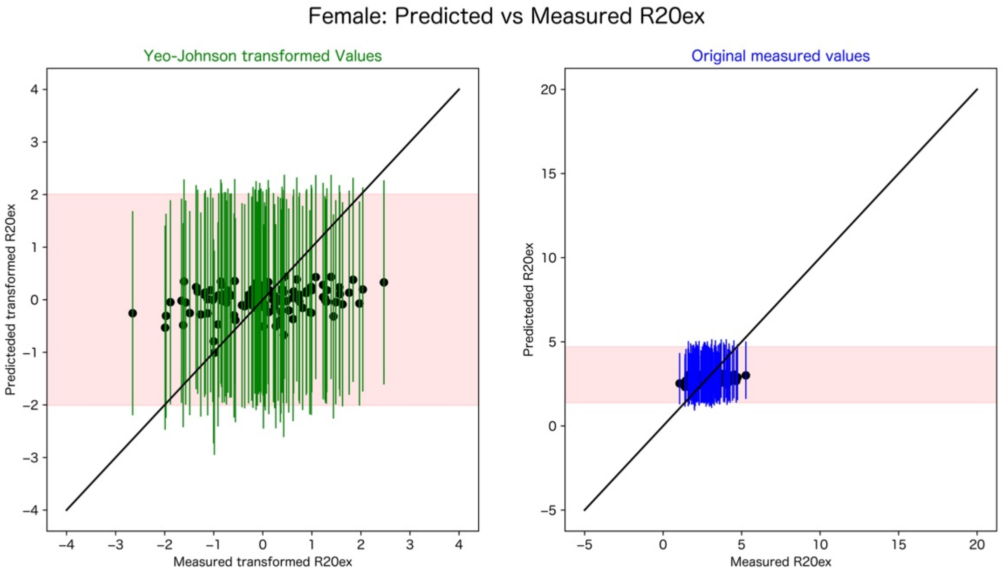

Female: Predicted vs Measured R20ex
Yeo-Johnson transformed Values
Original measured values
Predicteded transformed R20ex
Measured transformed R20ex
Predicteded R20ex
Measured R20ex

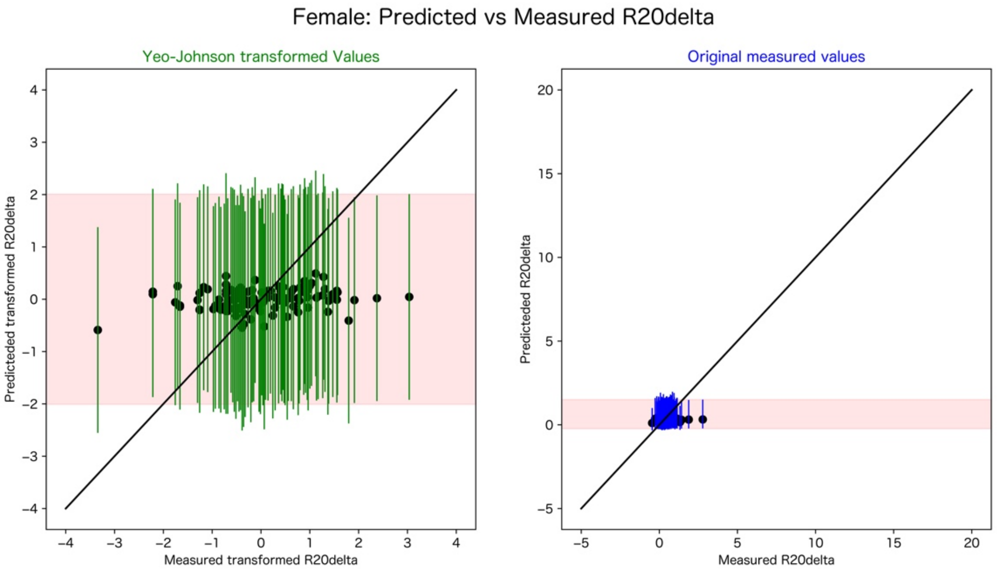

Female: Predicted vs Measured R20delta
Yeo-Johnson transformed Values
Original measured values
Predicteded transformed R20delta
Measured transformed R20delta
Predicteded R20delta
Measured R20delta

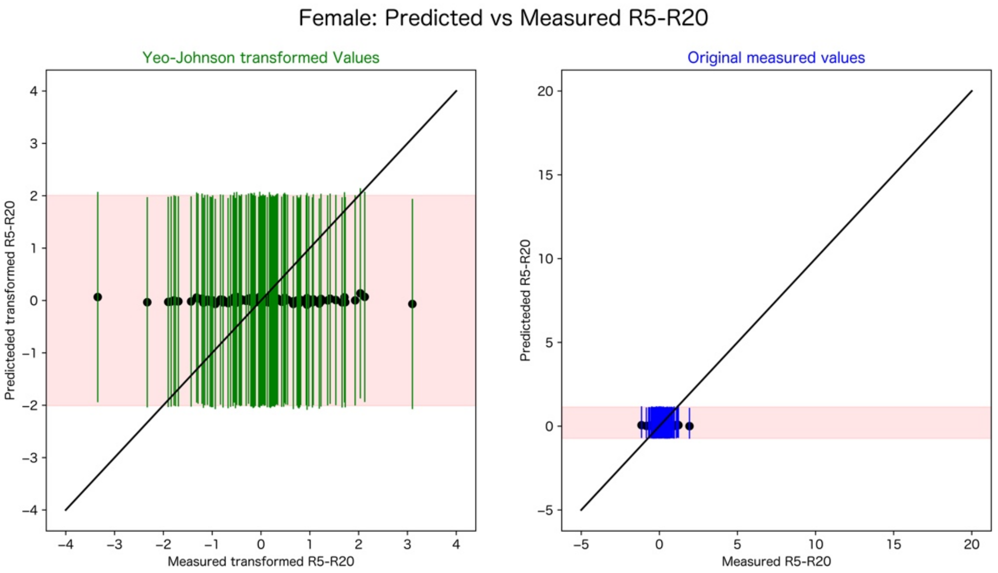

Female: Predicted vs Measured R5-R20
Yeo-Johnson transformed Values
Original measured values
Predicteded transformed R5-R20
Measured transformed R5-R20
Predicteded R5-R20
Measured R5-R20

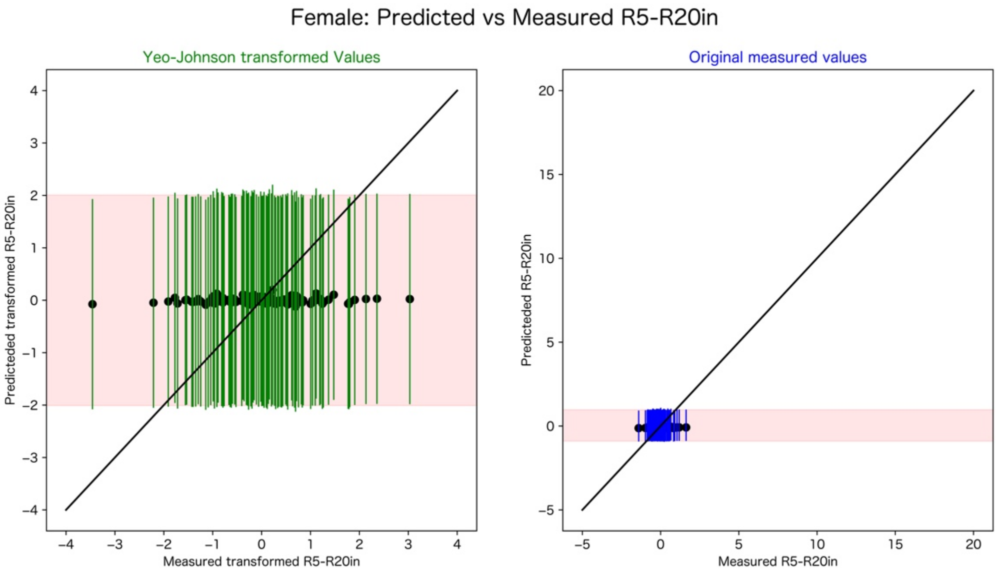
Female: Predicted vs Measured R5-R20in
Yeo-Johnson transformed Values
Original measured values
Predicteded transformed R5-R20in
Measured transformed R5-R20in
Predicteded R5-R20in
Measured R5-R20in

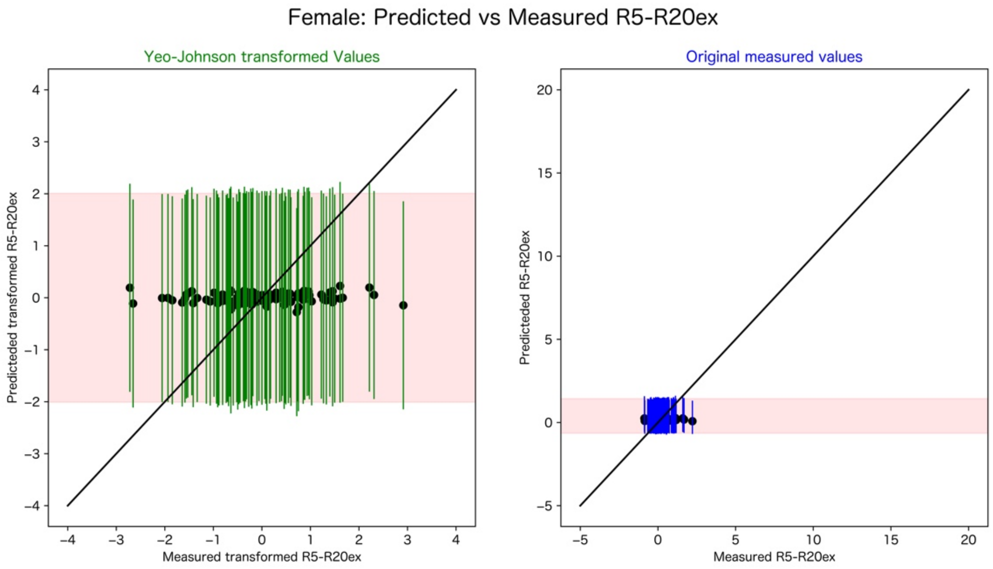
Female: Predicted vs Measured R5-R20ex
Yeo-Johnson transformed Values
Original measured values
Predicteded transformed R5-R20ex
Measured transformed R5-R20ex
Predicteded R5-R20ex
Measured R5-R20ex

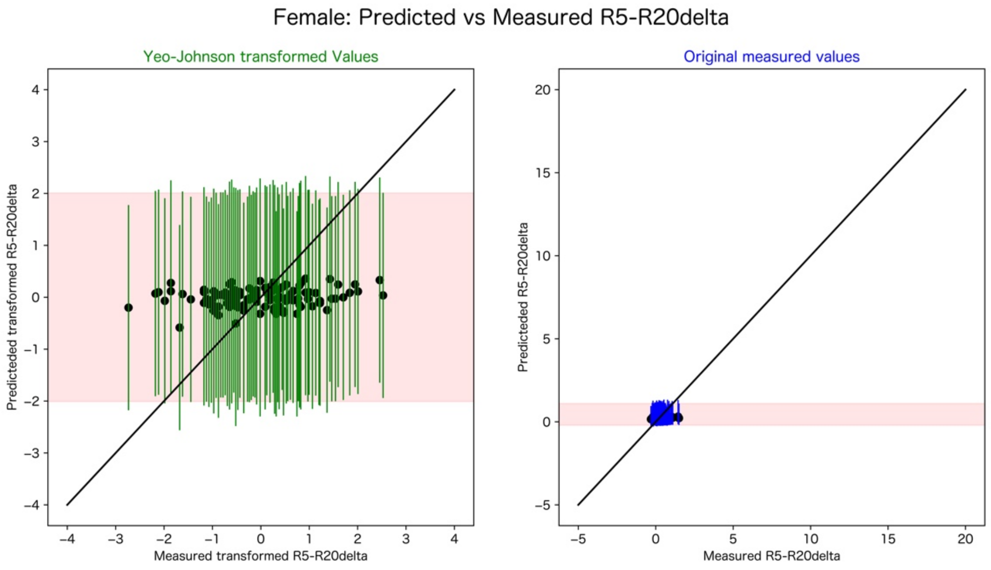
Female: Predicted vs Measured R5-R20delta
Yeo-Johnson transformed Values
Original measured values
Predicteded transformed R5-R20delta
Measured transformed R5-R20delta
Predicteded R5-R20delta
Measured R5-R20delta

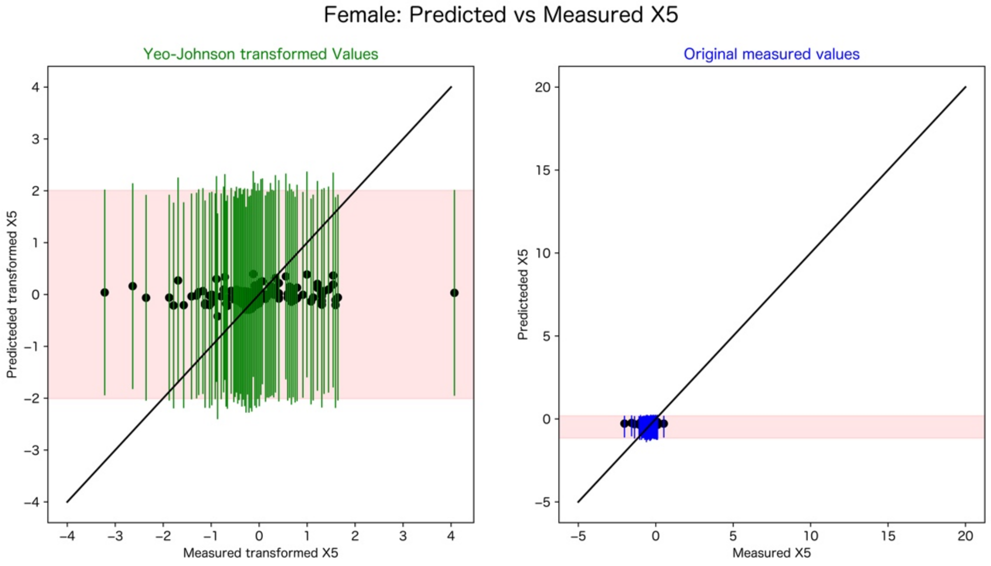
Female: Predicted vs Measured X5
Yeo-Johnson transformed Values
Predicteded transformed X5
Measured transformed X5
Original measured values
Predicteded X5
Measured X5

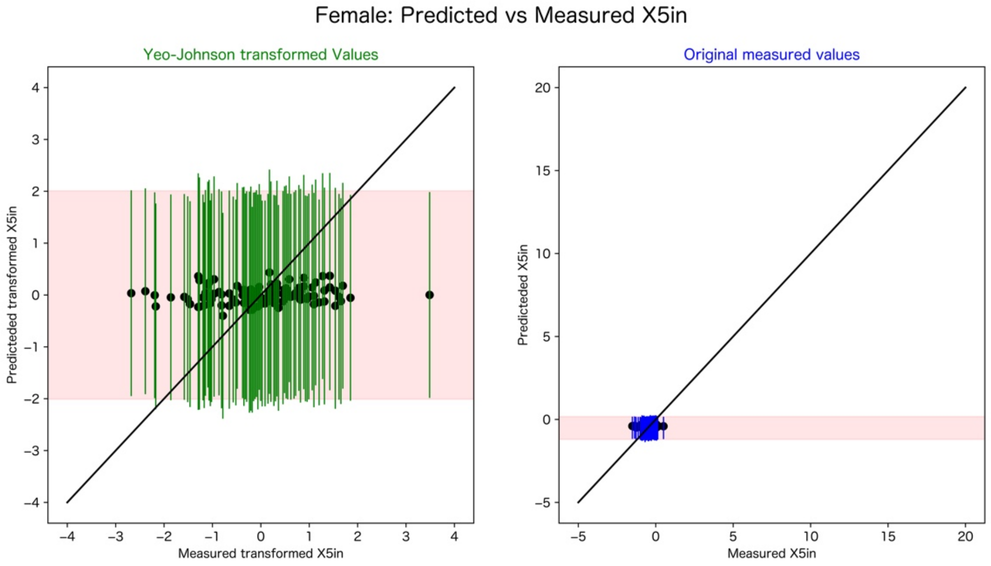
Female: Predicted vs Measured X5in
Yeo-Johnson transformed Values
Predicteded transformed X5in
Measured transformed X5in
Original measured values
Predicteded X5in
Measured X5in

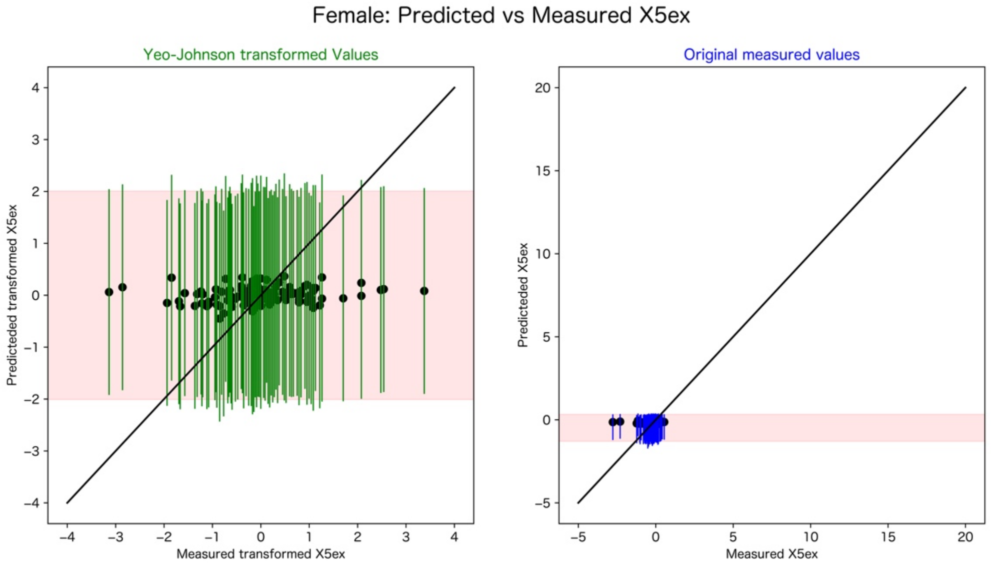
Female: Predicted vs Measured X5ex
Yeo-Johnson transformed Values
Predicteded transformed X5ex
Measured transformed X5ex
Original measured values
Predicteded X5ex
Measured X5ex

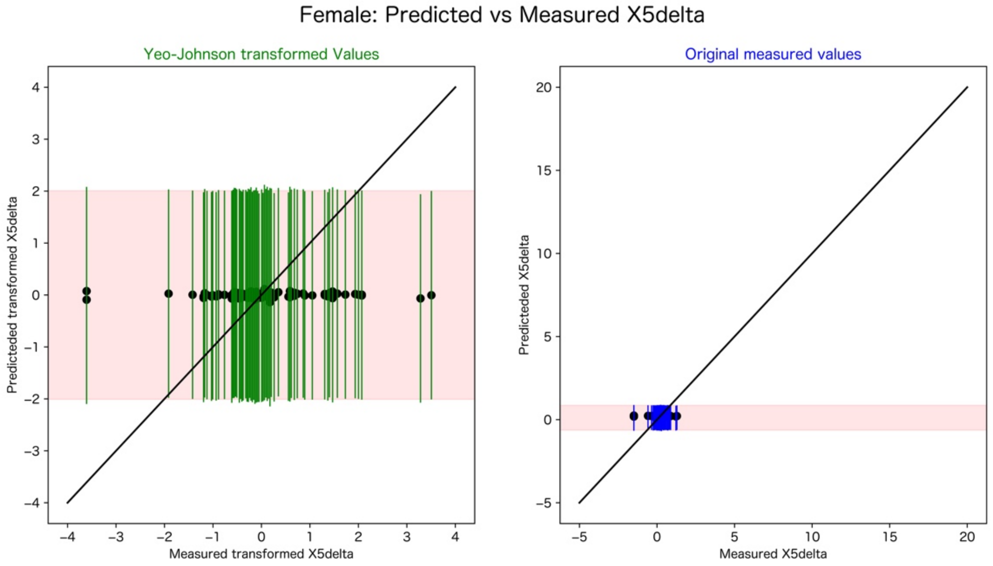
Female: Predicted vs Measured X5delta
Yeo-Johnson transformed Values
Predicteded transformed X5delta
Measured transformed X5delta
Original measured values
Predicteded X5delta
Measured X5delta

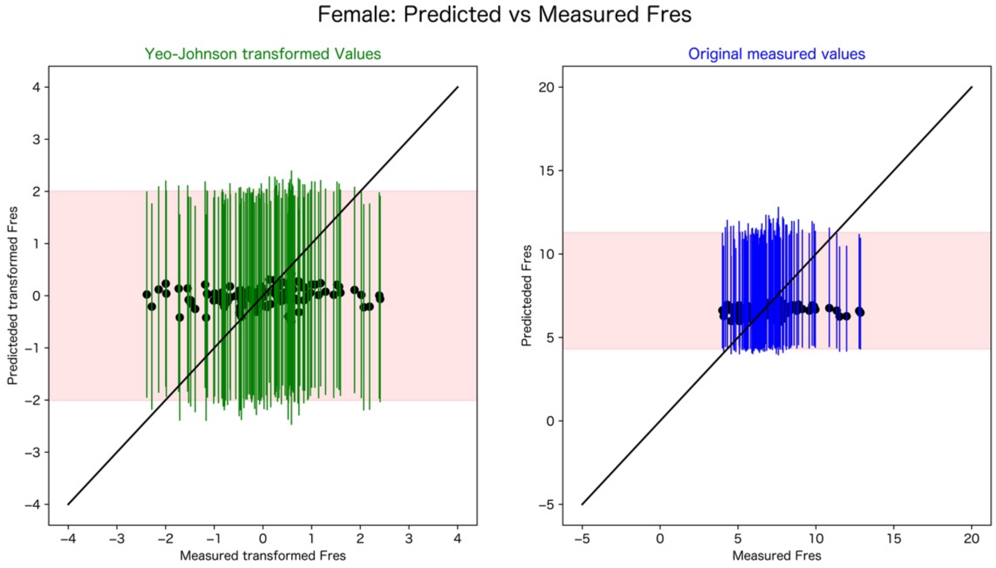
Female: Predicted vs Measured Fres
Yeo-Johnson transformed Values
Predicteded transformed Fres
Measured transformed Fres
Original measured values
Predicteded Fres
Measured Fres

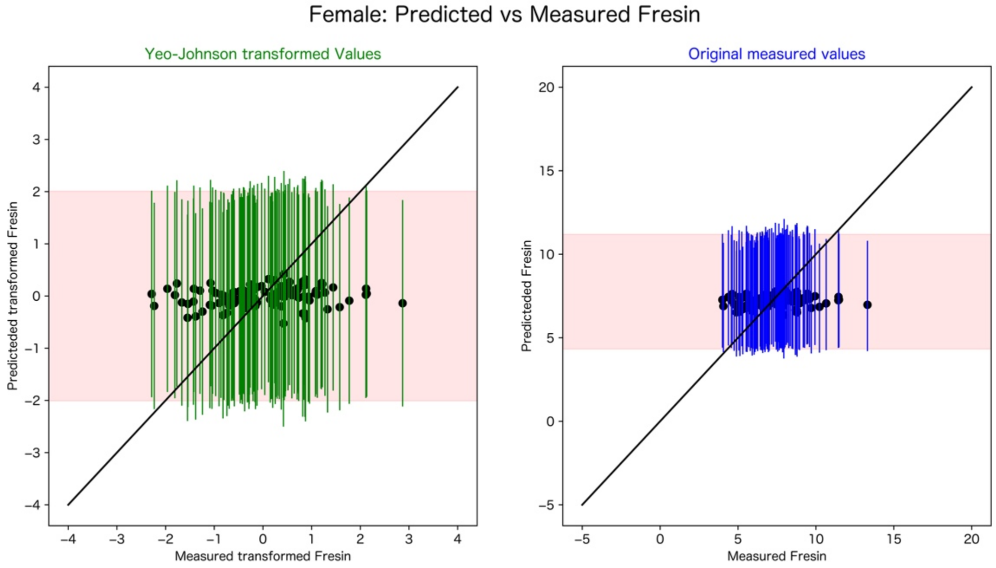
Female: Predicted vs Measured Fresin
Yeo-Johnson transformed Values
Predicteded transformed Fresin
Measured transformed Fresin
Original measured values
Predicteded Fresin
Measured Fresin

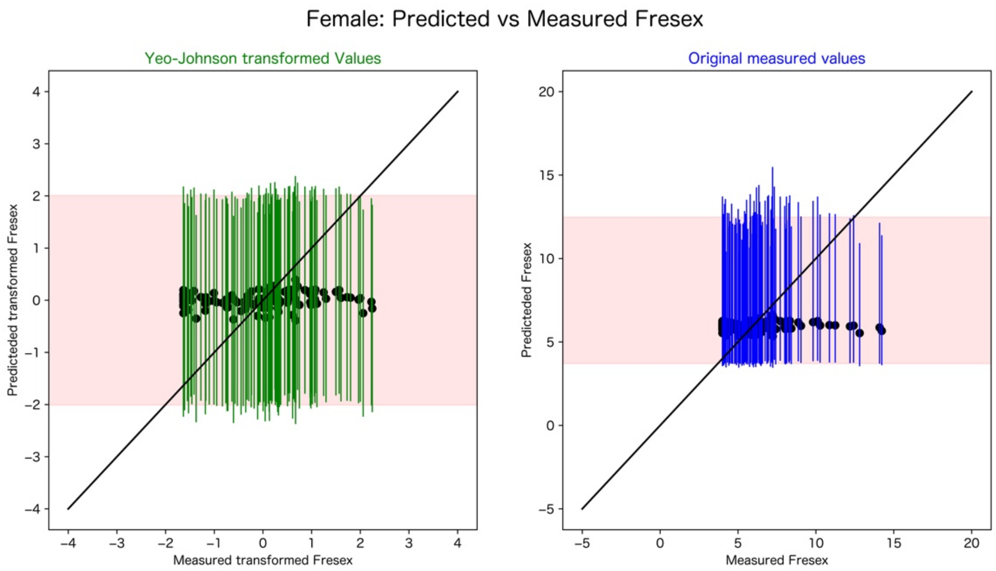
Female: Predicted vs Measured Fresex
Yeo-Johnson transformed Values
Original measured values
Predicteded transformed Fresex
Measured transformed Fresex
Predicteded Fresex
Measured Fresex

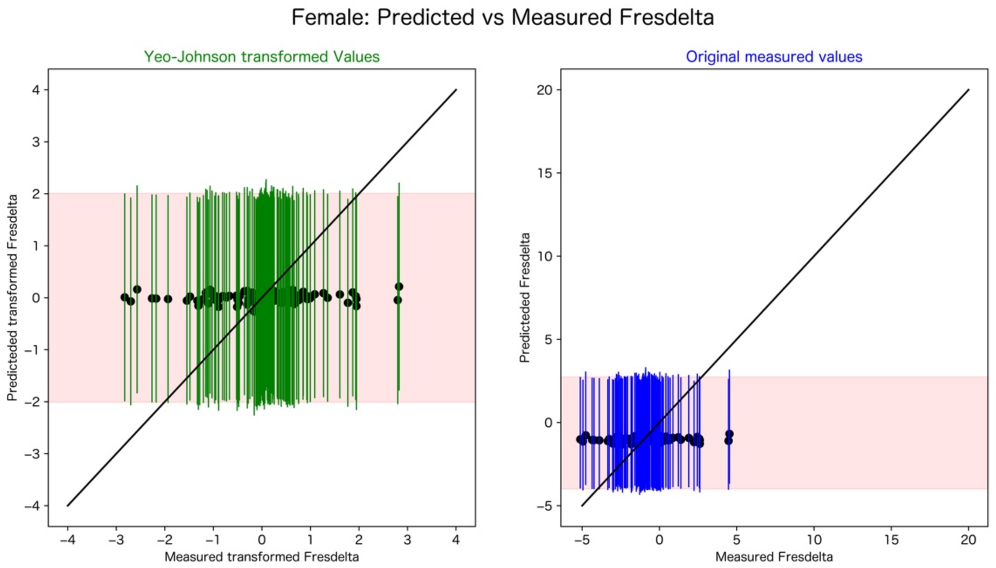
Female: Predicted vs Measured Fresdelta
Yeo-Johnson transformed Values
Original measured values
Predicteded transformed Fresdelta
Measured transformed Fresdelta
Predicteded Fresdelta
Measured Fresdelta

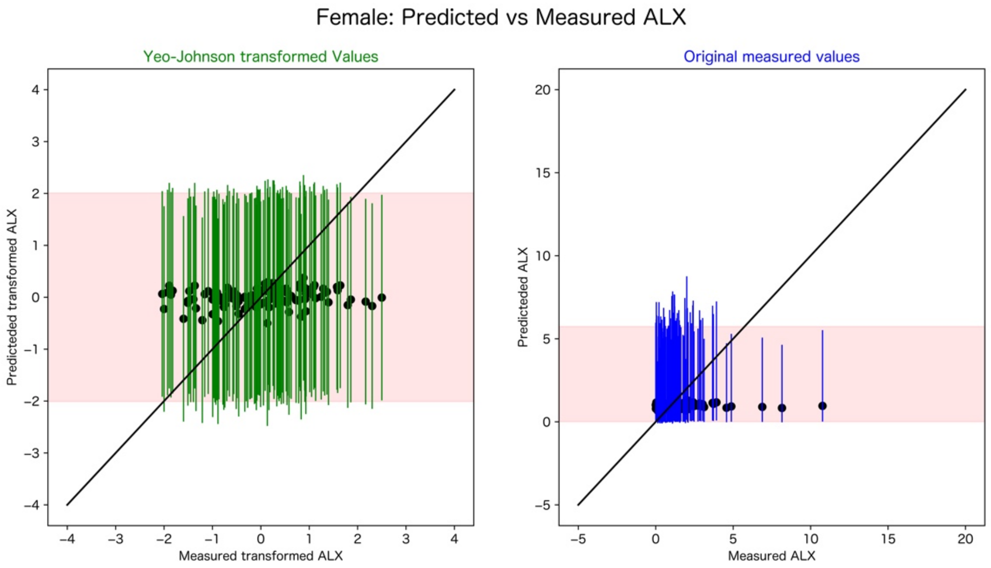
Female: Predicted vs Measured ALX
Yeo-Johnson transformed Values
Original measured values
Predicteded transformed ALX
Measured transformed ALX
Predicteded ALX
Measured ALX

Female: Predicted vs Measured ALXin

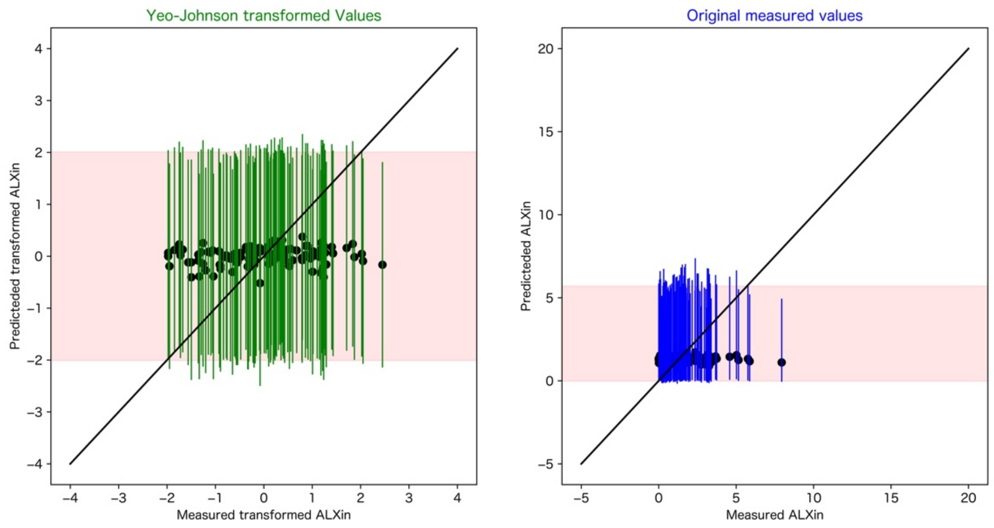

Female: Predicted vs Measured ALXex

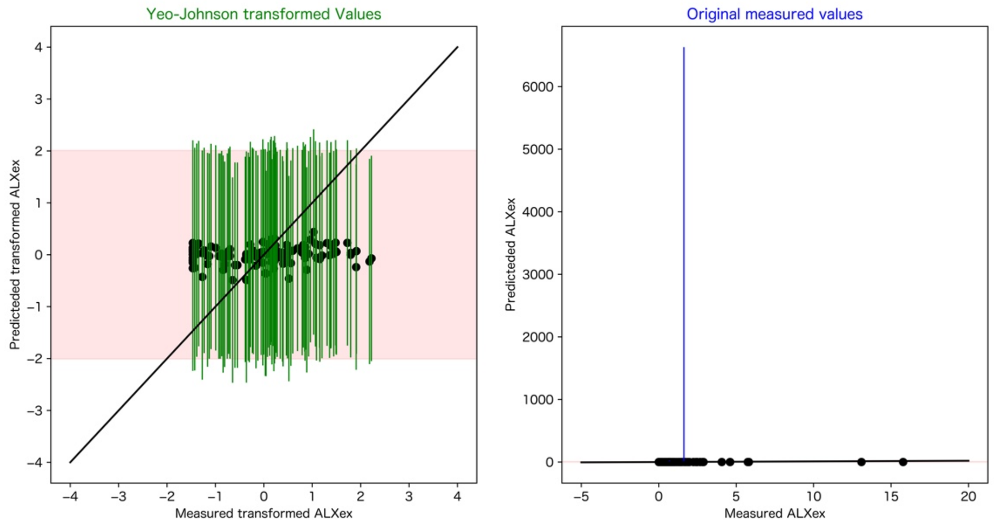

Female: Predicted vs Measured ALXdelta

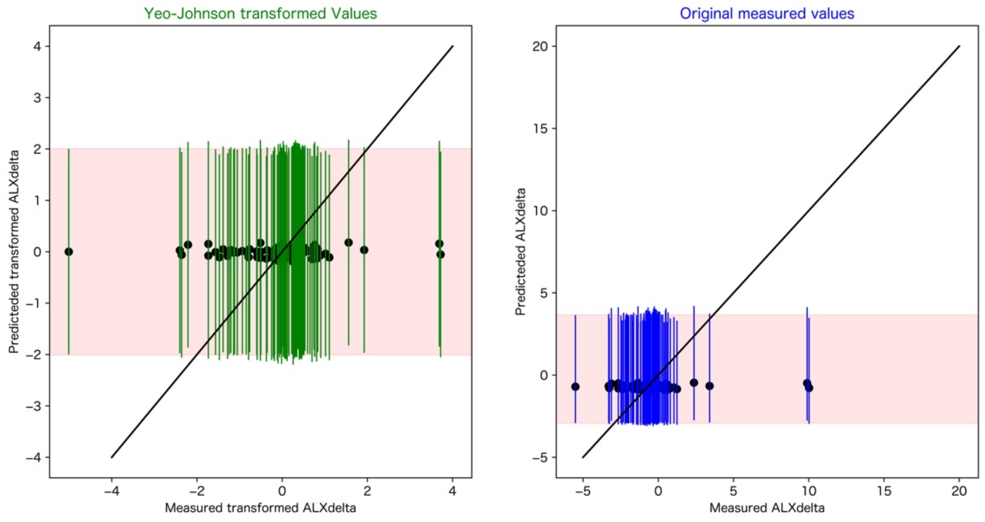

Supplementary Figure 3

Accuracy and loss during training and validation, and receiver operating characteristic (ROC) curve to discriminate between healthy controls and patients with asthma in each run with 5 layer deep learning model. Twenty controls and twenty patients were randomly selected as test data. Over-sampling with SMOTE was applied to the healthy controls' dataset in addition to weight balancing during training to handle imbalanced datasets for deep learning. The batch size was set to 32, validation split was set to 0.05, and the number of epochs was set to 1000. For input, sex and inhaled and exhaled data points for R5, R20, X5, Fres, and ALX from MostGraph measurements results were used. The metric scores were set to sensitivity, specificity, accuracy and F1 score at the cut-off point defined by the maximum Youden's index, and AUC.

These values were calculated and displayed in Python program "deeplearning-5layer-smote.py" available at https://github.com/sumi-yuki/mostgraph/blob/main/supplemental/deeplearning-5layer-smote.py

run 1

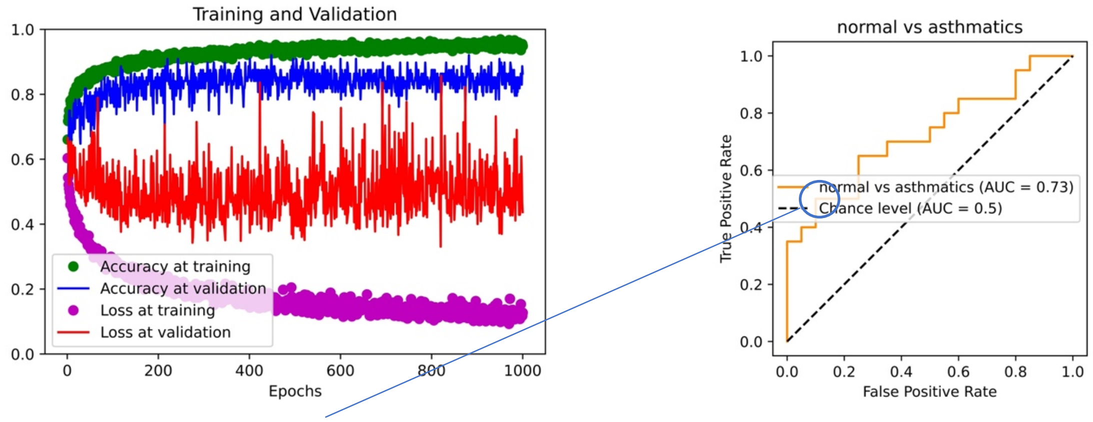

AUC 0.73, cut-off point 0.9949, sensitivity 0.50, specificity 0.90, accuracy 0.70, F1 score 0.625

run 2

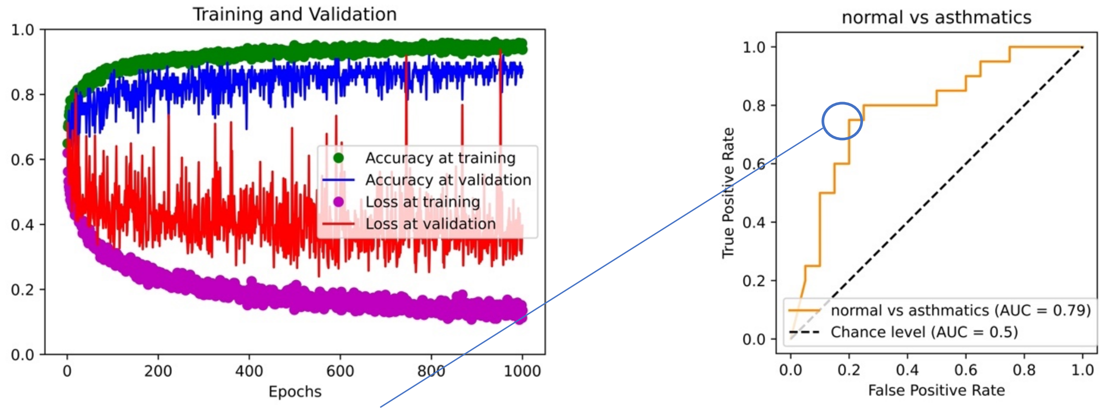

AUC 0.79, cut-off point 0.96166474, sensitivity 0.75, specificity 0.80, accuracy 0.78, F1 score 0.77

run 3

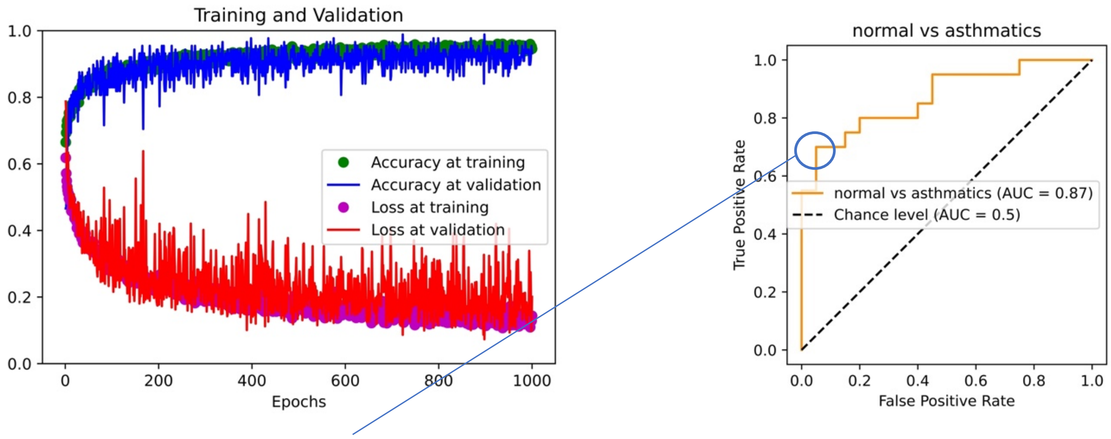

AUC 0.87, cut-off point 0.93333333, sensitivity 0.70, specificity 0.95, accuracy 0.83, F1 score 0.80

run 4

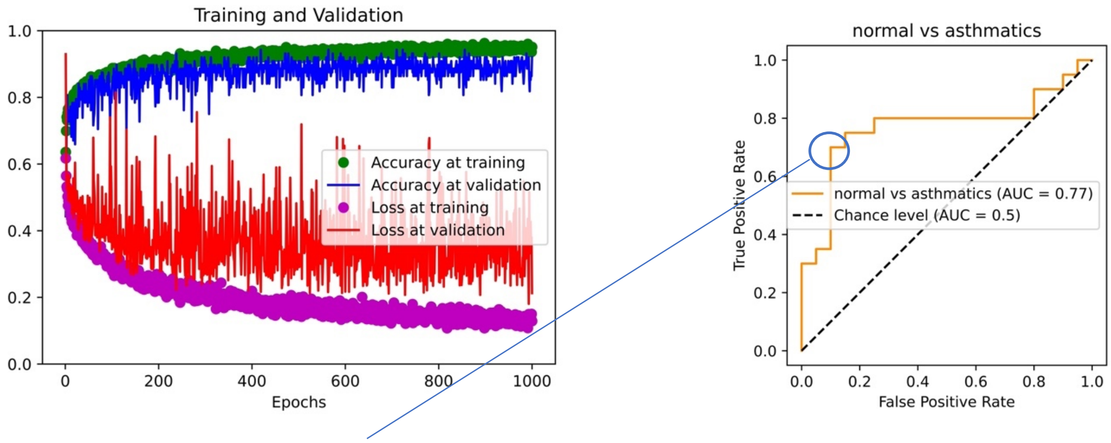

AUC 0.87, cut-off point 0.95024437, sensitivity 0.75, specificity 0.90, accuracy 0.83, F1 score 0.81

run 5

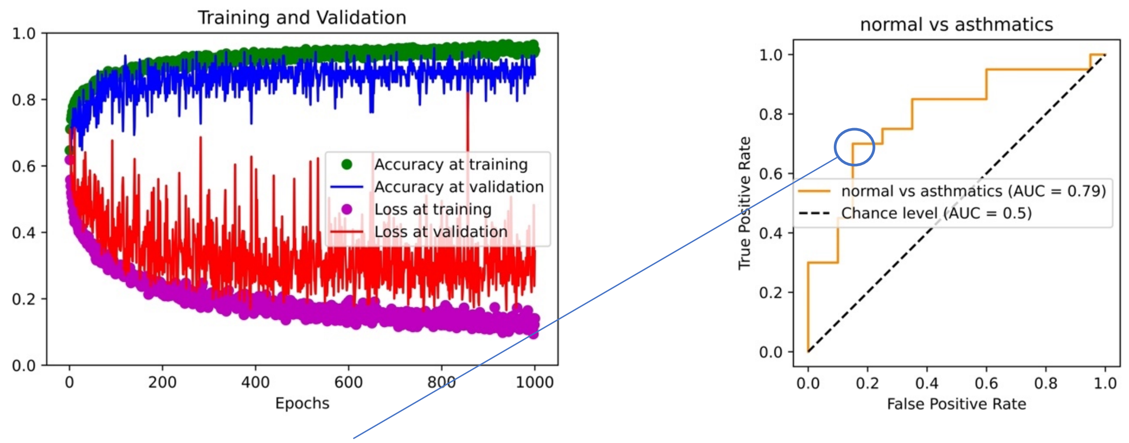

AUC 0.79, cut-off point 0.9849976, sensitivity 0.70, specificity 0.85, accuracy 0.77, F1 score 0.76

run 6

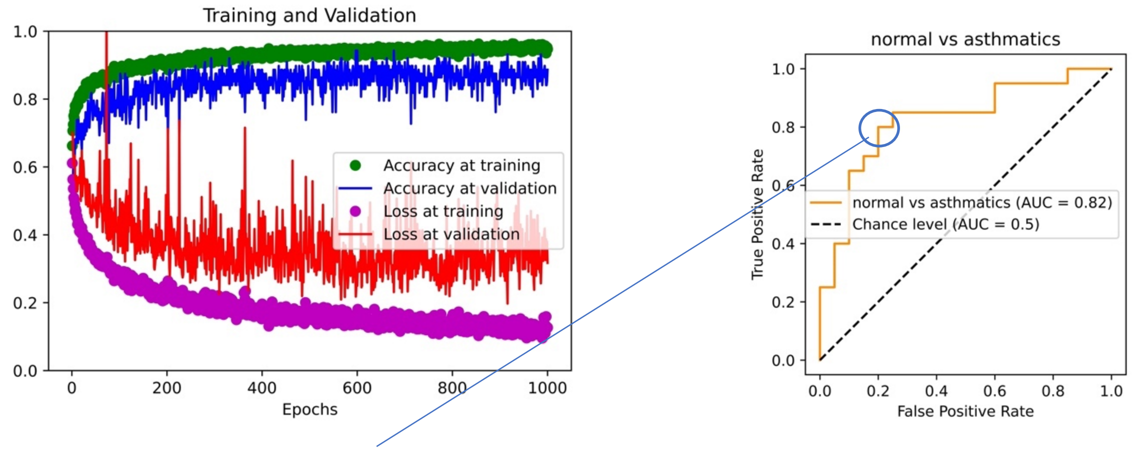

AUC 0.82, cut-off point 0.98102874, sensitivity 0.80, specificity 0.80, accuracy 0.80, F1 score 0.80

Supplementary Figure 4

Accuracy and loss during training and validation, and receiver operating characteristic (ROC) curve to discriminate between healthy controls and patients with asthma in each run with 1 layer neural network model. Twenty controls and twenty patients were randomly selected as test data. Over-sampling with SMOTE was applied to the healthy controls' dataset in addition to weight balancing during training to handle imbalanced datasets for deep learning. The batch size was set to 32, validation split was set to 0.05, and the number of epochs was set to 1000. For input, sex and inhaled and exhaled data points for R5, R20, X5, Fres, and ALX from MostGraph measurements results were used. The metric scores were sensitivity, specificity, accuracy and F1 score at the cut-off point defined by the maximum Youden's index, and AUC.

These values were calculated and displayed in Python program "deeplearning-mono-smote.py" available at https://github.com/sumi-yuki/mostgraph/blob/main/supplemental/deeplearning-mono-smote.py

run 1

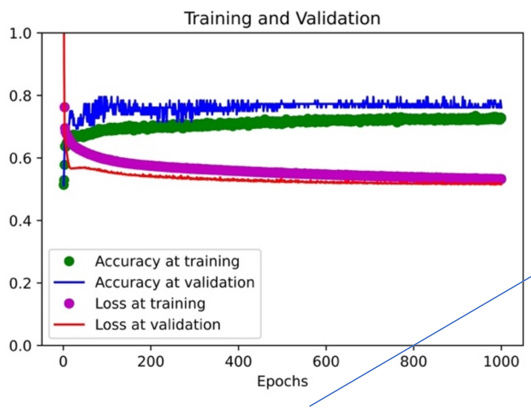

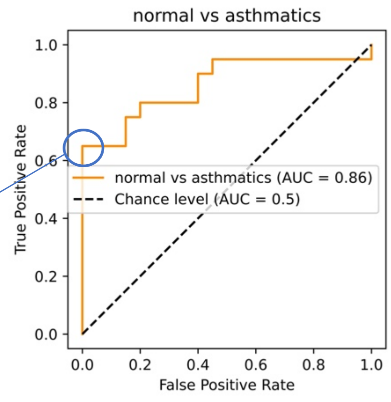

AUC 0.86, cut-off point 0.67276317, sensitivity 0.65, specificity 1.00, accuracy 0.83, F1 score 0.79

run 2

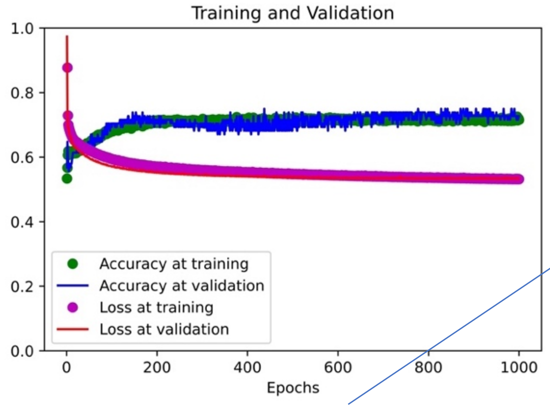

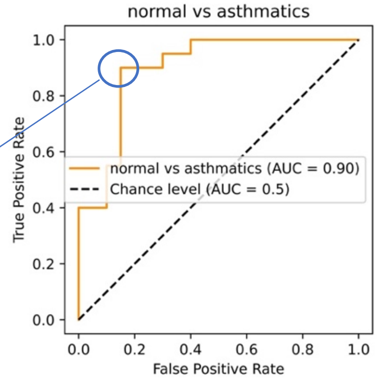

AUC 0.90, cut-off point 0.5873046, sensitivity 0.90, specificity 0.85, accuracy 0.88, F1 score 0.88

run 3

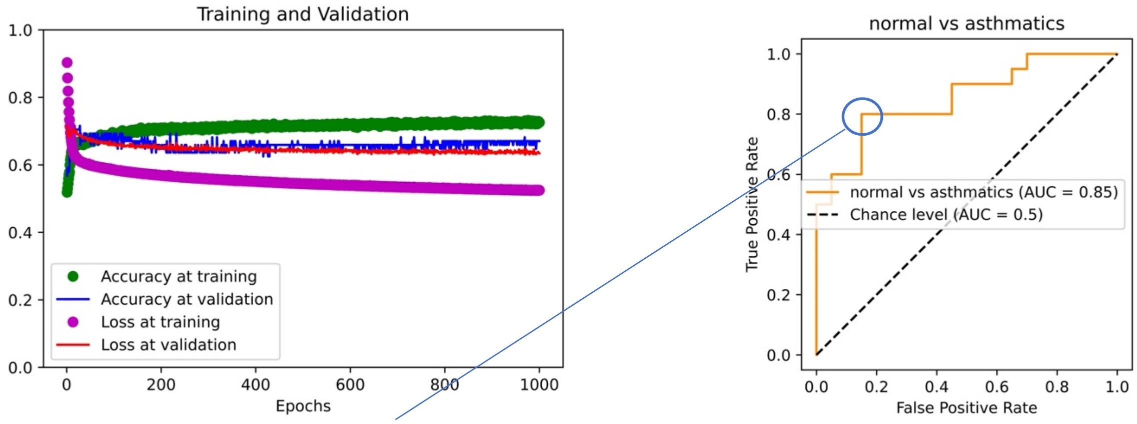

AUC 0.85, <u>cut-off point 0.52963644</u>, sensitivity 0.80, specificity 0.85, accuracy 0.83, F1 score 0.82

run 4

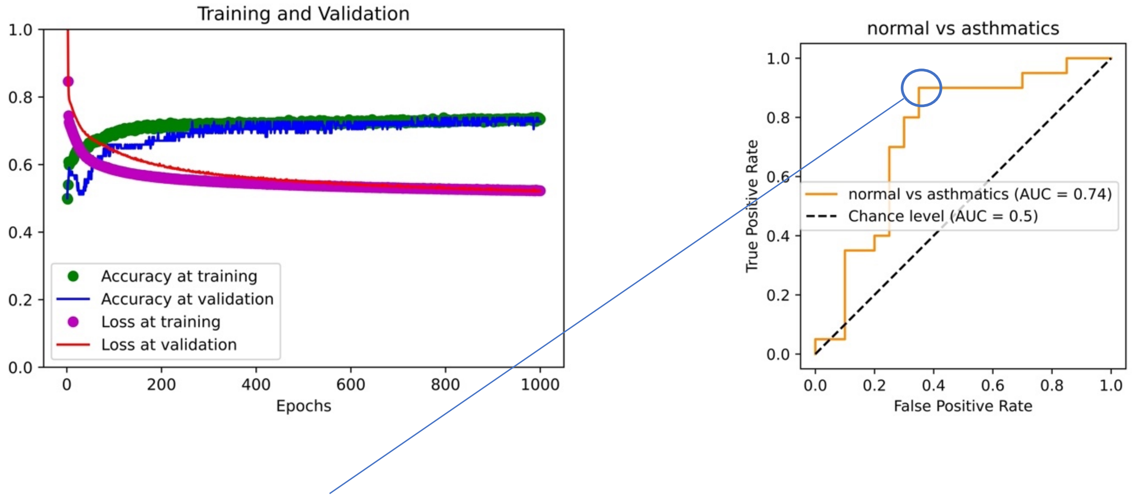

AUC 0.74, <u>cut-off point 0.35198024</u>, sensitivity 0.90, specificity 0.65, accuracy 0.78, F1 score 0.80

run 5

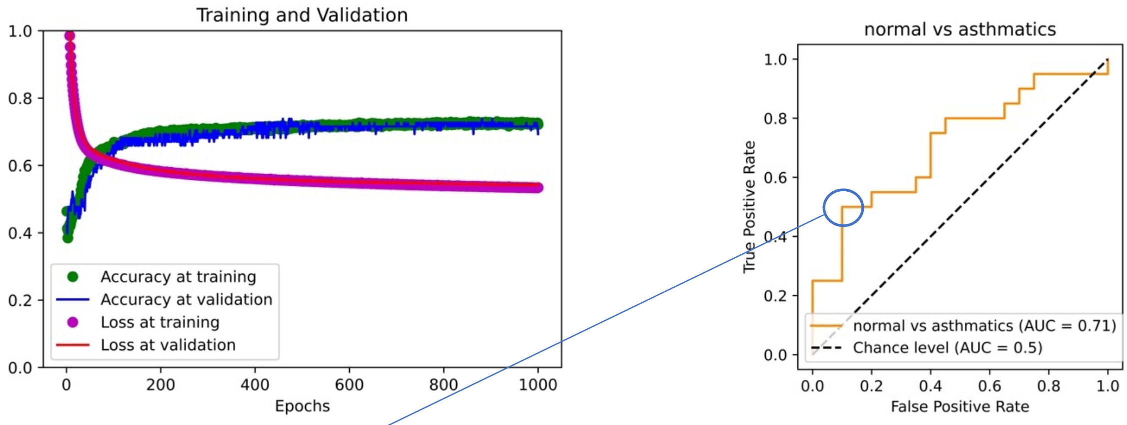

AUC 0.71, <u>cut-off point 0.7090467</u>, sensitivity 0.50, specificity 0.90, accuracy 0.70, F1 score 0.63

run 6

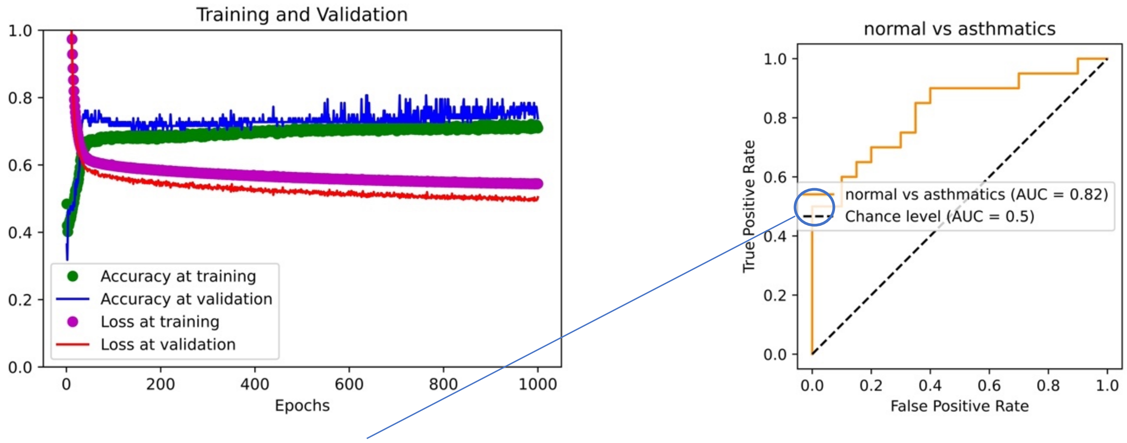

AUC 0.82, <u>cut-off point 0.6820854</u>, sensitivity 0.50, specificity 1.0, accuracy 0.75, F1 score 0.67

Supplementary Figure 5

Receiver operating characteristic (ROC) curve to discriminate between healthy controls and patients with asthma in each run with logistic regression model. Twenty controls and twenty patients were randomly selected as test data. Over-sampling with SMOTE was applied to the healthy controls' dataset. For input, sex and inhaled and exhaled data points for R5, R20, X5, Fres, and ALX from MostGraph measurements results were used. The metric scores were sensitivity, specificity, accuracy and F1 score at the cut-off point defined by the maximum Youden's index, and AUC.

These values were calculated and displayed in Python program "logistic-regression-smote.py" available at https://github.com/sumi-yuki/mostgraph/blob/main/supplemental/logistic-regression-smote.py

run 1

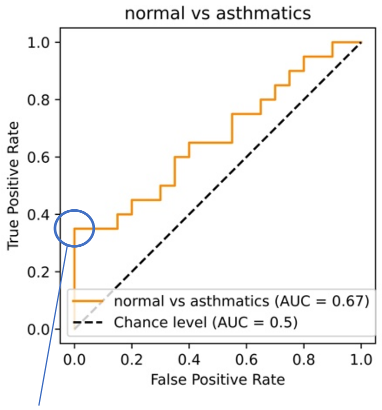

AUC 0.67, cut-off point 0.72747478, sensitivity 0.35, specificity 1.00, accuracy 0.68, F1 score 0.52

run 2

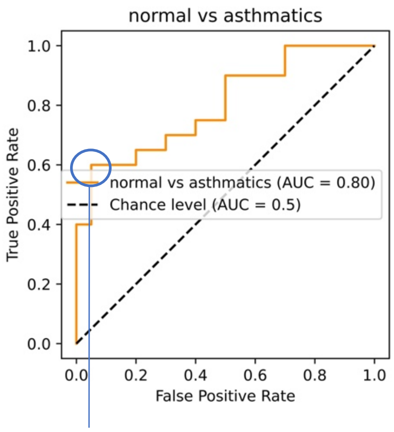

AUC 0.80, cut-off point 0.645014, sensitivity 0.60, specificity 0.95, accuracy 0.77, F1 score 0.73

run 3

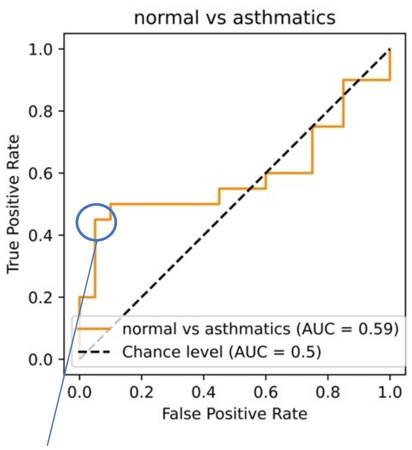

AUC 0.59, cut-off point 0.72446888, sensitivity 0.45, specificity 0.95, accuracy 0.70, F1 score 0.60

run 4

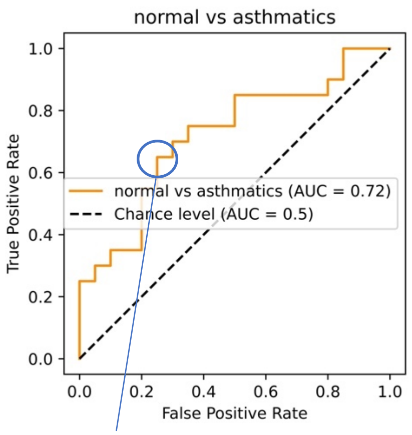

AUC 0.72, cut-off point 0.483244, sensitivity 0.65, specificity 0.75, accuracy 0.68, F1 score 0.68

run 5

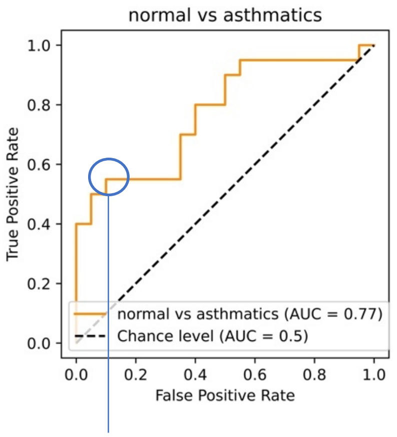

AUC 0.78, cut-off point 0.604683, sensitivity 0.55, specificity 0.90, accuracy 0.73, F1 score 0.67

run 6

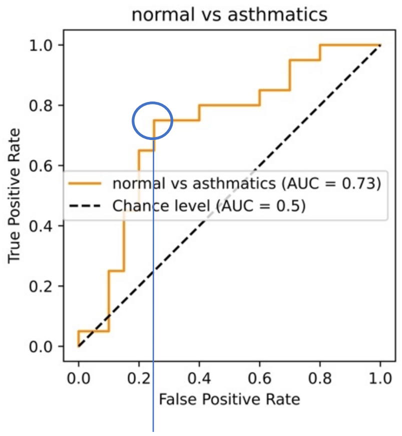

AUC 0.73, cut-off point 0.48751433, sensitivity 0.75, specificity 0.75, accuracy 0.75, F1 score 0.62

Supplementary Figure 6

Accuracy and loss during training and validation, and receiver operating characteristic (ROC) curve to discriminate between healthy controls and patients with asthma in each run with 5 layer deep learning model. Twenty controls and twenty patients were randomly selected as test data. Downsampling was applied to the asthmatics dataset in addition to weight balancing during training to handle imbalanced datasets for deep learning. The batch size was set to 32, validation split was set to 0.05, and the number of epochs was set to 1000. For input, sex and inhaled and exhaled data points for R5, R20, X5, Fres, and ALX from MostGraph measurements results were used. The metric scores were sensitivity, specificity, accuracy and F1 score at the cut-off point defined by the maximum Youden's index, and AUC.
These values were calculated and displayed in Python program "deeplearning-5layer-down.py" available at https://github.com/sumi-yuki/mostgraph/blob/main/supplemental/deeplearning-5layer-down.py

run 1

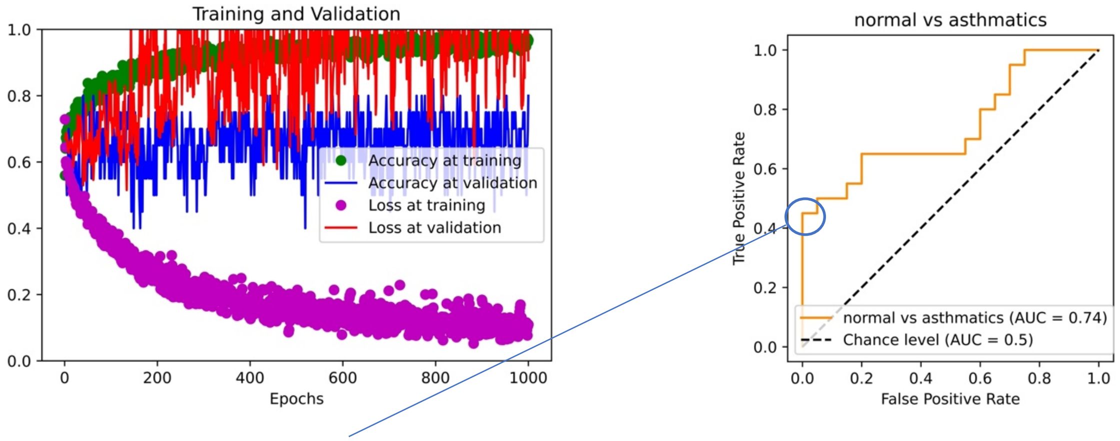

AUC 0.74, cut-off point 0.6655501, sensitivity 0.45, specificity 1.00, accuracy 0.73, F1 score 0.62

run 2

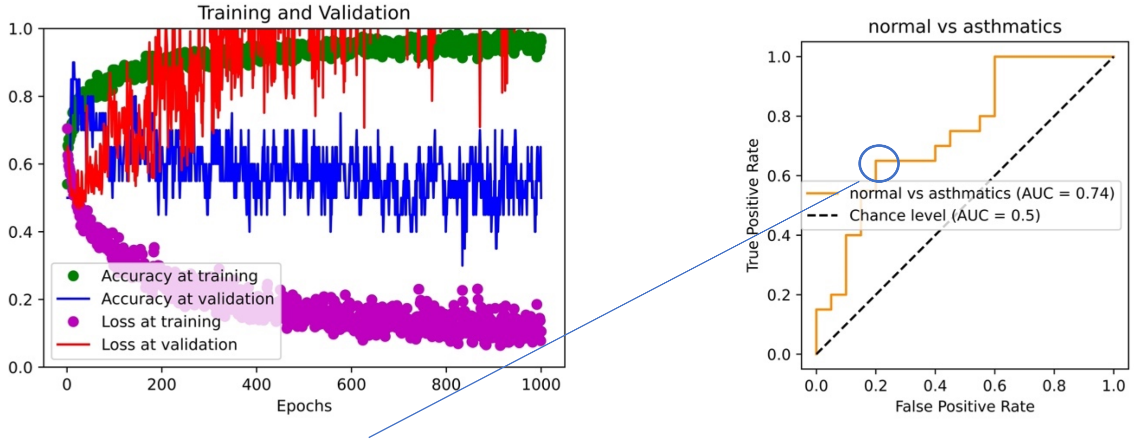

AUC 0.74, cut-off point 0.45303956, sensitivity 0.65, specificity 0.80, accuracy 0.73, F1 score 0.70

run 3

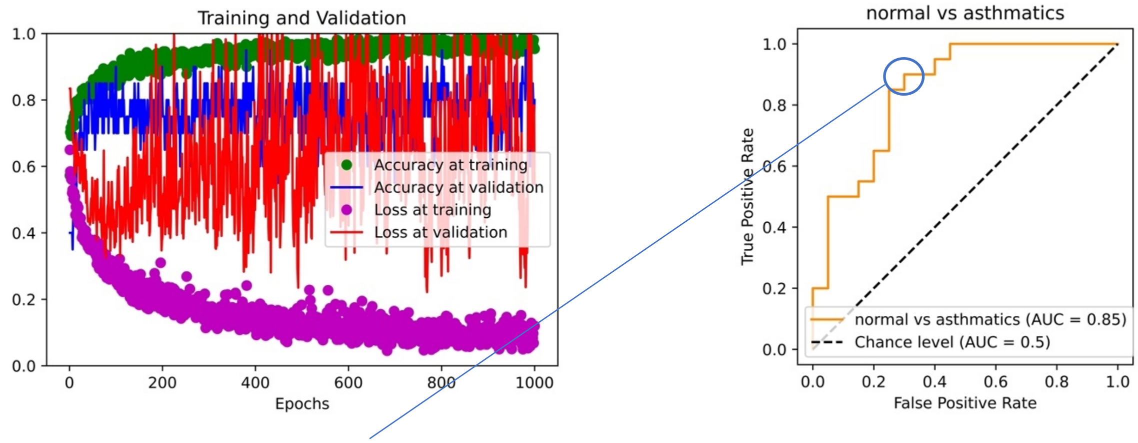

AUC 0.85, cut-off point 0.6859668, sensitivity 0.90, specificity 0.70, accuracy 0.80, F1 score 0.82

run 4

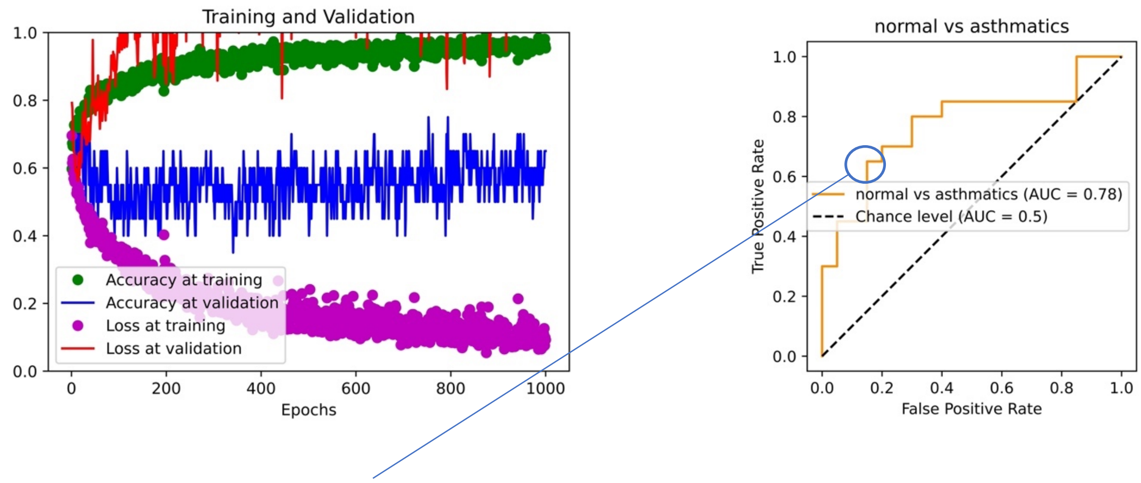

AUC 0.78, cut-off point 0.40718675, sensitivity 0.65, specificity 0.85, accuracy 0.75, F1 score 0.72

run 5

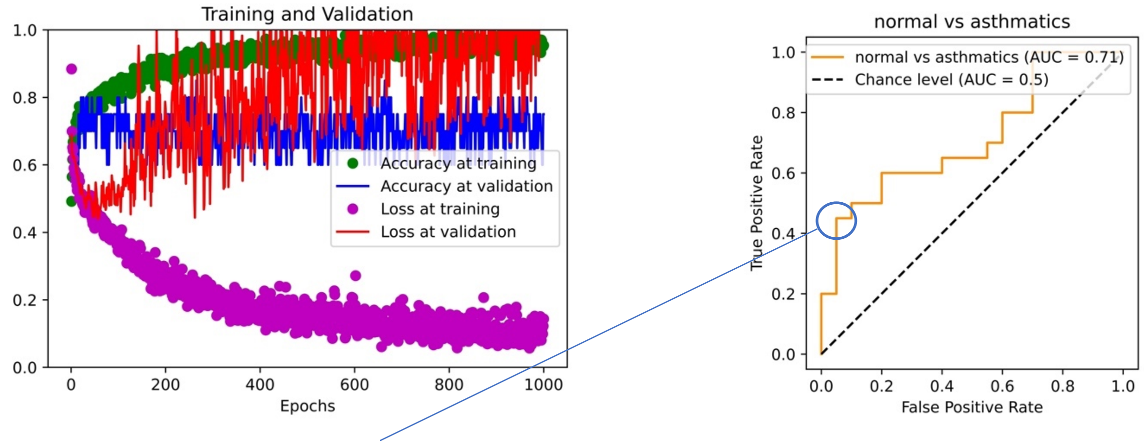

AUC 0.71, <u>cut-off point 0.89922845</u>, F1 score 0.60, sensitivity 0.45, specificity 0.95, accuracy 0.70

run 6

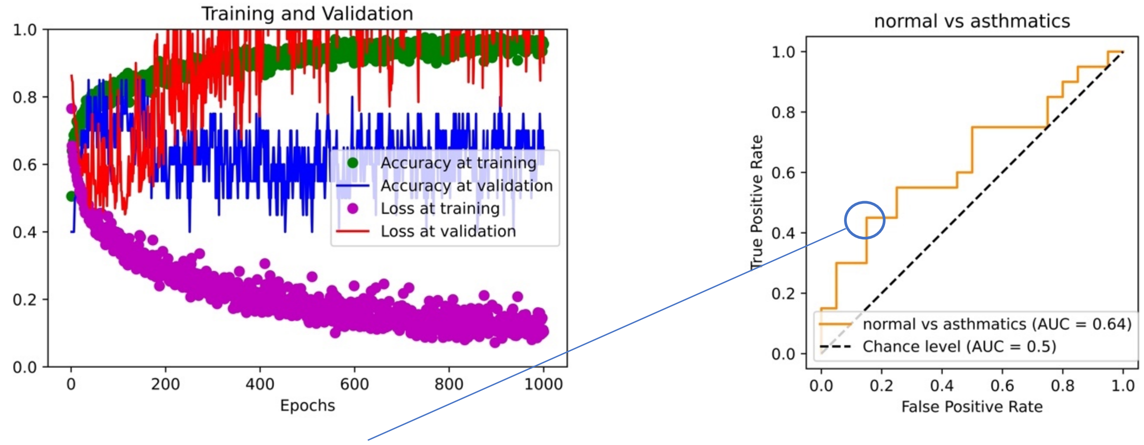

AUC 0.85, <u>cut-off point 0.38797888</u>, F1 score 0.56, sensitivity 0.45, specificity 0.85, accuracy 0.65

Supplementary Table1

Summary of various model evaluation results

The metric scores were sensitivity, specificity, accuracy and F1 score at the cut-off point defined by the maximum Youden's index, and AUC.

model	AUC	sensitivity	specificity	accuracy	F1 score
All MostGraph reported items (both sexes). Five layer deep learning model. Over-sampling with SMOTE.	0.78 ± 0.10	0.60 ± 0.19	0.90 ± 0.12	0.75 ± 0.06	0.69 ± 0.11
Values recorded for ten items during exhalation and inhalation (both sexes). Five layer deep learning model. Over-sampling with SMOTE.	0.81 ± 0.05	0.70 ± 0.10	0.87 ± 0.06	0.78 ± 0.04	0.76 ± 0.06
Values recorded for ten items during exhalation and inhalation (male). Five layer deep learning model. Over-sampling with SMOTE.	0.73 ± 0.06	0.62 ± 0.17	0.84 ± 0.11	0.73 ± 0.06	0.69 ± 0.10
Values recorded for ten items during exhalation and inhalation (female). Five layer deep learning model. Over-sampling with SMOTE.	0.73 ± 0.06	0.65 ± 0.21	0.78 ± 0.19	0.71 ± 0.02	0.66 ± 0.08
Values recorded for ten items during exhalation and inhalation (both sexes). Single layer deep learning model. Over-sampling with	0.81 ± 0.07	0.71 ± 0.17	0.88 ± 0.19	0.79 ± 0.06	0.80 ± 0.06

SMOTE.					
Values recorded for ten items during exhalation and inhalation (both sexes).<br>Logistic regression.<br>Oversampling with SMOTE.	0.71 ± 0.07	0.56 ± 0.13	0.88 ± 0.10	0.72 ± 0.03	0.66 ± 0.08
Values recorded for ten items during exhalation and inhalation (both sexes).<br>Five layer deep learning model<br>Down-sampling.	0.73 ± 0.06	0.59 ± 0.16	0.86 ± 0.10	0.73 ± 0.05	0.67 ± 0.09

Supplementary Table2

Summary of model structures and execution environments used for figures.

<table>
<tr><th></th><th>The number of neural network layers</th><th>Epochs (the number of training)</th><th>The number of test subjects in each group</th><th>The proportion for validation</th><th>Batch size</th><th>Metrics</th><th>Over-sampling / Under-sampling</th></tr>
<tr><td>Figure 2</td><td rowspan="4">5</td><td rowspan="4">2500</td><td rowspan="4">10</td><td rowspan="6">0.05</td><td rowspan="8">32</td><td rowspan="4">accuracy at cut-off point 0.5</td><td rowspan="4">5 times naive over-sampling</td></tr>
<tr><td>Table 4</td></tr>
<tr><td>Table 5</td></tr>
<tr><td>Table 6</td></tr>
<tr><td>Supplementary Figure 3</td><td>5</td><td rowspan="4">1000</td><td rowspan="4">20</td><td rowspan="4">sensitivity, specificity, accuracy and F1 score at the cut-off point defined by the maximum Youden's index, and AUC</td><td rowspan="3">SMOTE over-sampling</td></tr>
<tr><td>Supplementary Figure 4</td><td>1</td></tr>
<tr><td>Supplementary Figure 5</td><td>logistic regression</td><td>0</td></tr>
<tr><td>Supplementary Figure 6</td><td>5</td><td>0.05</td><td>under-samplimg</td></tr>
</table>
